# Translating research evidence into youth behavioral health policy and action: using a community-engaged storyboard approach

**DOI:** 10.3389/fpubh.2024.1348117

**Published:** 2024-08-21

**Authors:** McKenna F. Parnes, Merih Mehari, Georganna R. Sedlar, Cindy Trevino, Rachel Porter, Sarah C. Walker

**Affiliations:** ^1^Department of Psychiatry and Behavioral Sciences, University of Washington, Seattle, WA, United States; ^2^Northwest Kidney Centers, Seattle, WA, United States; ^3^Seattle Children's Hospital, Seattle, WA, United States

**Keywords:** co-design, youth behavioral health, community-engaged research, storyboarding, non-specialist providers

## Abstract

**Introduction:**

There is nationwide shortage of child and adolescent behavioral health providers. Lack of diversity in the mental health care profession compounds workforce capacity issues, contributing to greater disparities in treatment access and engagement for youth from historically disenfranchised communities. Strategies are needed to foster cross-sector alignment to inform policy which can improve mental health care access and reduce disparities. This current case study details a specific research-practice-policy partnership strategy, storyboarding, as a method to engage community partners in Washington State to deliberate on information drawn from research on non-specialist models of child and adolescent mental health care to support the behavioral workforce expansion.

**Method:**

Research evidence from a scoping literature review on non-specialist models of child and adolescent mental health care was shared via storyboards with community partners to inform policy efforts around the behavioral health workforce expansion. In Phase 1, community members with lived experience and clinical expertise contributed to the storyboard design process. In Phase 2, a broader community partner group shared their perspectives on the models of care presented in the storyboards via Qualtrics survey with open-ended questions. Listening sessions were also held with non-English speaking refugee and immigrant communities to elicit feedback on whether these models of care would meet their needs. Qualitative data was coded to explore emerging themes using a rapid deductive approach.

**Results:**

Community partners shared mixed responses to models of care presented from the research literature. Immigrant and refugee communities explicitly stated these existing models would not fit their context. Regarding partnership strategy success, the smaller community partner group was engaged in the storyboard design process. The broader community interacted with and provided detailed responses to the models of care presented in the storyboards. Success was also reflected in community partners’ continued participation in the next stage of the project.

**Discussion:**

Findings demonstrate how storyboarding can be effectively used to translate research evidence into accessible information to promote community partner engagement and capture community voice in policy processes. More work is needed exploring how such methods can be used to increase the use of research evidence in policy and practice spaces.

## Introduction

### Behavioral health workforce shortages and systemic violence are public health crises

The nationwide shortage of child and adolescent mental health professionals is a public health crisis. Over 160 million people in the United States live in areas with limited access to professional mental health services (1), and low availability of pediatric providers is especially dire (2). The COVID-19 pandemic has further exacerbated child and adolescent mental health problems and workforce capacity issues (3, 4). Historically disenfranchised groups, including Black, Indigenous, and People of Color (BIPOC), non-English speakers, refugee and immigrant communities, and sexual and gender minorities, including lesbian, gay, bisexual, transgender, queer, intersex, asexual, two spirit (LGBTQIA2S+) individuals, face worse barriers to care, both due to issues of provider availability as well as lack of providers who reflect the demographic and identity characteristics of these communities (5–8).

The significant demand placed on existing behavioral health providers has also led to increased burnout and an exodus of workers from such positions. In a national survey of behavioral health workers conducted by the National Council on Mental Wellbeing (9), the majority of workers reported increased caseloads and time spent on administrative tasks, as well as client severity since the COVID-19 pandemic. Nearly two-thirds of workers reported experiencing moderate to severe levels of burnout as a result. Workers expressed concerns that workforce shortages will have a negative impact on society as a whole, creating more difficulty for people seeking care to receive it and limiting the capacity of workers to continue providing care. Over 80% workers believe that public policy changes are necessary to meet the increased behavioral health demand, calling on policymakers to take action on solutions that will increase worker rates and incentivization to remain in the field, reduce burden, and expand the workforce.

Issues of behavioral healthcare access interact with social determinants of health as drivers of population health. Social determinants of health, or non-medical factors, environments, and broader systems which shape conditions of daily living, affect the resources and opportunities available to people (10). As such, while workforce shortages impact behavioral healthcare access, even when providers are available, experiences of individual and structural violence and systemic oppression affect health behaviors and openness to engage in treatment, contributing to significant health disparities (11, 12). This is particularly true in the mental healthcare system which has a longstanding history of mistreatment of racism and discrimination, resulting in appropriate mistrust among historically minoritized populations and significant barriers to seeking and engaging in care [e.g., (13)].

### Cross-sector collaboration is needed to support the behavioral health workforce

The workforce shortage paired with systemic barriers demand efforts that facilitate alignment across sectors of health care, social services, and public health to improve population health and reduce health disparities. There has been increasing communication between research and policy sectors, with research evidence being brought into policymaking processes (14, 15). However, even among successful research-policy partnerships, few approaches effectively collaborate with community and center community voice and knowledge in decision-making processes (16–18). Strategies are needed to integrate research evidence with community lived experience and expertise, as well as clinical expertise, to inform policy.

Washington State is focused on stabilizing and expanding the behavioral health workforce with a total of 33 Medically Underserved Areas and 37 Health Provider Shortage Areas (1). While some statewide initiatives in Washington have focused on training and supervising community clinicians to provide evidence-based care (19), these efforts have not addressed issues related to low provider availability and lack of a representative workforce, coupled with increased service need. In response to the state’s urgent need for more culturally responsive, effective public behavioral health services, an initiative was developed to strengthen the lived experience of a behavioral health workforce and create a culturally responsive care curriculum for implementation in Medicaid-serving organizations across the state.

While a number of influential health and public health organizations call for more meaningful collaboration among universities and the public, the science of these collaborative efforts is limited. To date, most of the examination of research-practice partnerships is in the educational sector (20, 21). Public health departments, and cross-sector collaborations to achieve public health goals present additional challenges and complexities not fully captured in the educational research on this topic. As a result, we aim to advance knowledge of the mechanisms guiding successful research-practice partnerships in public health, particularly in the science of engagement, using a case study to reflect on the use of a co-design approach to capturing community feedback on models of nontraditional mental healthcare using a participatory research approach.

### Context for the current study

Funded through the Washington state legislative proviso, the Culturally Affirming & Responsive Mental Health (CARE) for Kids & Families project addresses these public health concerns. Led by a coalition of collaborators and facilitated by the University of Washington CoLab for Community and Behavioral Health Policy (CoLab), this statewide initiative intentionally fosters research-practice-policy collaboration and communication among individuals with lived and professional expertise relevant to culturally responsive care. In order to ensure the project is informed by multiple perspectives and sources of knowledge, CoLab adopted a collaborative co-design process with broad ranging input from individuals across the state with the goals of centering community voice and increasing transparency and accountability through all stages of the process. Co-design is a participatory strategy which combines research synthesis with stakeholder expertise in policy and practice spaces to facilitate a partnership that engages end users in program/policy design processes (22).

CoLab’s approach to co-design is informed by principles of co-production, design thinking, and health services innovation, with decision making organized at four levels: the co-design team, the advisory team, the community sounding board, and the implementation team. The co-design team is a multisector group of 10 individuals representing lived experience, clinical expertise, research experience, and mental health provider experience. This team is responsible for all key decision making regarding training materials and strategy for the CARE project. The advisory team supported the co-design team, including 20 multi sector individuals representing diverse cultural groups, public mental health consumers, provider agencies, payer and state organizations, and clinical and scholarly experts. The community sounding board includes any community member across Washington State interested in sharing feedback to guide co-design team decisions. Community sounding board members were recruited via word of mouth, email, and social media, and the opportunity to sign up was available on the CARE project website through the UW CoLab. Finally, the implementation team includes four Accountable Communities of Health who are positioned to bring regional perspectives into the project. We prioritized developing trusting and mutually beneficial partnerships, working across systems, and integrating community voice with research in service of knowledge production and developing sustainable, real-world solutions.

Within this broader CARE effort, we conduct a case study of a specific research-practice-policy strategy, storyboarding, as a method to engage a wide-ranging, diverse stakeholder base to deliberate and provide responses on information drawn from the research evidence-base. Using metrics of engagement and our own analysis as participant-observers in this effort, we draw conclusions about what worked well and where efforts can be improved to foster collaborative partnerships.

## Methods and results

### Research practice partnership activities

#### Phase 1: Procedure (evidence review and storyboard development)

A key strategy in the CARE effort involved sharing research evidence on “non-specialist” mental health service models using a visual design tool called storyboarding (23, 24). Non-specialists are defined in the academic literature as experts in the community with personal experience supporting a child with mental health needs who are then supported to deliver aspects of formal mental health treatment (25, 26). The research team conducted a rapid scoping review of 573 research studies on non-specialist youth mental health service models and summarized the findings for community partners. The three predominant models presented in the academic literature included parent peers, community health workers, and youth peers. Parent peer models involved parents or caregivers who had lived experience supporting a child with mental health needs leading group or individual sessions with other parents/caregivers to provide support. Community health worker models involved training and supervising trusted local community members who may not have specific experience with mental health care to provide individual and group support to parents/caregivers and/or youth. Youth peer models involved youth aged 18 to 25 with lived experience of seeking mental health support who were trained and supervised to facilitate groups and individual sessions with adolescents. Using storyboards, each model discussed in the literature was depicted through a series of comic panels that showed either a caregiver or adolescent seeking mental health care. The storyboards included an interactive component, whereby community partners were encouraged to imagine themselves as the individual seeking care and write down what they believe a client might think or feel if they were in these situations.

Visual design tools are increasingly being used in participatory research as a strategy to enhance engagement and facilitate collaborative knowledge production (27, 28). Storyboarding allowed the research team to translate terminology and approaches from an academic perspective and communicate these concepts to community partners. One author on the team who is a clinical psychologist with 7 years of experience working with youth and families (MP) used Storyboard That, an online storyboarding tool, to create a digital story depicting the models of care from the rapid scoping review. Characters in the storyboards were intentionally created to represent community members with diverse identities in terms of age, race, gender, and ability status. The storyboard dialog was refined with members of the CARE co-design team to ensure the messages conveyed in the storyboards aligned with community priorities. The final product was shared with the broader community sounding board to invite input on what a parent/youth, administrator, or provider might think or feel in these situations, as well as what they liked about these models and ideas for alternative models of care for youth and families in Washington state.

##### Analysis (synthesis of feedback)

Qualitative data was coded by one of the authors (MP) using a rapid deductive approach guided by the Consolidated Framework for Implementation Research (CFIR) (29, 30). The first analytic step involved developing a template summary tabled with pre-specific domains based on the three models of care and key questions of interest. The feedback from community members was categorized into each of these domains. The second analytic step involved consolidating the community member feedback to identify commonly occurring themes. A matrix was created in Microsoft Excel (one tab per model and key question of interest). The matrix was designed to capture (1) broad themes or categories separated by community sounding board members taking the perspective of caregivers, children/youth, administrators, and providers, (2) within each theme a brief description of what was reported, and (3) exemplar quotes supporting the identified theme. Data from this step was used to create presentations and deliver a report to co-design team community partners.

##### Results and integration (refined storyboards)

###### Co-design team feedback

The first stage of the storyboard process involved iterating with the co-design team to align the language and information depicted in the storyboards with community priorities, as well as ensure design elements were user-friendly and accessible. The evaluation team lead (MP) attended several team meetings to start a relationship-building process with co-design team members, providing updates on the review process, including key terms used and approaches to coding the data. The review findings were then translated into storyboard drafts and shared with the co-design team. The co-design team emphasized a need for more client-centered, validating, and empathic language in the storyboards. For example, in an initial iteration of the storyboard, a caregiver is speaking to an administrator at a community mental health clinic and shares about her child and family’s need for care. The administrator responds by saying “We can help” and discusses the next stage of the process to connect with a provider. A co-design team member suggested that the administrator first validates the caregiver and expresses appreciation for their vulnerability (e.g., “Thank you for sharing, I am so sorry to hear you have been having trouble finding care. I want to make sure I can help get you to the right place.”).

Members of the co-design team also highlighted the importance of depicting a collaborative approach to working with families. Co-design members suggested language such as “I am hoping we can work as a team and come up with goals together” and “Is there anything else you want to discuss? I want to make sure this meets the needs of your family.” Such emphasis on collaboration is in contrast with many traditional approaches in mental health where a clinical provider comes in with a specific agenda or skill focus, which may not align with youth and family priorities and create barriers to developing trust in the therapeutic relationship.

The co-design team also tested various designs of the storyboards to ensure ease with providing feedback through interactive elements. The final version was presented to community members via Qualtrics, an online survey platform, with external links to storyboards fully presented on Mentimeter, an interactive presentation software. The Qualtrics survey gave community members and opportunity to share general feedback on the models of care, with questions asking what community members liked or did not like about the models and what other kinds of care models they wanted to see. The storyboards shared on Mentimeter were designed such that each page included two comic panels, with speech bubbles and thought bubbles. Users were encouraged to click on the thought bubbles which had an embedded link that took them to a separate page where they could share their perspective on what the client in the storyboard might be thinking or feeling. See Supplementary material for storyboard design elements.

#### Phase 2: Procedure (community response to storyboards)

The final product was then shared with the community sounding board. Community members who signed up for the sounding board were sent an emailing requesting participation in an online activity to inform culturally responsive care in WA. English-speaking community members responded to the activity via a Qualtrics survey with open-ended questions with as well. A separate recruitment process was conducted with non-English speaking community members to participate in listening sessions (described below). Community members who completed the Qualtrics survey were provided an external link to the full storyboards on Mentimeter asked to interact with each of the three care models (parent peers, community health workers, youth peers), and provide feedback on what they did and did not like about the models presented as well as other potential models of care. The storyboard interaction process included clicking on a thought bubble for a storyboard character and having the community member share what they believe the caregiver, youth, or administrator/provider might be thinking or feeling. Some slides had multiple thought bubbles, allowing community sounding board members to take perspectives of more than one storyboard character. Demographic data was collected on participant race/ethnicity, LGBTQIA identity, disability status, low-income status, caregiver status, and lived experience seeking and accessing mental healthcare in WA.

##### Analysis

Author (MP) coded the feedback using the rapid deductive approach from Phase 1. Themes are summarized below, and are described in more detail in Tables 1A–1C which includes the number of responses captured in each overarching theme, as well as Table 2 for quotes supporting the top three themes described.

**Table 1A:** Themes from community partner perspectives on parent peer model.

Parent peer themes	*n*	%
**Parent perspective**		
Hope and relief	43	20.1
Skepticism and lack of clarity	40	18.7
Seeking shared experience	15	7.0
Desire for immediate assistance	11	5.1
Expressing comfort with the model of care	10	4.7
Concerns about judgment	9	4.2
Feeling overwhelmed	9	4.2
Uncertainty about parent peer qualifications	6	2.8
**Child/youth perspective**		
Feeling confused and unclear about the situation	18	8.4
Focusing on play and interaction with the caregiver and provider	5	2.3
**Administrator perspective**		
Concerns about demand and capacity issues	21	9.8
Hopes that the program has tangible benefits for youth and families	3	1.4
Feeling a commitment to supporting families	2	0.9
**Provider perspective**		
Observation of family dynamics	12	5.6
Attempts to encourage and connect with families	10	4.7

**Table 1B:** Themes from community partner perspectives on community health worker model.

Community health worker themes	*n*	%
**Parent perspective**		
Hope and appreciation	42	22.8
Skepticism and concerns	33	17.9
Confusion and having more questions	21	11.4
Openness to the approach and collaboration	15	8.2
Feeling nervous with others in the group and desire for connection	12	6.5
Receiving understanding and empathy	11	6.0
Concerns about judgment	11	6.0
Worrying about lack of culturally sensitivity	10	5.4
Concerns about wait	3	1.6
**Child/youth perspective**		
Feeling confused and unsure about the approach to care	9	4.9
Observing others in the group and group dynamics	3	1.6
Feeling hopeful	2	1.1
**Administrator/provider perspective**		
Worrying about resources and capacity	5	2.7
Feeling hopeful about the group’s effectiveness	3	1.6
Reflecting on potential group dynamics and client openness	2	1.1
Questioning the resources needed to sustain and support the models	2	1.1

**Table 1C:** Themes from community partner perspectives on youth peer model.

Youth peer themes	*n*	%
**Youth perspective**		
Lack of clarity, confusion about process	26	15.8
Desire for shared experience and connection	24	14.5
Uncertainty and skepticism	21	12.7
Mixed thoughts about caregiver/parent involvement	20	12.1
Feelings of positivity and interest in receiving care	13	7.9
Feeling nervous about participating in care	10	6.1
Mixed emotions but openness to receiving care	9	5.5
Overwhelmed/disinterested	7	4.2
Feeling concerned about the wait time for group sessions to start	4	2.4
Desire to build self-awareness	2	1.2
Worrying about lack of cultural responsiveness	1	0.6
**Administrator/provider perspective**		
Concerns about youth risk, safety, and confidentiality	12	7.2
Considerations about cultural responsiveness	6	3.6
Reflections on youth treatment needs.	5	3.0
Navigating parent involvement	5	3.0

**Table 2:** Quotes reflecting top themes that emerged.

Top themes	Quotes
**Parent/youth perspective**	
Parent peer: Hope and relief	“Wow. She understands and is just like me. I feel normalized. There is hope. Maybe I can get to that point someday.”
Community health worker: Hope and appreciation	“I hope this new place works out, I appreciate getting connected with a professional to talk about my options.”
**Child/youth perspective**	
Parent peer: Feeling confused and unclear about the situation	“Who is this lady and why is she asking all these questions? Am I going to have to talk to her all the time?”
Community health worker: Feeling confused and unsure about the approach to care	“This is weird. Are we supposed to talk about our issues in front of all these parents?”
Youth peer: Lack of clarity, confusion about process	“I do not know what this role/person does.”
**Administrator/provider perspective**	
Parent peer: Concerns about demand and capacity issues	“How on earth will i pull this off without more budget and more training for new staff.”
Community health worker: Worrying about resources and capacity	“[Feeling] stressed about how to pay people enough to want to work (and stay) in community mental health.”
Youth peer: Considerations about cultural responsiveness	“I should learn more about the stigma that might exist in their culture.”

##### Results

**Community member demographics**. A total of 122 community members engaged with the storyboards and were offered a $10 gift card upon completion, with 104 who shared demographic information via the Qualtrics Survey. Community member age ranged from 18 to 69 (*M* = 34.61, *SD* = 11.41), with racial/ethnic identity including 8.7% (*n* = 9) Asian, 19.2% (*n* = 20) Black/African American, 1.0% (*n* = 1) Chicano, 3.8% (*n* = 4) Hispanic/Latino, 1.9% (*n* = 2) Mixed Race, 4.8% (*n* = 4) Native American/American Indian, 1.0% (*n* = 1) Pacific Islander, and 42.3% (*n* = 44) White. Approximately 17.3% (*n* = 18) of community members identified as LGBTQIA+. A little less than half (40.4%, *n* = 42) of community members identified as low-income and one-quarter (25%, *n* = 26) identified a person with a disability.

One-quarter (25%, *n* = 26) of community members identified as a caregiver or guardian of a person who has accessed care/services in the public behavioral health system in WA. Similarly, one-quarter (25%, *n* = 26) identified as a provider of services in the public behavioral health system in WA. Less than half the sample reported some lived experience of engaging with or attempting to access services, with 26.9% (*n* = 28) of community members reporting having first-hand experience accessing care/services and 20.2% (*n* = 21) of community remembers reporting having attempted and ben unable to access services. A small number (15.4%, *n* = 16) of community members reported having no experience interacting with the public behavioral health system in WA.

**Parent peer model**. The parent peer model demonstrated the process of a caregiver seeking help from another caregiver with lived experience. When community members took the perspective of caregivers, the following themes emerged regarding potential caregiver thoughts and feelings: (1) experiencing hope and relief; (2) feeling skepticism and lack of clarity; (3) seeking shared experience; (4) desire for immediate assistance; (5) expressing comfort with the model of care; (6) concerns about judgment; (7) feeling overwhelmed; and (8) sharing uncertainty about parent peer qualification. When community members took perspectives of children in the storyboards, two major themes emerged: (1) feeling confused and unclear about the situation and (2) focusing on play and interaction with the caregiver and provider. In taking the perspective of administrators, community members described themes regarding: (1) concerns about demand and capacity issues; (2) hopes that the program has tangible benefits for youth and families; and (3) feeling a commitment to supporting families. Finally, taking provider perspectives, themes emerged regarding: (1) observation/familiarization of family dynamics and (2) attempts to encourage and connect with families.

**Community health worker model**. The community health worker storyboards showed a caregiver seeking support from a trusted community member with lived experience. In taking caregiver perspectives, themes included: (1) experiencing hope and appreciation; (2) feeling skepticism and concerns about appropriateness of care; (3) confusion and having more questions; (4) openness to the approach and collaboration; (5) feeling nervous with others in the group and desire for connection; (6) receiving understanding and empathy; (7) having concerns about judgment; (8) worrying about lack of culturally sensitivity; and (9) expressing concerns about waiting for services. Taking child/youth perspectives, themes emerged regarding: (1) feeling confused and unsure about the approach to care; (2) generally observing others in the group and group dynamics; and (3) feeling hopeful. When community members took perspectives of an administrator, themes included: (1) worrying about resources and capacity; and (2) questioning the resources needed to sustain and support the models. Taking the provider perspective, the main themes included: (1) feeling hopeful about the group effectiveness and (2) reflecting on potential group dynamics and client openness.

**Youth peer model**. The youth peer model was intended to highlight the experience of an adolescent seeking care without caregiver/parent involvement, given that the age of consent for mental health care in Washington State is 13 years old. Thus, this model only offered opportunity for interaction as an adolescent seeking care as well as administrator/provider perspectives. In taking perspective of adolescents seeking care, themes emerged regarding: (1) lack of clarity and confusion about the process; (2) desires for shared experience and connection with peers; (3) uncertainty and skepticism; (4) thoughts about potential caregiver/parent involvement; (5) feelings of positivity and interest in receiving care; (5) feeling nervous about participating in care; (6) experiencing mixed emotions but openness to receiving care; (7) expressing overwhelm/disinterest; (8) feeling concerned about the wait time for group sessions to start; (9) sharing a desire to build self-awareness; and (10) worrying about lack of cultural responsiveness. When taking the administrator perspective, themes included: (1) considerations about cultural responsiveness and (2) reflections on youth treatment needs. When community members took the provider perspective, themes emerged regarding: (1) concerns about youth risk, safety, and confidentiality and (2) navigation around parent involvement.

**General perspectives on models of care**. After interacting with the storyboards, community sounding board members were asked if they would like to share anything about what they liked or disliked about the models presented. Overall, 61 community sounding board members shared thoughts on models of care through open-ended Qualtrics survey questions. Nearly one-third of individuals (*n* = 19, 31.1%) had a generally positive evaluation of the care models (e.g., “I think [the three models of care presented] were well thought out and thorough”). However, many individuals (*n* = 17, 27.9%) expressed remaining concerns and questions about the models of care (e.g., “It is not clear how families with a higher level of need will be addressed”). There were also feelings of appreciation expressed in community sounding board responses, particularly for the supportive connections offered through these models (*n* = 13, 21.3%; e.g., “I like how everyone’s opinion matters and they were all working together to help”). Community sounding board members also highlighted a need for more clarity around communication of the various care models (*n* = 8, 13/1%; e.g., “There is a need for more background information in the models to understand the situations better). There were also reflections regarding the cultural and linguistic responsiveness of the models (*n* = 2, 3.3%; e.g., “I liked that there are different language options available”). Finally, community sound board members (*n* = 2, 3.3%) questioned aspects of safety and confidentiality (e.g., “Are there steps in place to ensure the safety of the professionals, especially during home visits?”).

**Other potential models of care**. Community sounding board members were also asked to describe other kinds of care models they would like to see for families in Washington State. Forty-six individuals responded, offering the following alternative models of care: (1) models which focus on improving access to existing services (*n* = 12, 26.1%); (2) models which emphasize cultural fit (*n* = 9, 19.6%); (3) expansion of services and mental and behavioral health education (*n* = 7, 15.2%); (4) care for specific populations (e.g., trauma, foster care; *n* = 6, 13.0%); (5) peer and community support models (*n* = 5, 10.9%); (6) models which promote care coordination services (*n* = 3, 6.5%); (7) temporary or respite care models (*n* = 2, 4.3%); and (8) nature or art therapy models of care (*n* = 1, 2.2%).

**Listening session results**. Given the limitations of translating storyboards, a listening session guide was developed to present the various models of care to non-English speaking refugee and immigrant communities and solicit feedback and recommendations on the adaptability of those models. The guide was co-developed with a member from the co-design team who has expertise working with refugee and immigrant families in the Seattle area. The team member contacted multiple non-English-speaking community partners in King County, WA to explain the process and recruit for the listening session. This co-design team member then facilitated the listening session’s in-person with the help of cultural/language translator, providing an overview of the project, a brief summary of the models of care identified in the literature, and scenarios of different caregivers or youth seeking care from the storyboards read orally alongside images of the model. Following the listening sessions, a general discussion was held to solicit feedback, in which a vignette was shared with community members describing a process of seeking care that members from the immigrant and refugee community may experience, along with questions about resources they think are needed as well as challenges they have experienced in similar situations.

The team specifically engaged non-English speaking communities in the Seattle area who have broad representation of mental health needs and barriers to accessing care through traditional mental health services. The first listening happened with Somali speaking refugees and immigrants from Somalia residing in South King County, Seattle, WA, which has a high percentage of immigrant and refugee populations. The session happened in person at the Somali community center venue and participants were offered $ 50 gift cards as compensation. Somali community members reported back to the facilitator that the models and scenarios were not designed for them. Among the reasons mentioned include they would only feel comfortable discussing mental health issues with someone with the credentials and awareness of their cultural and religious values. Second, they expressed that members of their community did not have the knowledge or information about mental health and when to seek help, which served as an additional barrier to them actively engaging with the models of care presented during the listening sessions.

### Success of the partnership strategy

When considering outcomes and impacts of the participatory storyboard approach (21), evidence from the partnership process suggests this strategy was effective for fostering research-practice-policy partnership. Our approach involved translating research evidence into a visual tool that allowed community partners to engage in the application of the research to inform policy development. Drawing on Drahota et al. (20) review, which discusses facilitating and hindering factors to the partnership process, the research team intentionally took care to build trust and respect with co-design team partners, which encouraged transparent and generative conversations about the storyboard designs. This process resulted in storyboards which represented the research literature and used language from individuals with lived experience that could be presented to the broader community. This strategy for combining research and community perspectives translated into a final product that aligned with co-design team values and, in turn, was well received by the broader community.

Metrics for success were also reflected in the number of community partners who engaged with the storyboards, both during the design process and when soliciting broader community feedback. Of the 10 co-design team members, 80% (*n* = 8) assisted in the storyboard design process during a synchronous session, while 30% of team members provided additional contributions outside of a co-design session. In terms of broader community engagement, of the 189 community sounding board members, 64.5% (*n* = 122) interacted with the storyboards on Mentimeter by taking the perspective of a caregiver, youth, and/or administrator. The parent peer storyboard had the highest level of interaction with 214 responses, while the community health worker model had 184 responses, and the youth peer model had 165 responses. The community listening session with refugee and immigrant community was also successful, in that the models of care presented in the storyboards were able to be translated and shared with 6 community partners, 5 of whom had limited or no English proficiency.

Success of this partnership strategy is also demonstrated in the next steps for the CARE project, which includes continued community participation in workforce training and policy recommendations. Advisory and co-design team members have now formed subcommittees to develop and pilot trainings for mental health agency staff over the next year. The CARE team also put out a request for proposals to contract with a community organization in Washington State that will partner in this effort to train clinicians in culturally responsive care.

## Discussion

Overall, this paper describes a detailed process of facilitating research-practice-policy collaboration to inform the development of training resources and a training strategy to improve the cultural responsiveness of public mental health services for youth and families in Washington State. Specifically, a collaborative partnership was developed between the CARE project research team and the co-design team to create materials for the broader community to promote community representation. The results of this project highlight how visual design tools can be used as one method of translating research evidence into accessible information to meaningfully engage community members and elicit community perspectives. The visual participatory methods fostered strong community partner engagement, with the majority of co-design team members providing feedback on the storyboard design process as a way to deliberate on the ‘evidence-base’ and more than two-thirds of community sounding board partners interacting with storyboards. This high engagement offered insights and rich data that will directly inform policy directions for the CARE project. Of note, there was greater engagement with the parent peer model and community health worker model compared to the youth peer model, which may reflect respondent preferences for engaging with storyboards they found to be most relatable or interesting. The use of open-ended questions may have also posed as a limitation, given that open-ended questions have higher rates of nonresponse as compared to other question formats (e.g., yes/no, ordinal scales, nominal scales) (31). Future research should collect both quantitative and qualitative data to increase the likelihood of response and provide a more complete picture and deeper understanding of the data.

While the benefits of such design tools for facilitating research-practice-policy partnerships are evident, it is important to acknowledge limitations of such an approach. Notably, there was higher engagement from the co-design team in session than out of session in the storyboard design process, suggesting challenges with engaging community experts asynchronously, which may add barriers to community partner collaboration if synchronous meetings are not possible. Additionally, even after engaging co-design team members in the design process to ensure the storyboards were clear and accessible, many community partners from the sounding board still expressed confusion and lack of clarity around the models of care. Future approaches to storyboarding may benefit from an initial statement providing more details to ensure community members fully understand information being presented. Additionally, the use of a visual design tool limits engagement of individuals who are blind or visually impaired, calling for a multi-modal approach to increase accessibility through other assistive technologies. Moreover, given the depiction of these models via comic panels, there are limitations in storytelling detail and clarity, which could have been addressed with other approaches (e.g., video with visuals and sound).

It is also important to note that the storyboards were only presented to English-speaking members of the community, which is not representative of all youth and families in need of care across Washington State. Elements of the storyboards have been translated into materials for non-English speaking refugee and immigrant community members to provide feedback via community listening sessions (i.e., facilitated discussions to collect community perspectives and ideas about these models of care). While data collection on perspectives on non-English speaking refugee and immigrant community members is ongoing, our research team was limited in not being able to directly translate the storyboards to gather the same level of interaction. Due to the fact the storyboards are primarily designed with one cultural context in mind, even when translated to another language they may not have the same meaning as the original context, and therefore would not communicate the intended message. However, we did elicit clear feedback from the immigrant and refugee community that without strong resources and capacities for their own community members to serve as care providers, these models would not work.

Finally, despite being an effective strategy for translating research evidence, storyboards and other visual design tools are limited in that they may not be able to communicate breadth or depth of research available. Research teams are often making decisions about the evidence that will be included and shared with community partners, and may have blind spots as to what community members think is relevant. Involving community members in the research evidence gathering and synthesis process may address some of these limitations, as community members will have even more say in guiding project direction.

Despite these limitations, this project highlights the benefits of using visual participatory approaches to promote successful research-practice-policy partnerships. The storyboards allowed the research team to work closely with community members with lived experience and clinical practice lenses to shift models of care presented in the academic literature to best reflect the wants and needs of Washington youth and families. There is potential for this method to be used in other contexts, such as creating training materials for non-specialist providers, which was a suggestion offered by one co-design team member. Visual design tools could also be used more broadly for visual science communication in public health contexts, offering opportunities for community members to co-design public health messages and infographics to support more effective messaging. Future research should continue to explore how such methods can be used to increase the use of research evidence in policy and practice spaces and create partnerships across multiple diverse sectors.

## Data Availability

The raw data supporting the conclusions of this article will be made available by the authors, without undue reservation.
