# Supplementary material for: Translating research evidence into youth behavioral health policy and action: using a community-engaged storyboard approach
Source: Front Public Health. 2024 Aug 21;12:1348117. doi: 10.3389/fpubh.2024.1348117 (PMC11371670; doi:10.3389/fpubh.2024.1348117)

Supplementary material: Storyboards designed and shared with community partners

# Peer-to-Peer Support Model

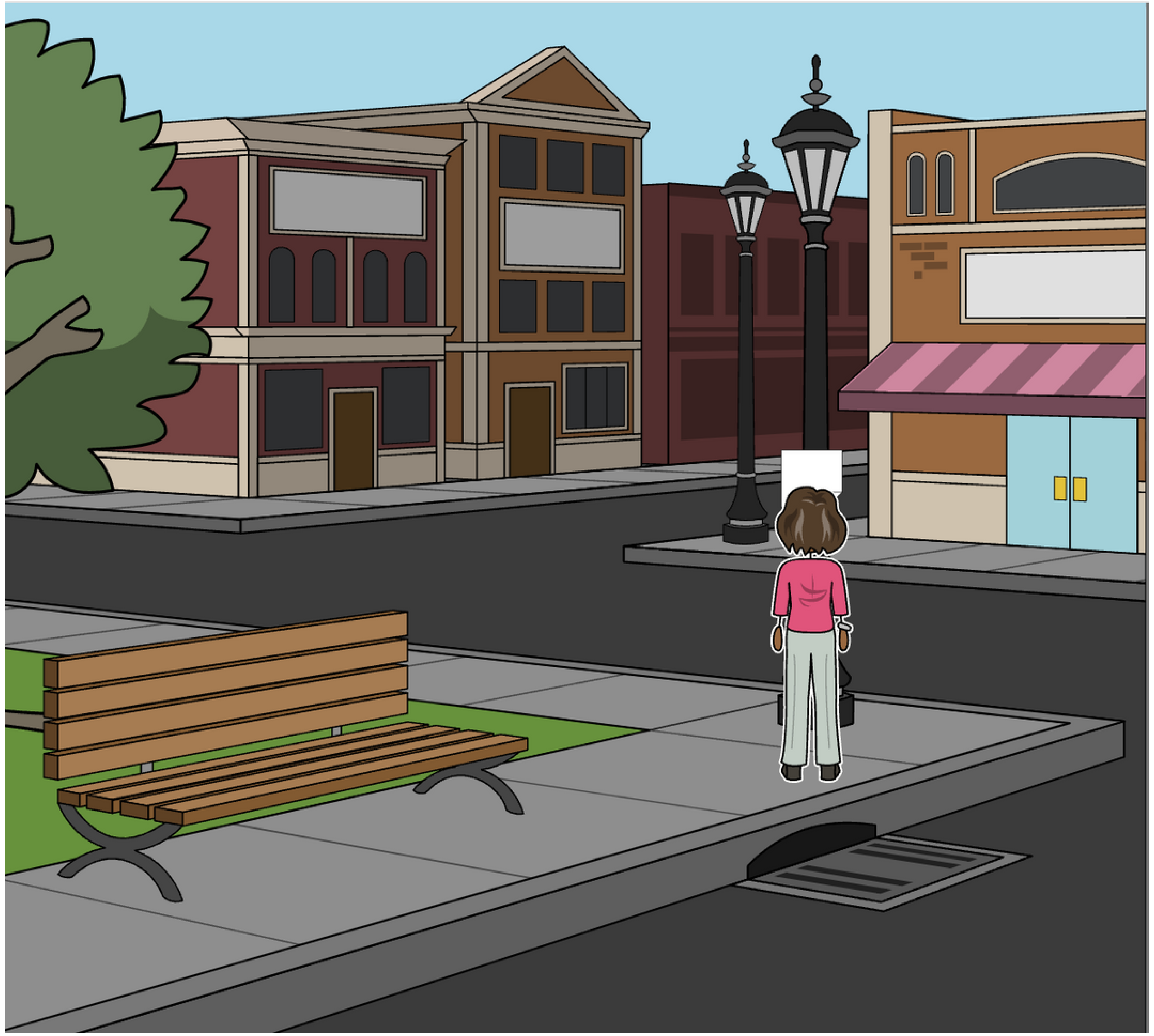

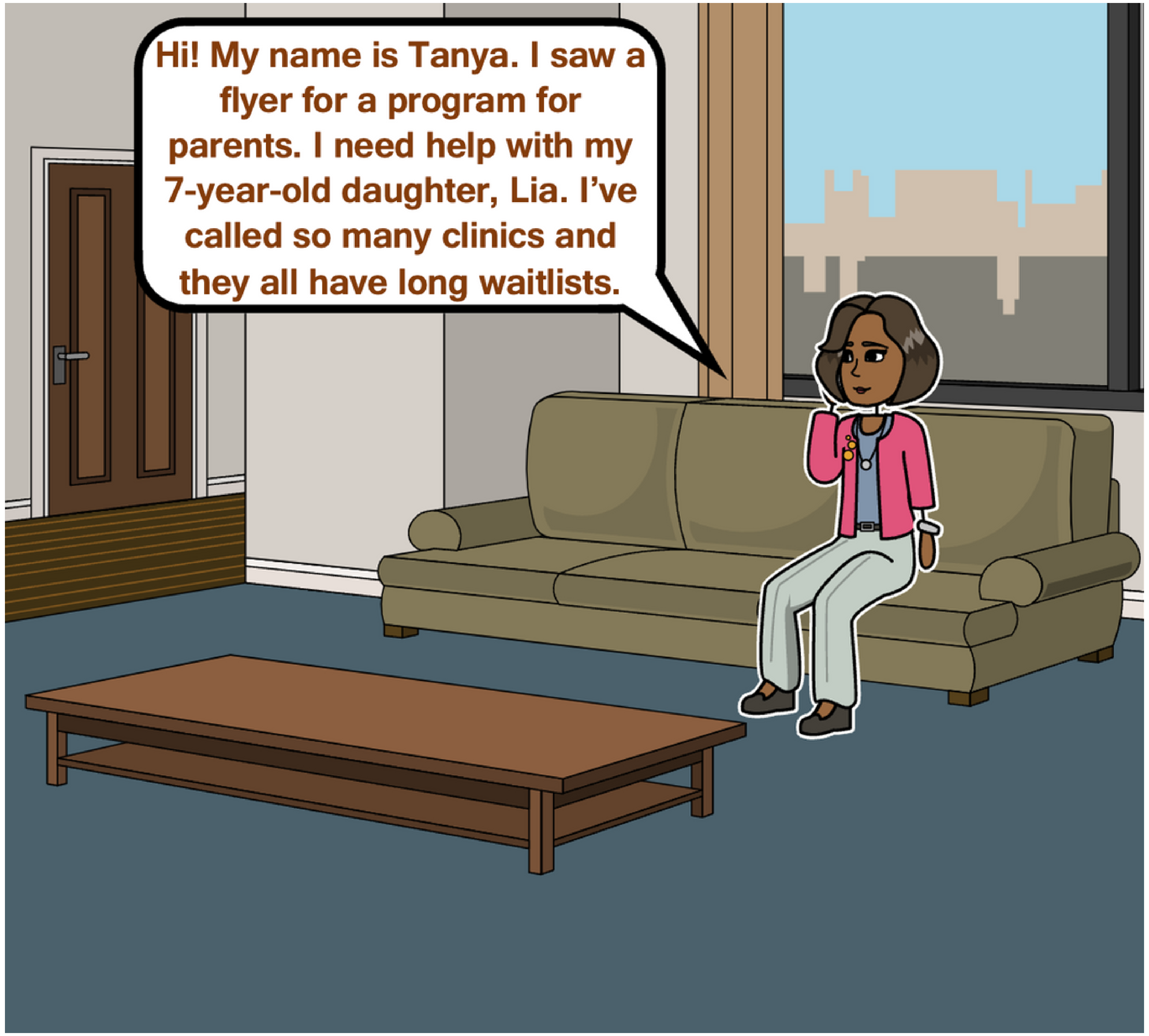

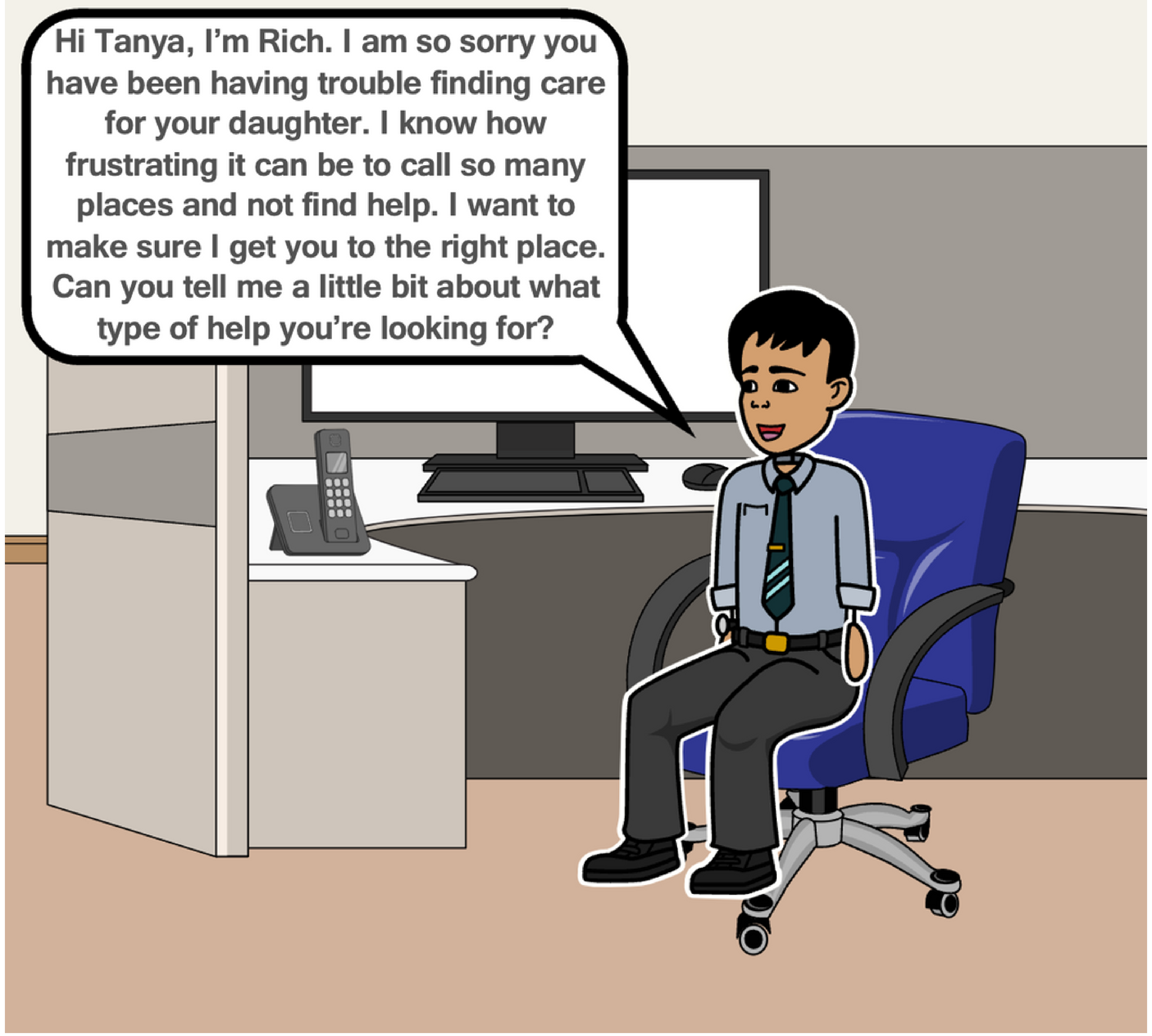
Hi Tanya, I'm Rich. I am so sorry you have been having trouble finding care for your daughter. I know how frustrating it can be to call so many places and not find help. I want to make sure I get you to the right place. Can you tell me a little bit about what type of help you're looking for?

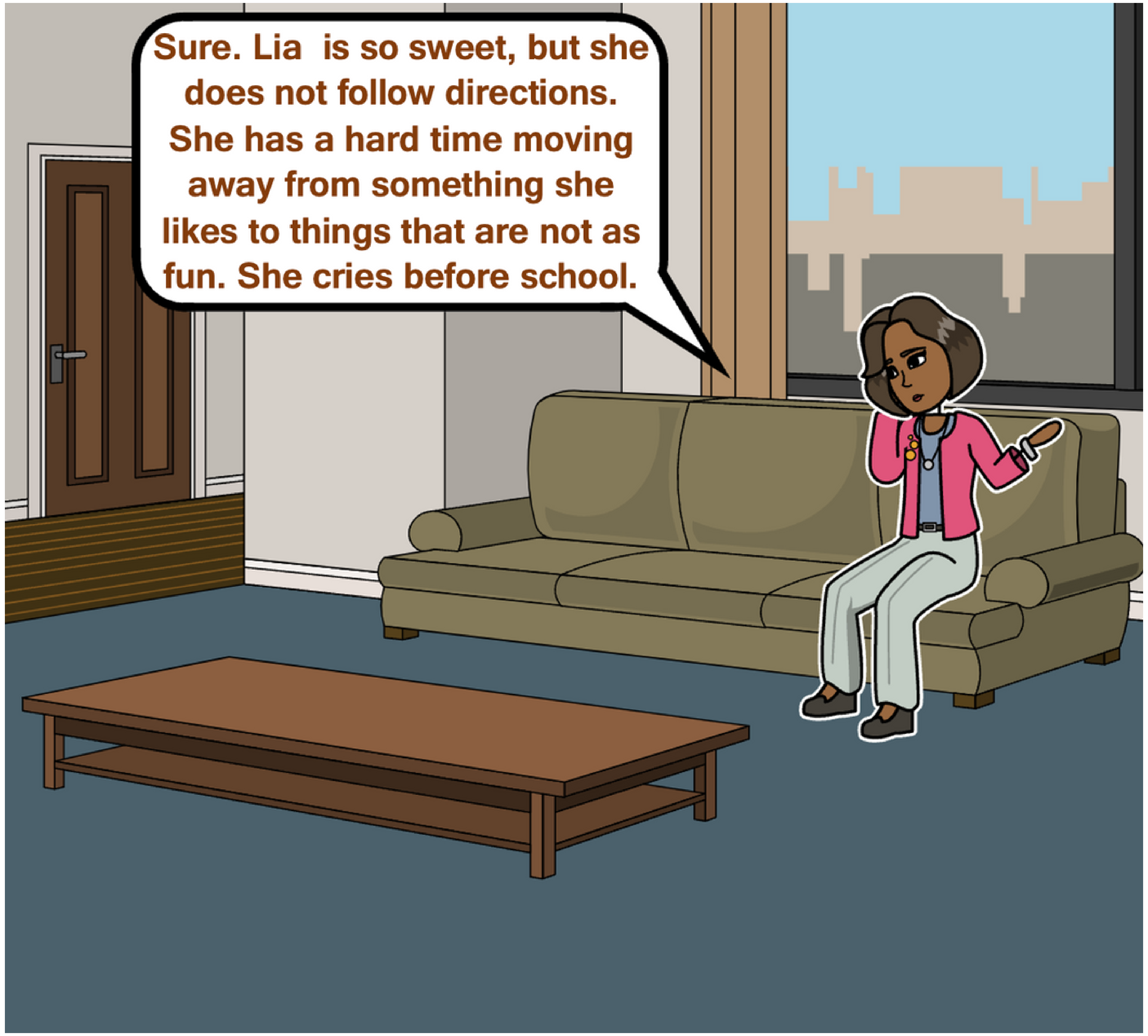
Sure. Lia  is so sweet, but she does not follow directions. She has a hard time moving away from something she likes to things that are not as fun. She cries before school.

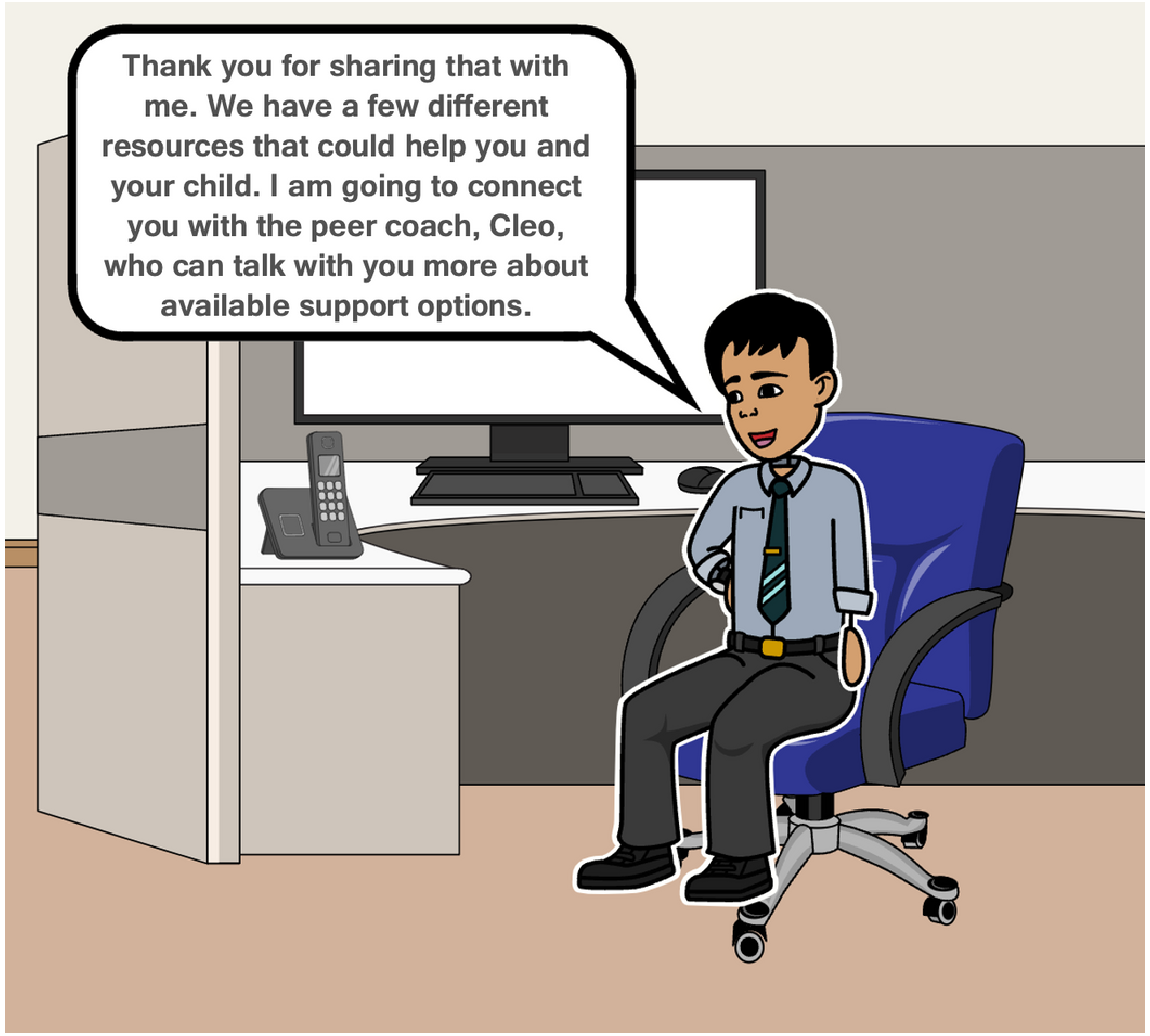
Thank you for sharing that with me. We have a few different resources that could help you and your child. I am going to connect you with the peer coach, Cleo, who can talk with you more about available support options.

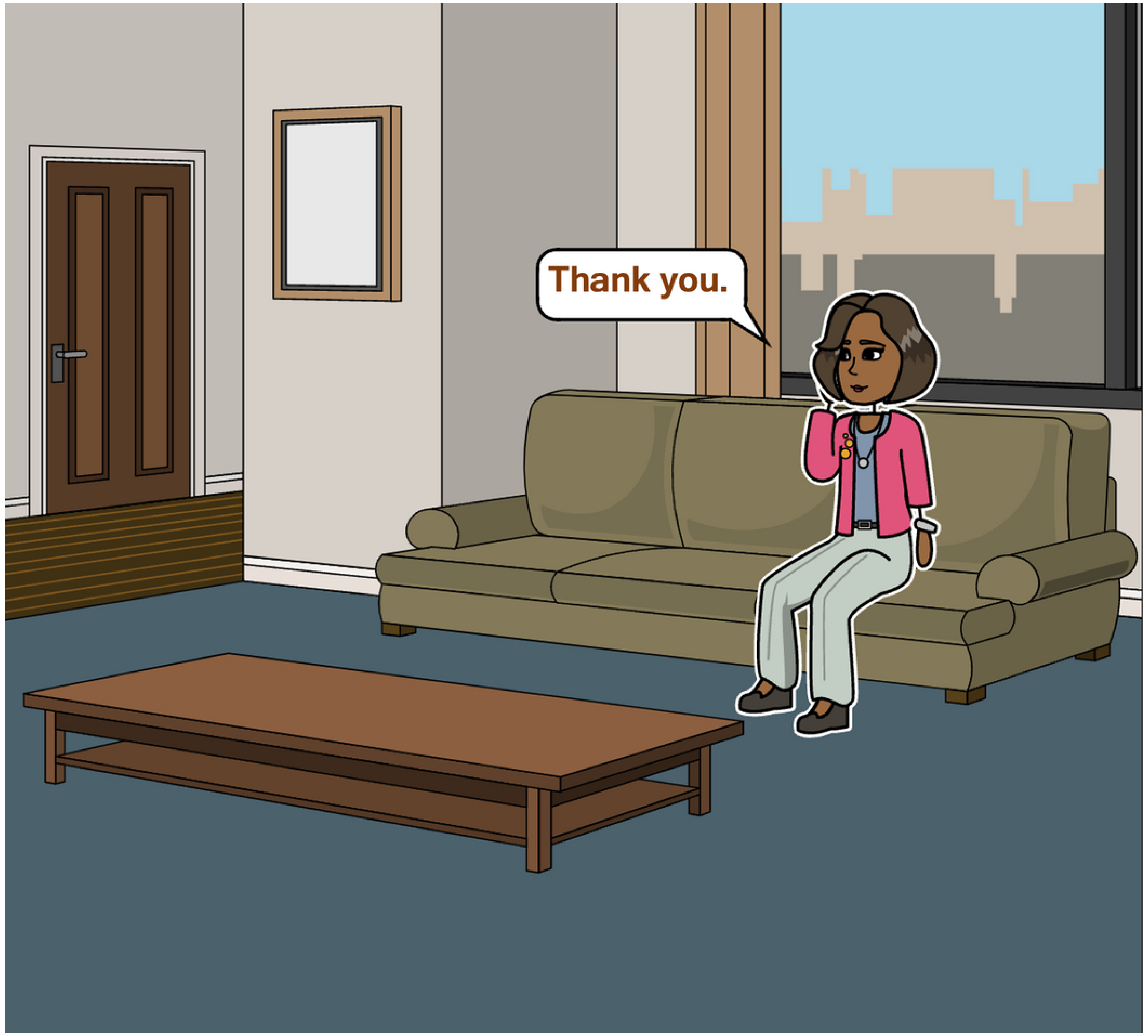
Thank you.

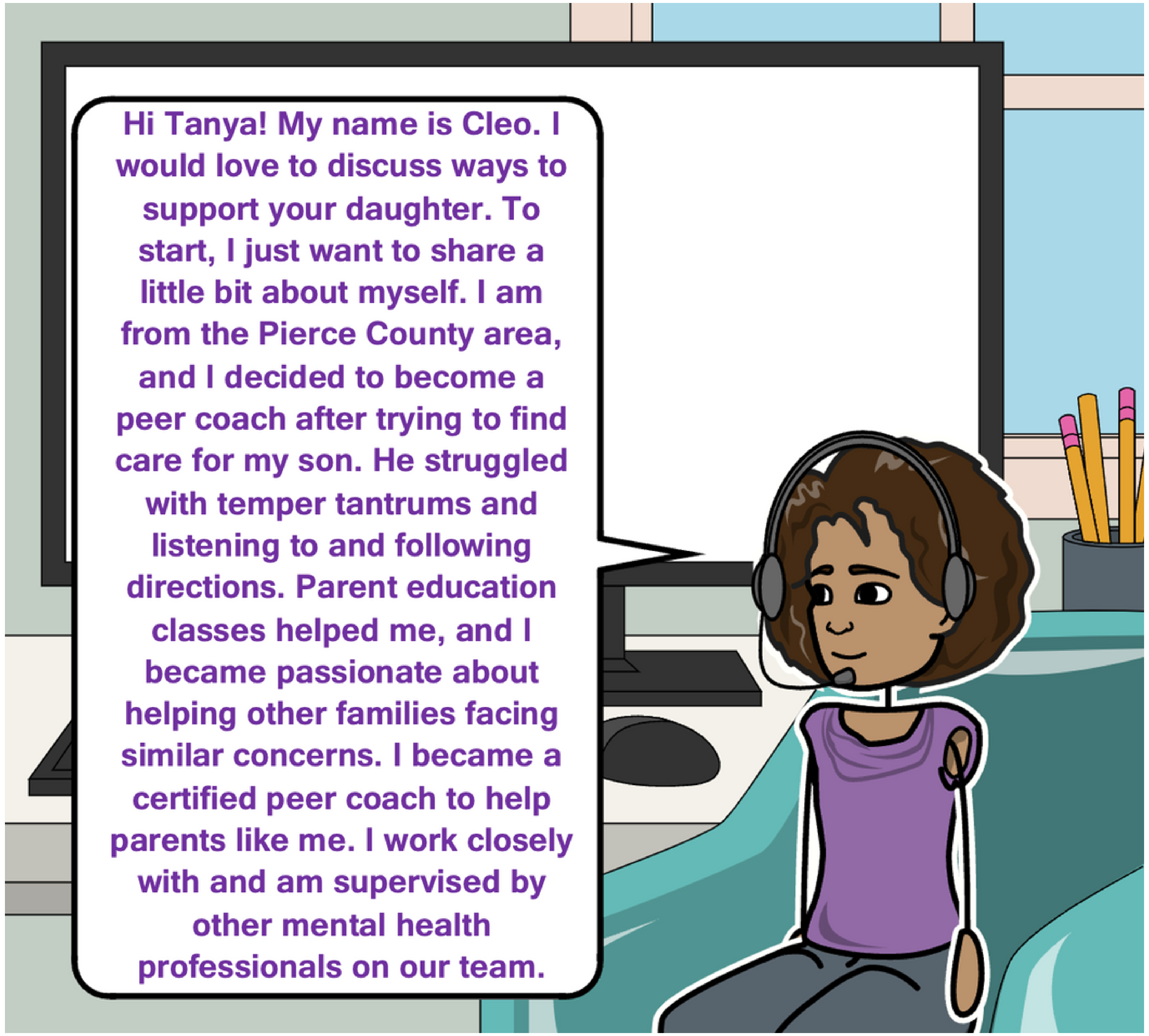
Hi Tanya! My name is Cleo. I would love to discuss ways to support your daughter. To start, I just want to share a little bit about myself. I am from the Pierce County area, and I decided to become a peer coach after trying to find care for my son. He struggled with temper tantrums and listening to and following directions. Parent education classes helped me, and I became passionate about helping other families facing similar concerns. I became a certified peer coach to help parents like me. I work closely with and am supervised by other mental health professionals on our team.

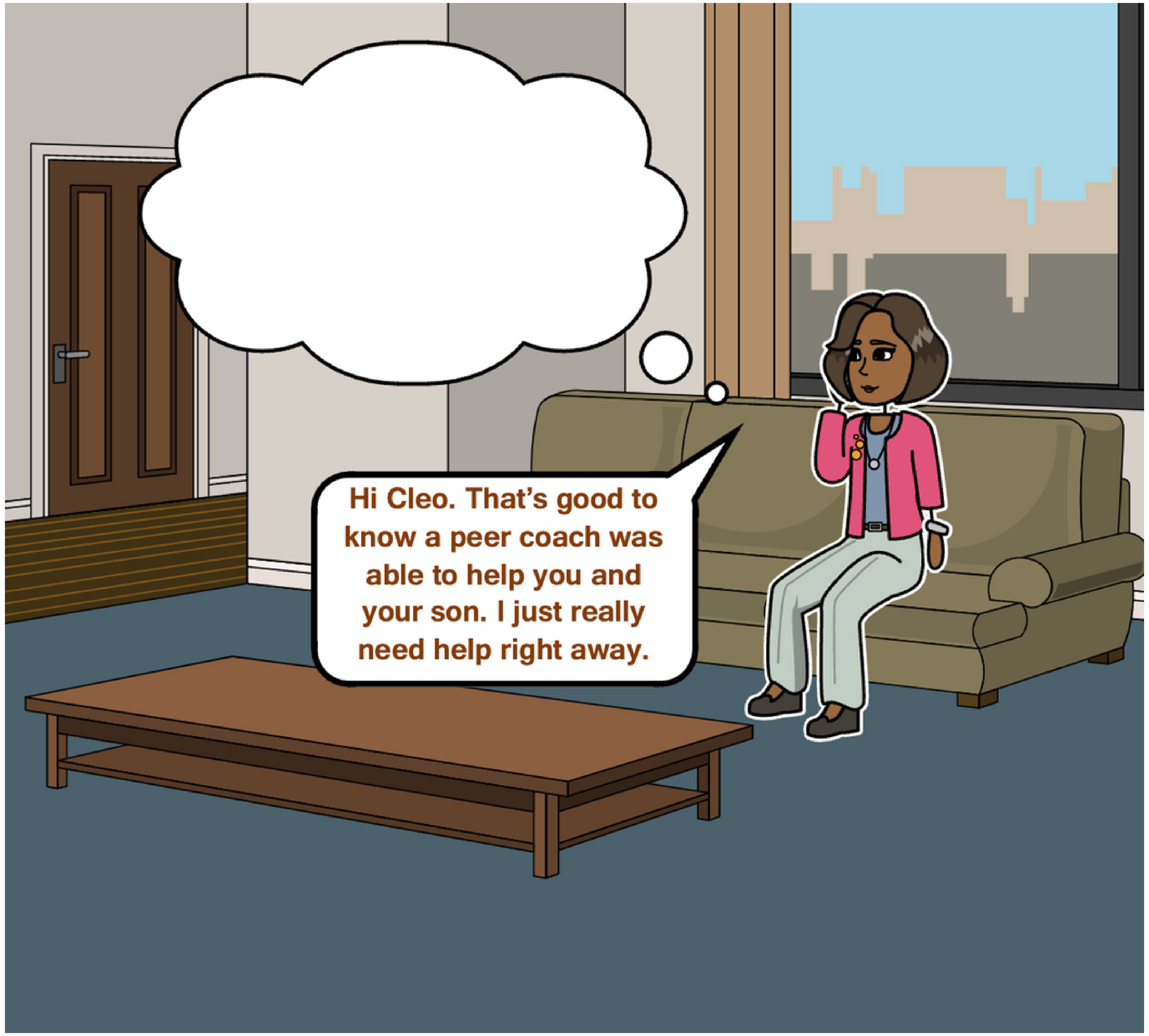
Hi Cleo. That’s good to know a peer coach was able to help you and your son. I just really need help right away.

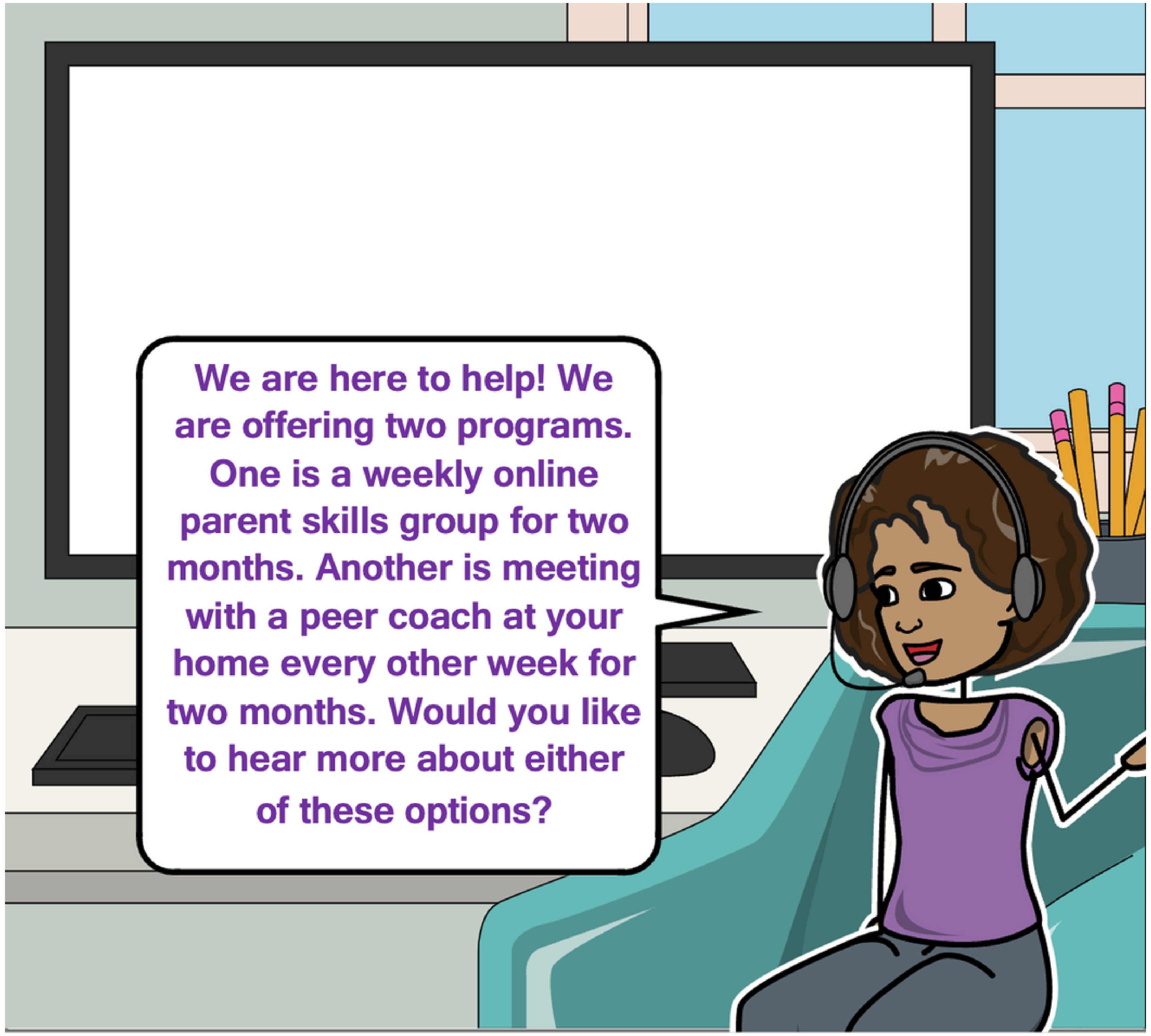
We are here to help! We are offering two programs. One is a weekly online parent skills group for two months. Another is meeting with a peer coach at your home every other week for two months. Would you like to hear more about either of these options?

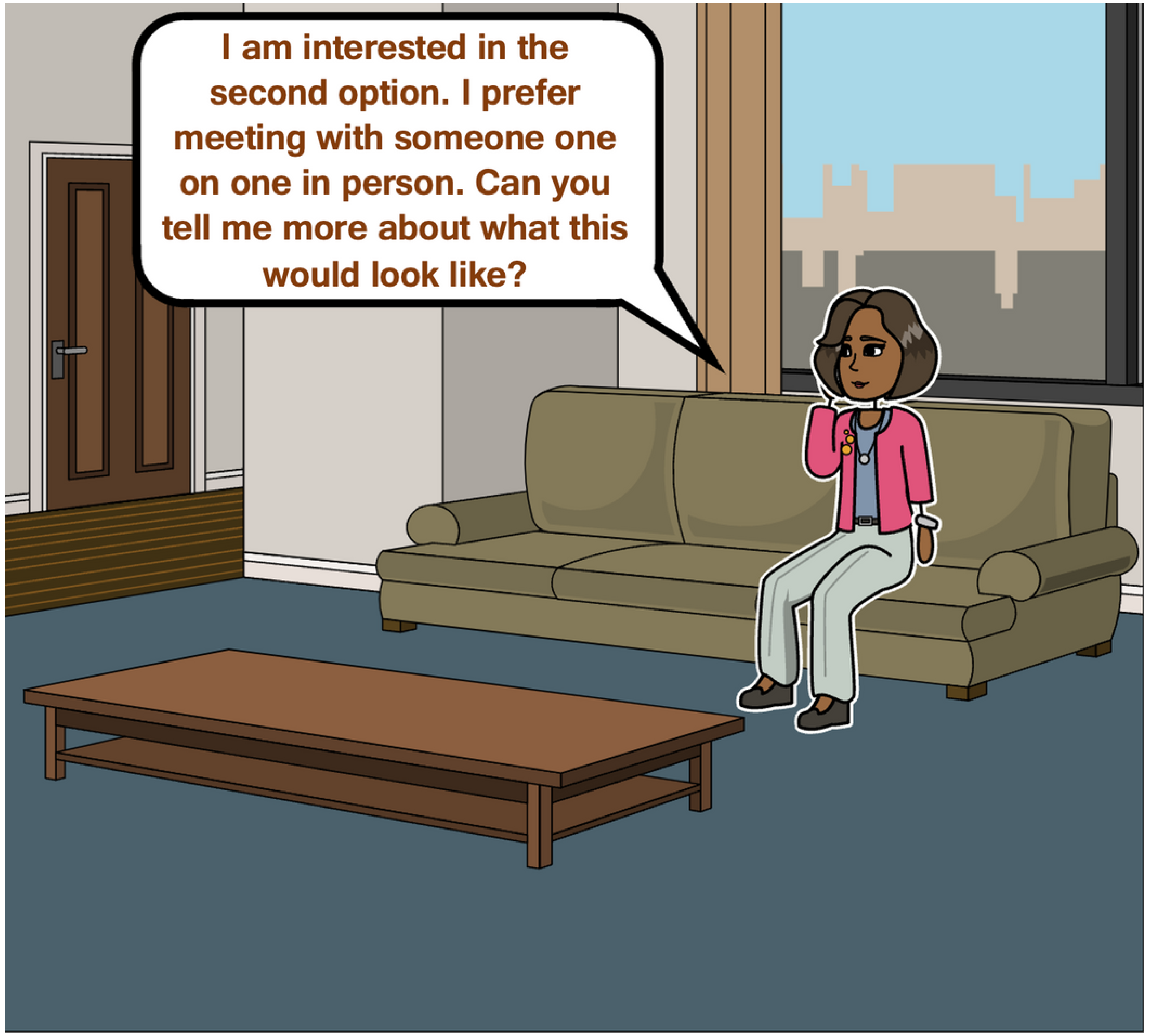
I am interested in the second option. I prefer meeting with someone one on one in person. Can you tell me more about what this would look like?

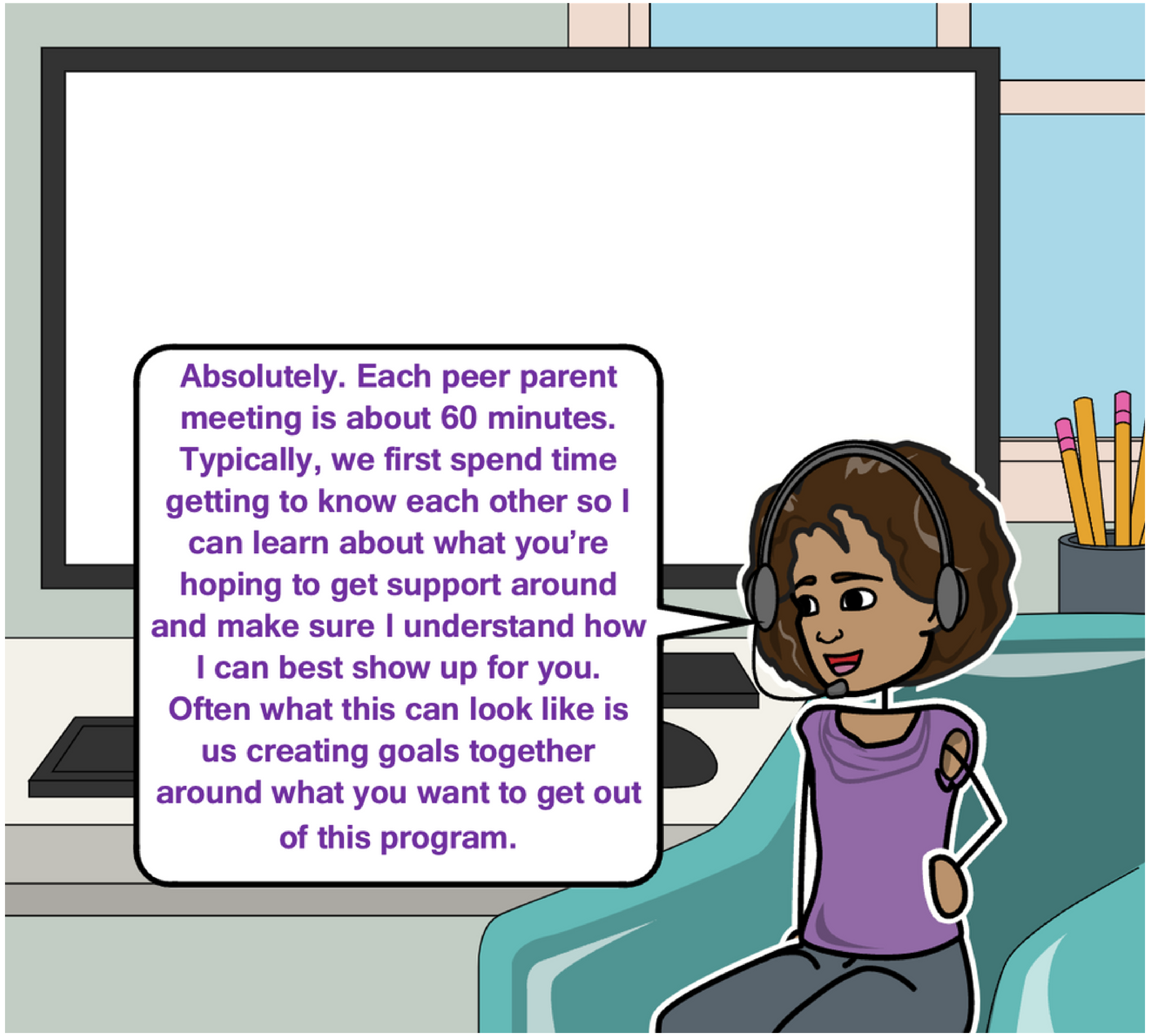
Absolutely. Each peer parent meeting is about 60 minutes. Typically, we first spend time getting to know each other so I can learn about what you're hoping to get support around and make sure I understand how I can best show up for you. Often what this can look like is us creating goals together around what you want to get out of this program.

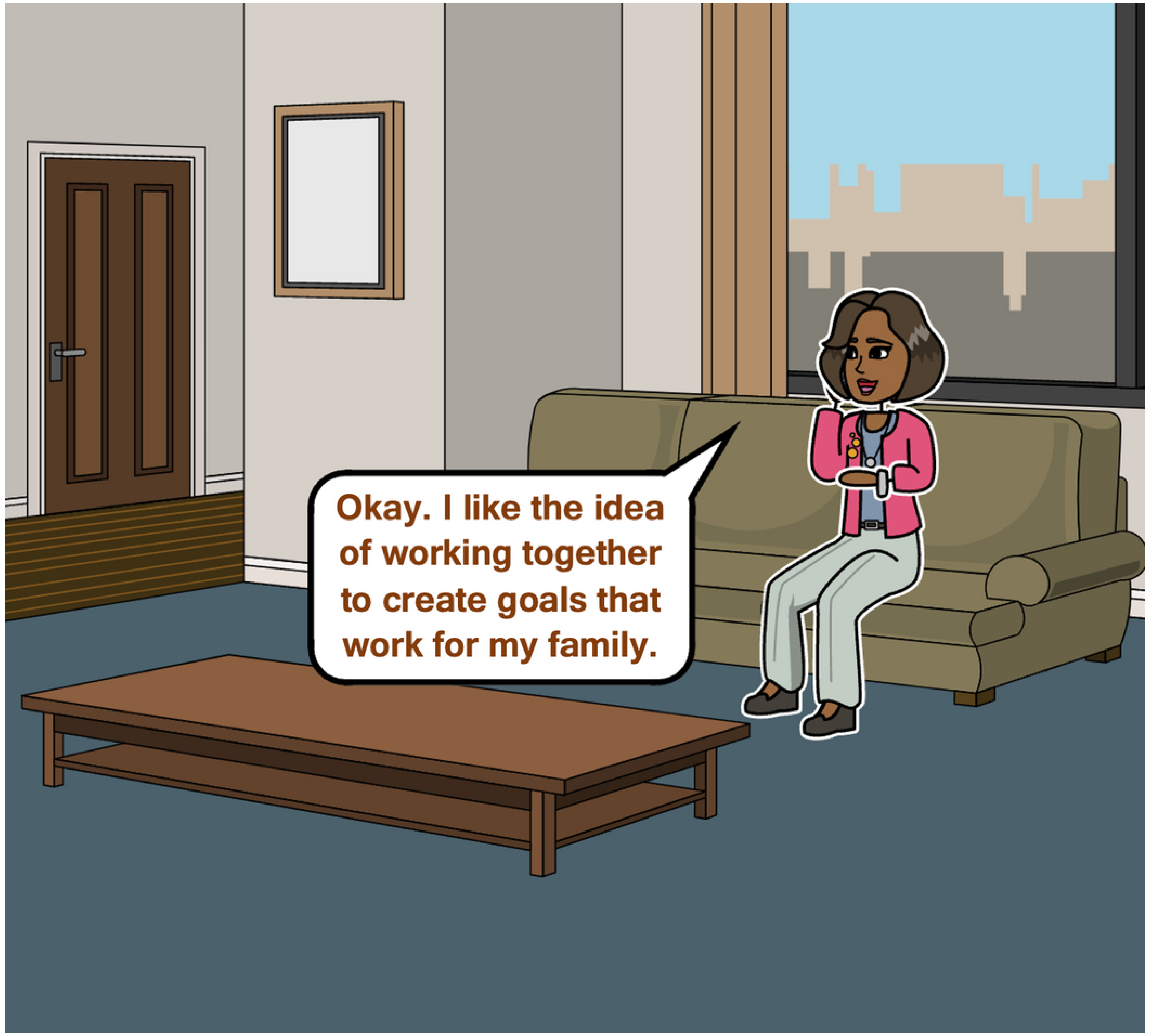
Okay. I like the idea of working together to create goals that work for my family.

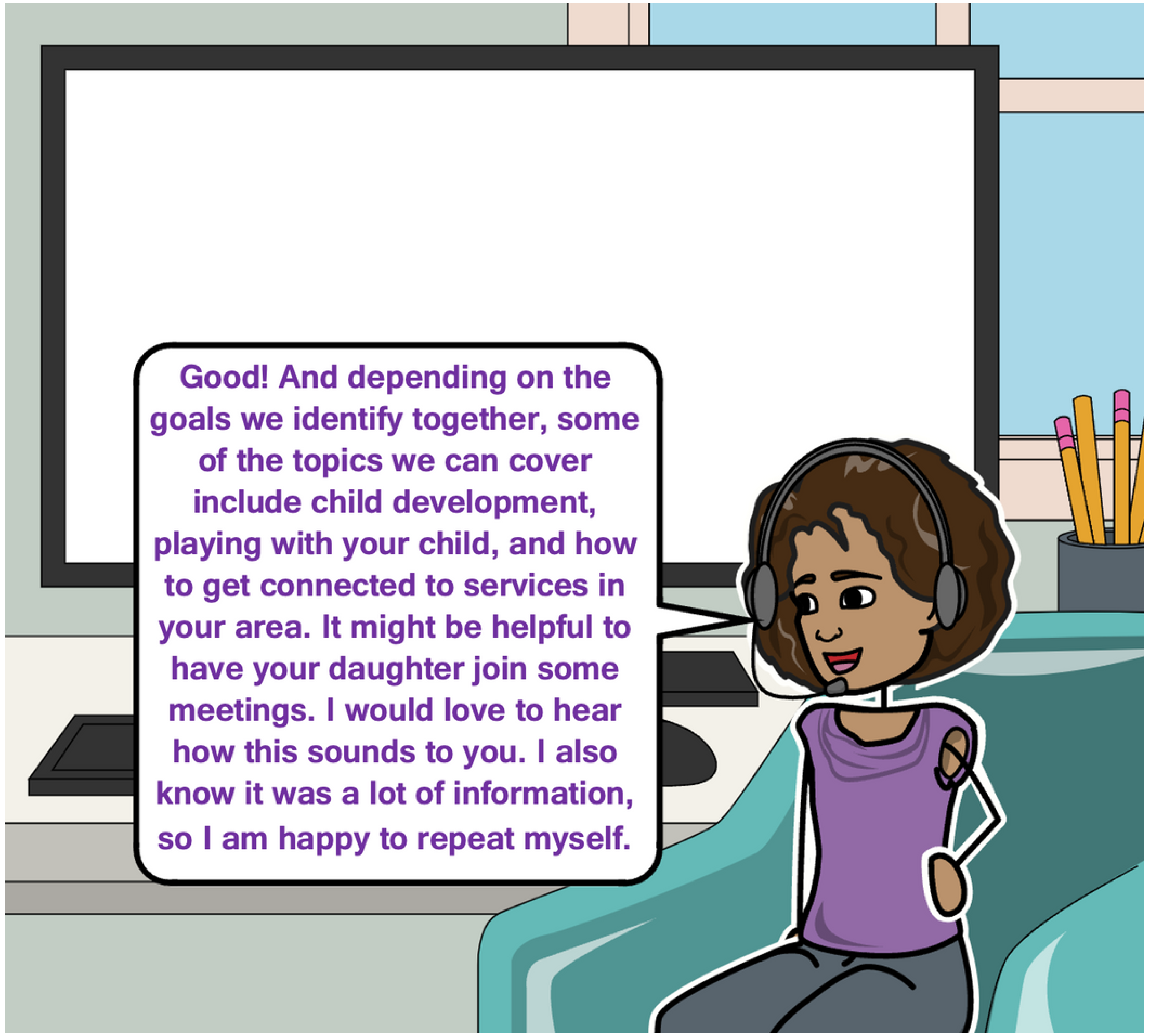
Good! And depending on the goals we identify together, some of the topics we can cover include child development, playing with your child, and how to get connected to services in your area. It might be helpful to have your daughter join some meetings. I would love to hear how this sounds to you. I also know it was a lot of information, so I am happy to repeat myself.

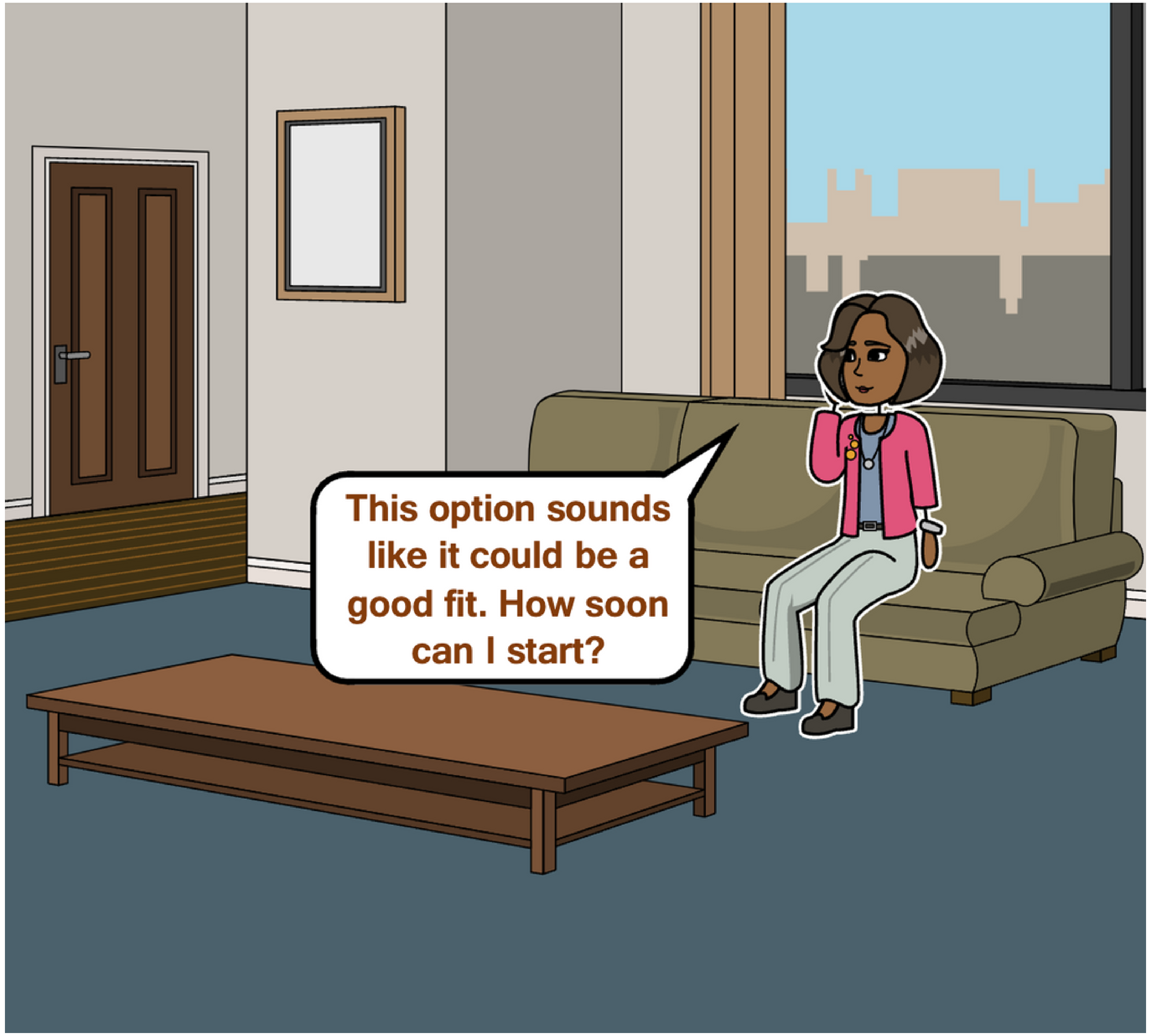
This option sounds like it could be a good fit. How soon can I start?

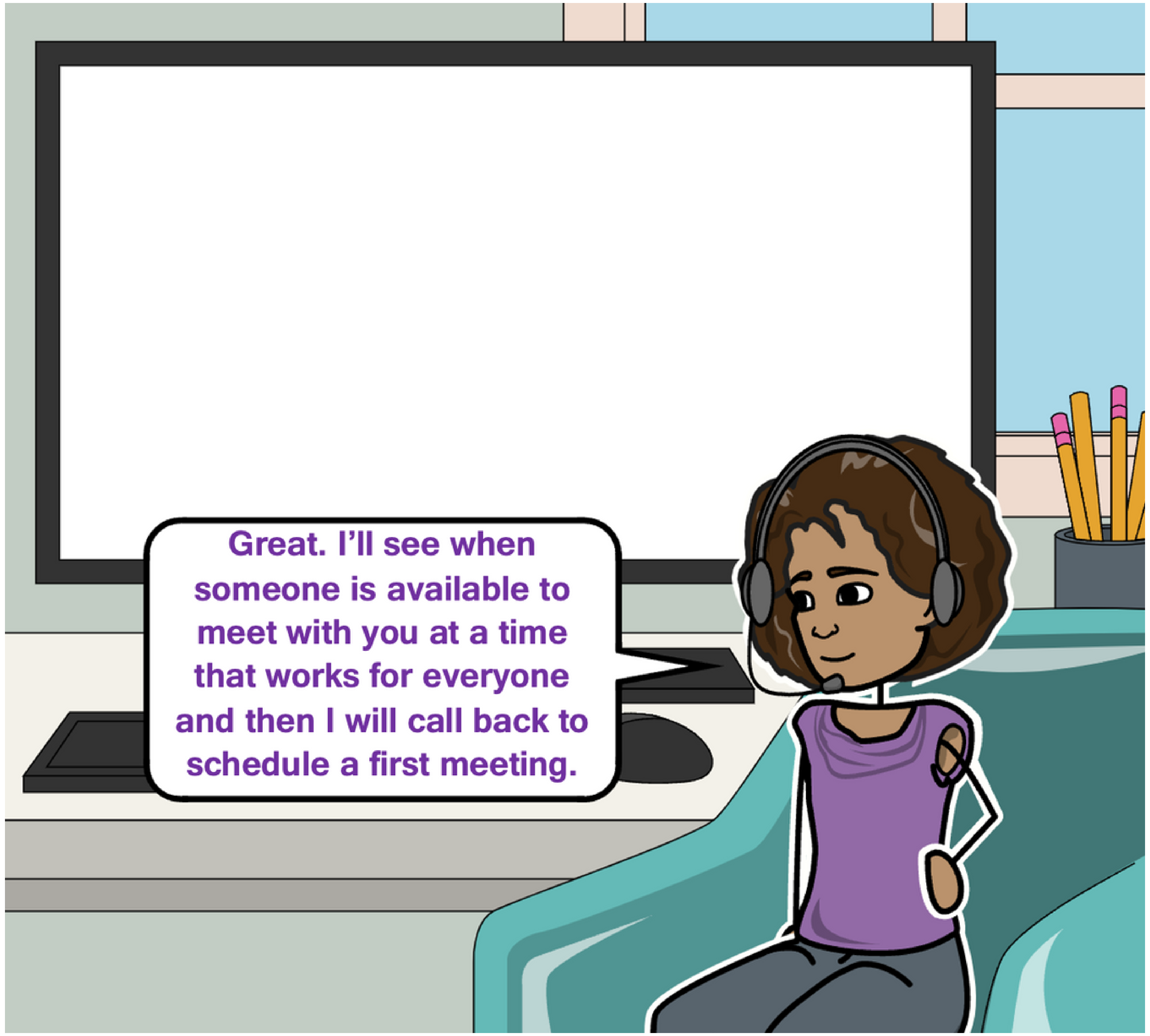
Great. I'll see when someone is available to meet with you at a time that works for everyone and then I will call back to schedule a first meeting.

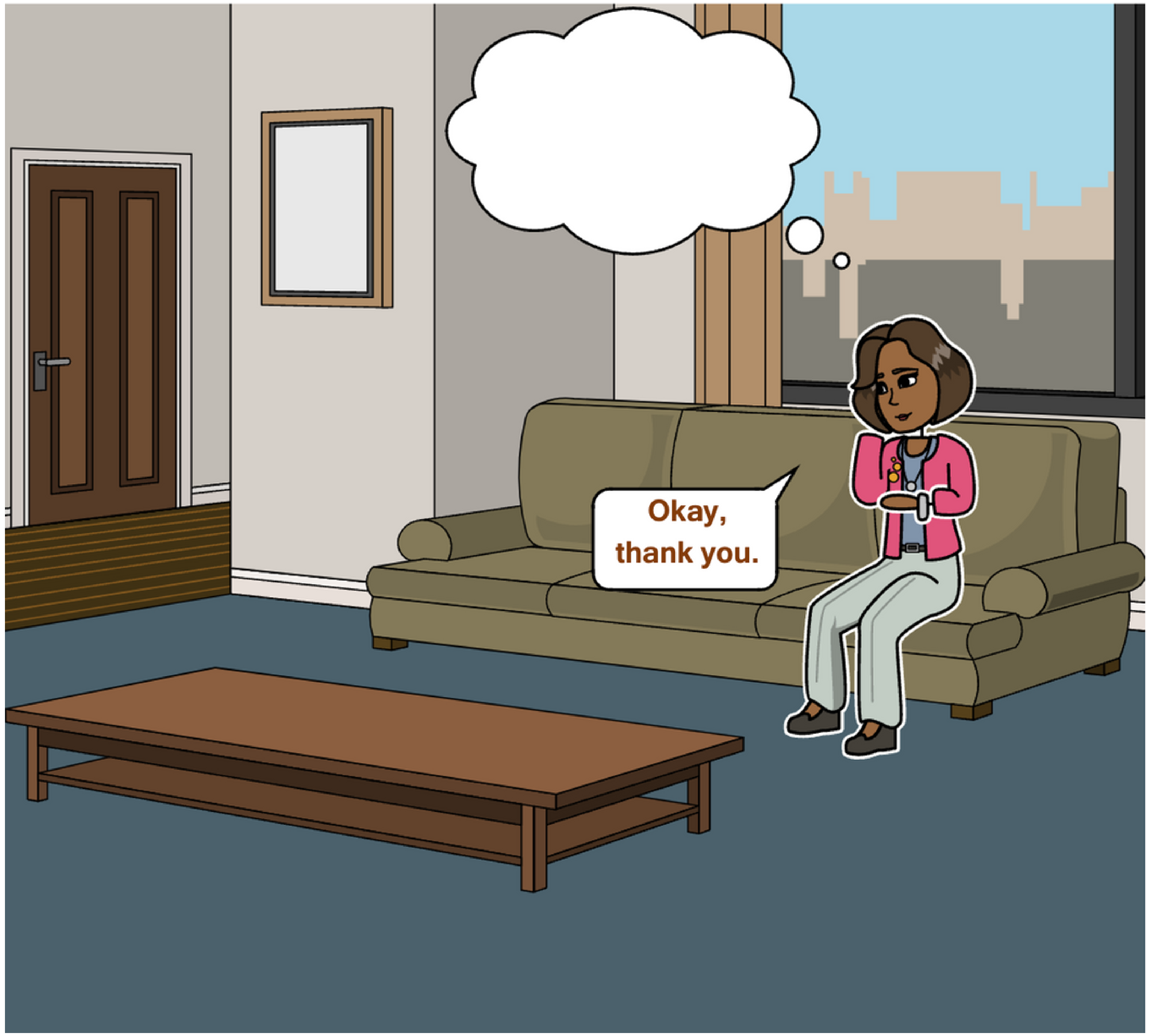
Okay, thank you.

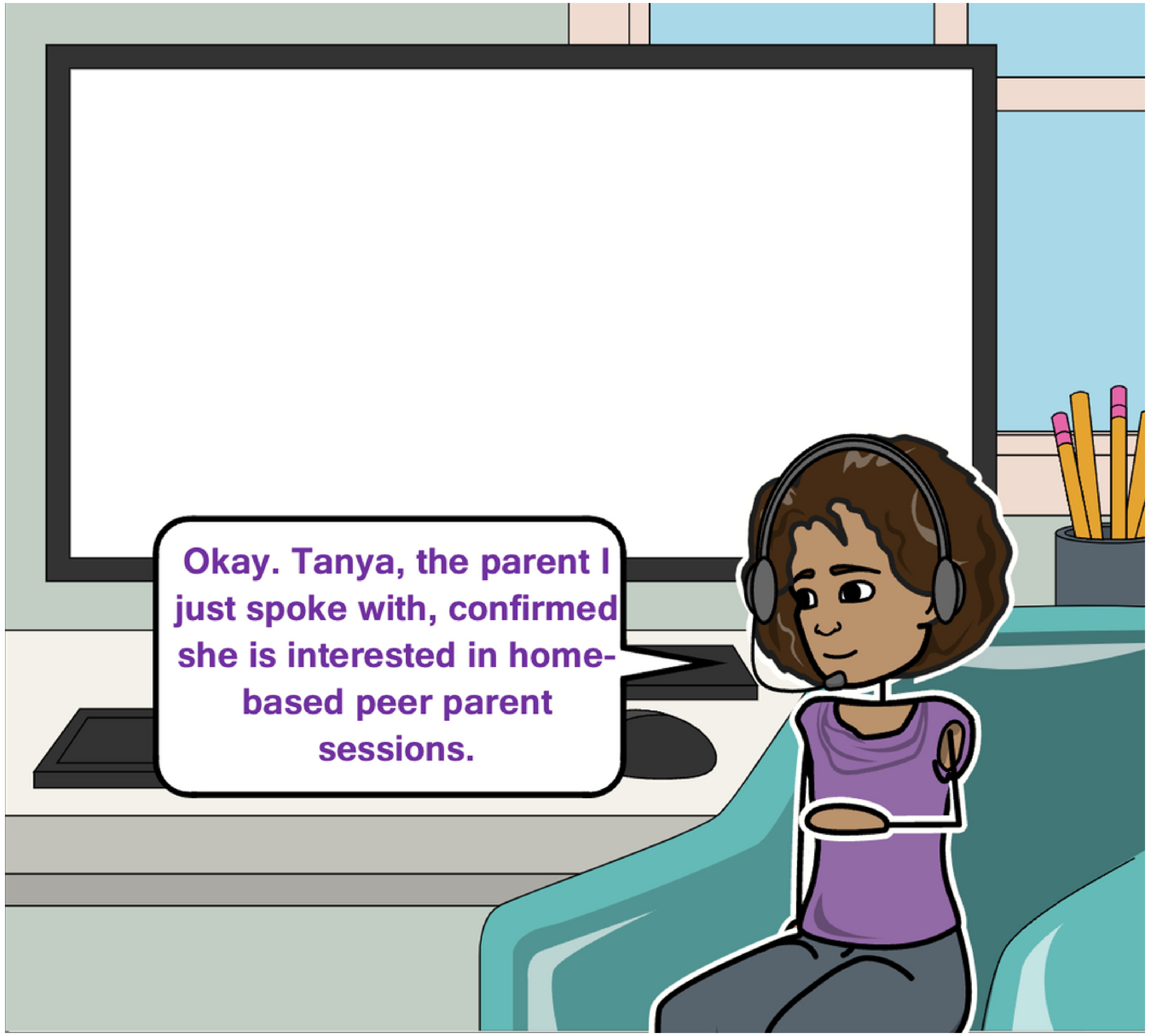
Okay. Tanya, the parent I just spoke with, confirmed she is interested in home-based peer parent sessions.

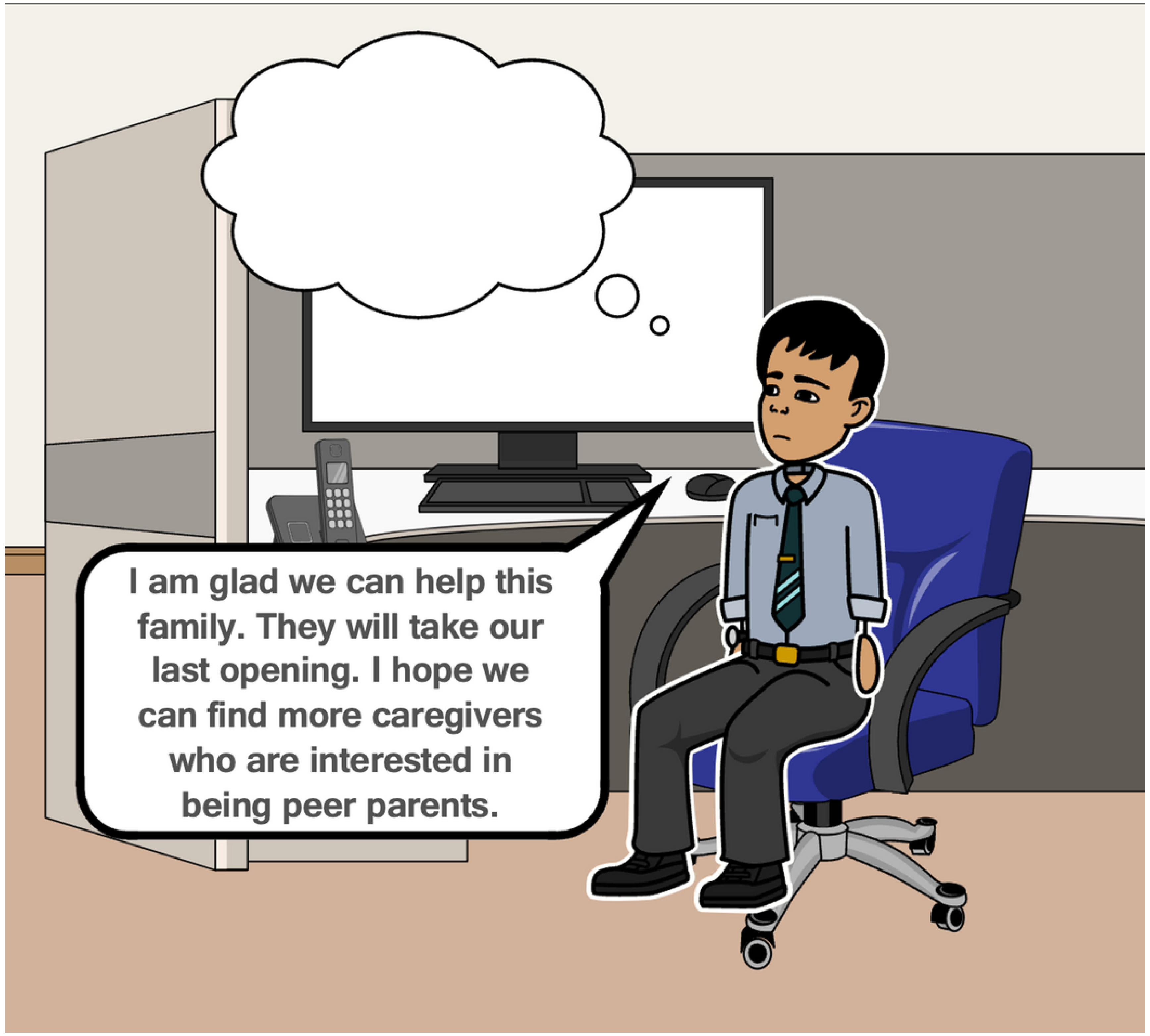
I am glad we can help this family. They will take our last opening. I hope we can find more caregivers who are interested in being peer parents.

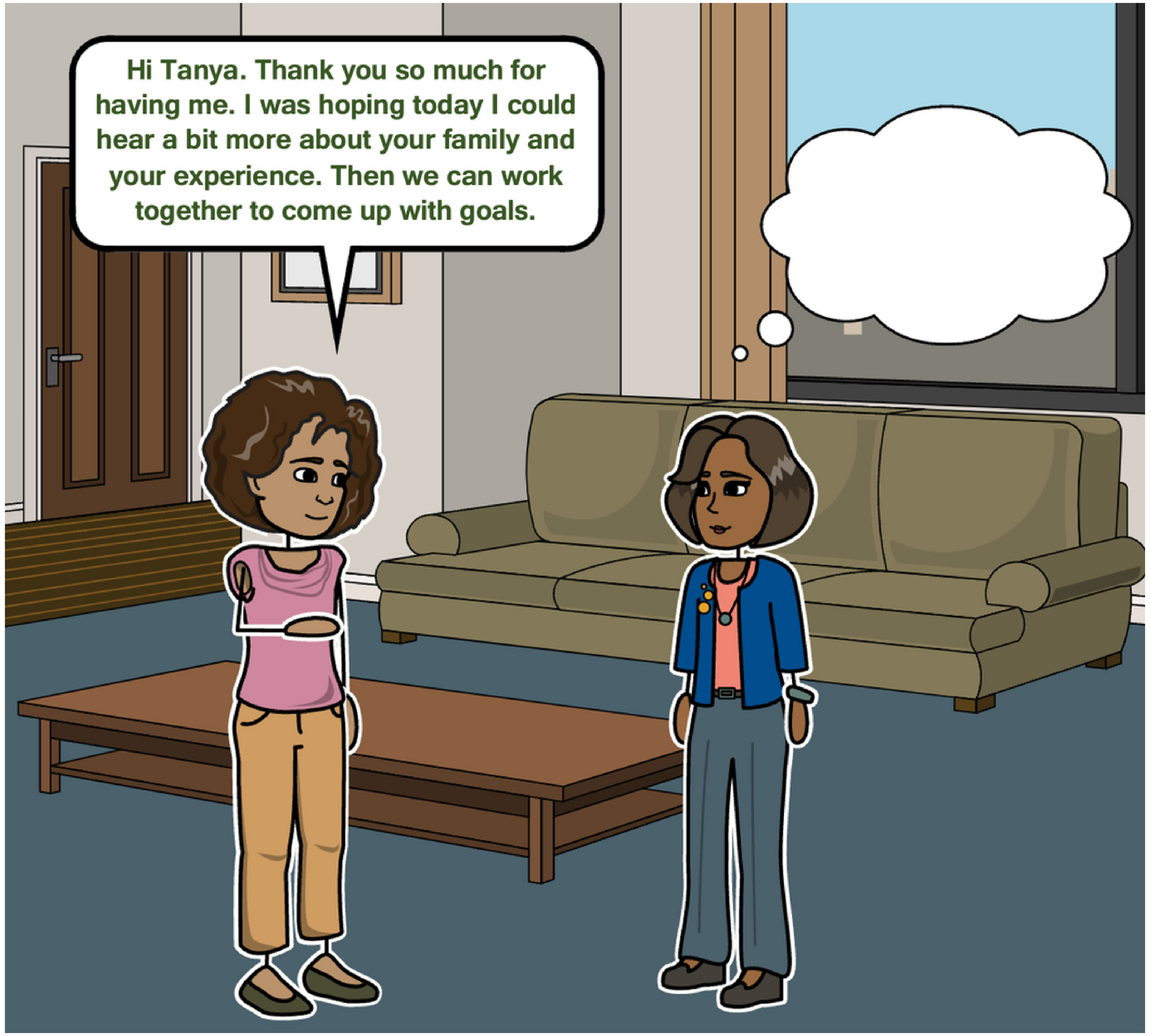
Hi Tanya. Thank you so much for having me. I was hoping today I could hear a bit more about your family and your experience. Then we can work together to come up with goals.

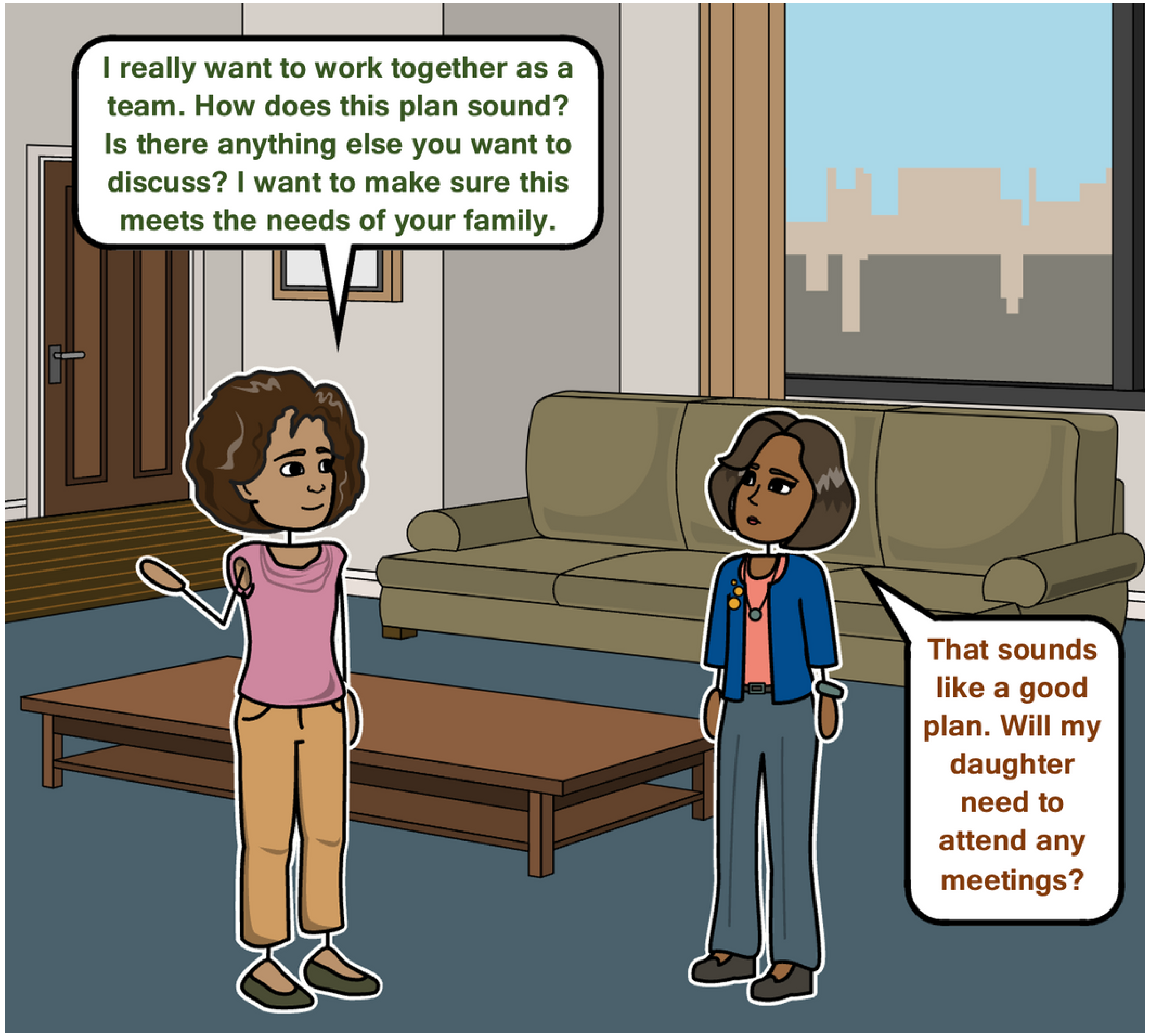
I really want to work together as a team. How does this plan sound? Is there anything else you want to discuss? I want to make sure this meets the needs of your family.
That sounds like a good plan. Will my daughter need to attend any meetings?

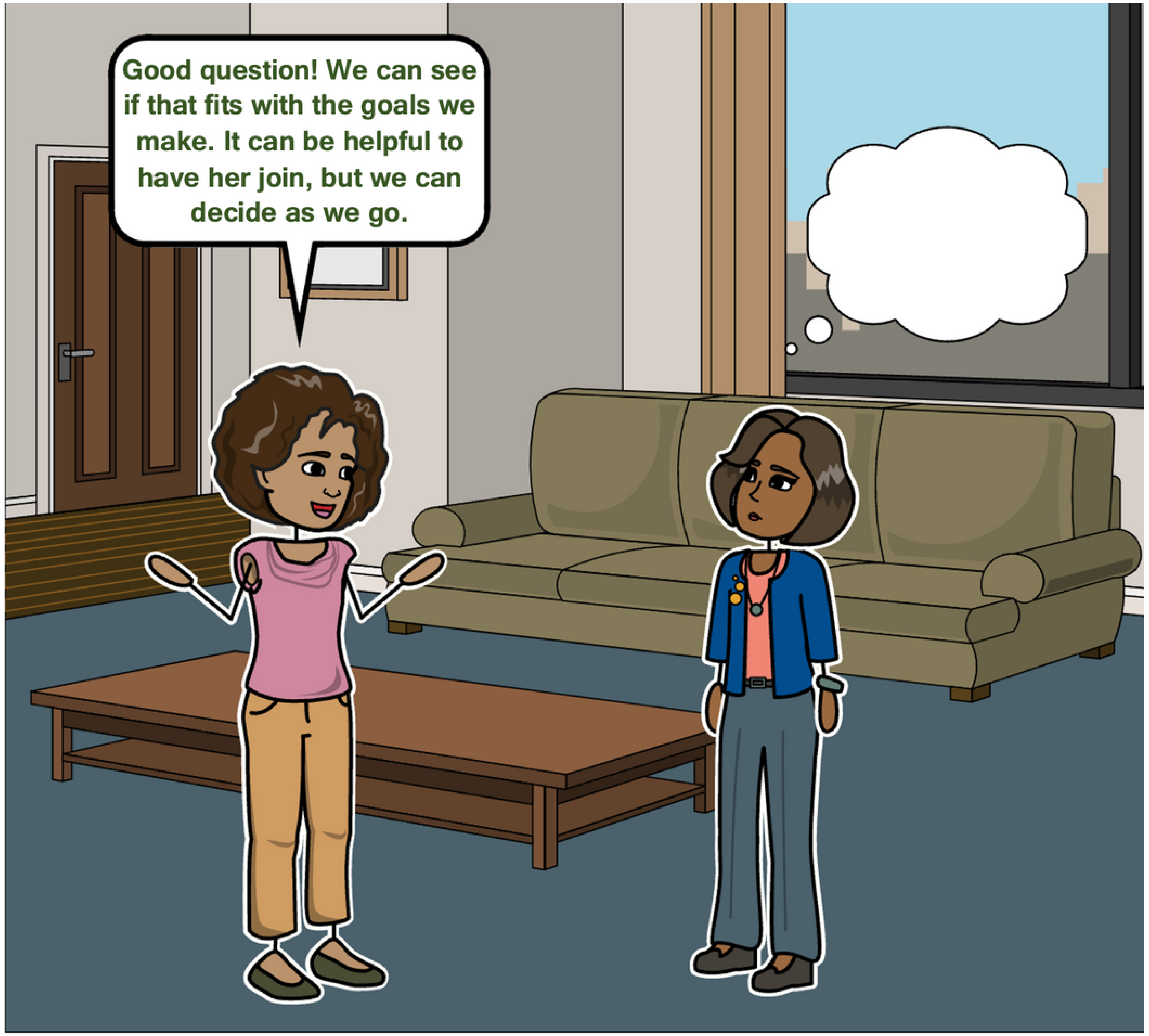
Good question! We can see if that fits with the goals we make. It can be helpful to have her join, but we can decide as we go.

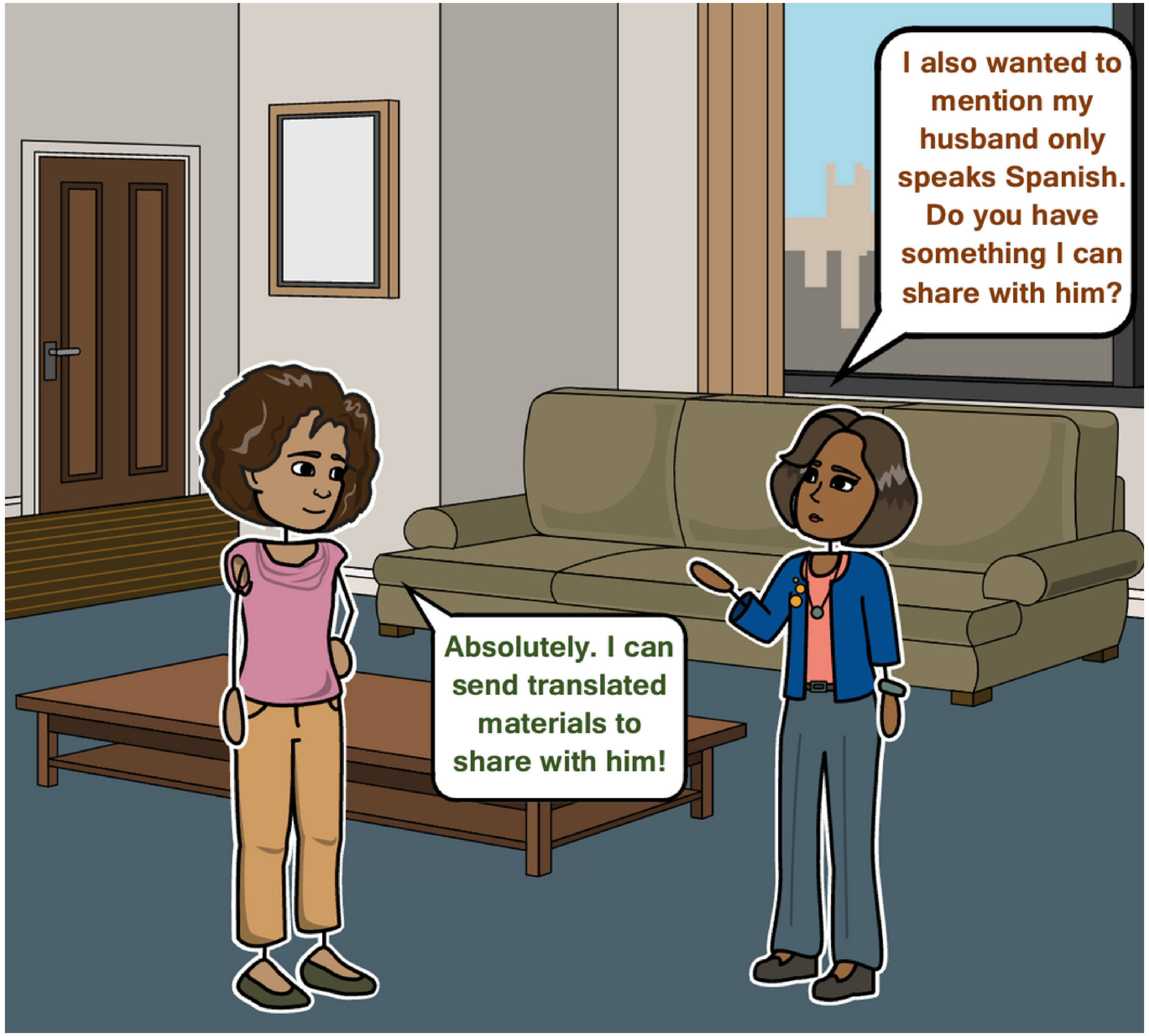
I also wanted to mention my husband only speaks Spanish. Do you have something I can share with him?
Absolutely. I can send translated materials to share with him!

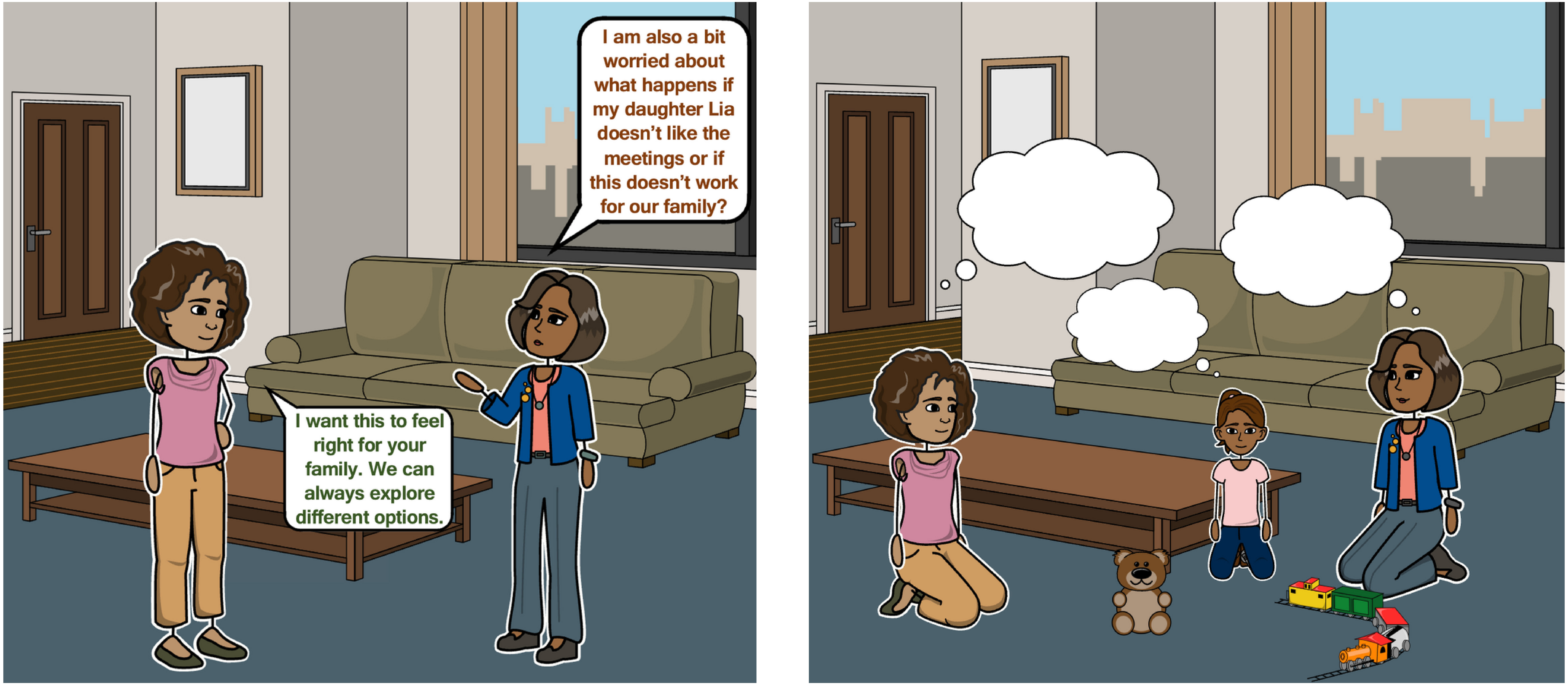
I am also a bit worried about what happens if my daughter Lia doesn't like the meetings or if this doesn't work for our family?
I want this to feel right for your family. We can always explore different options.

# Community Health Worker Model

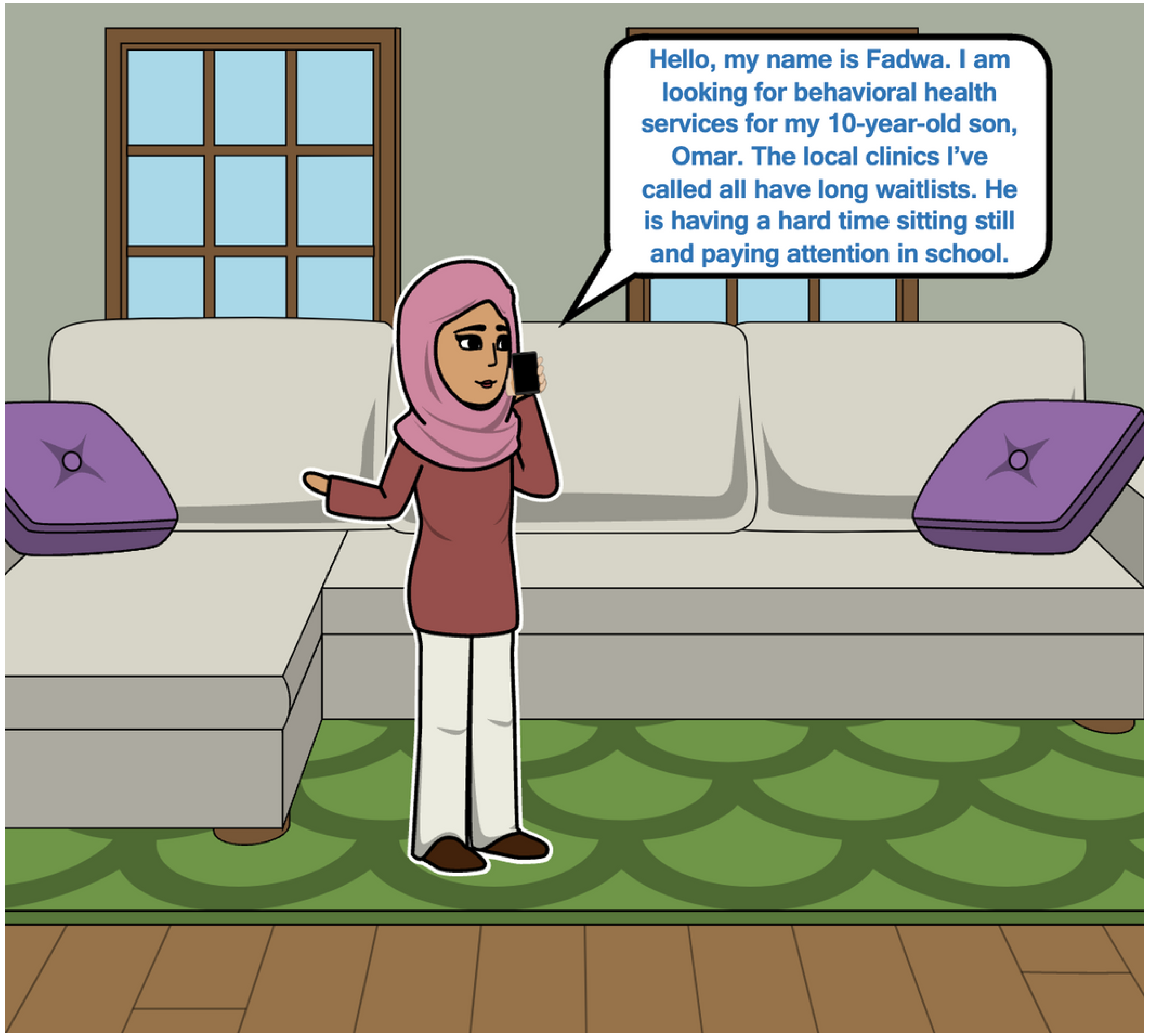


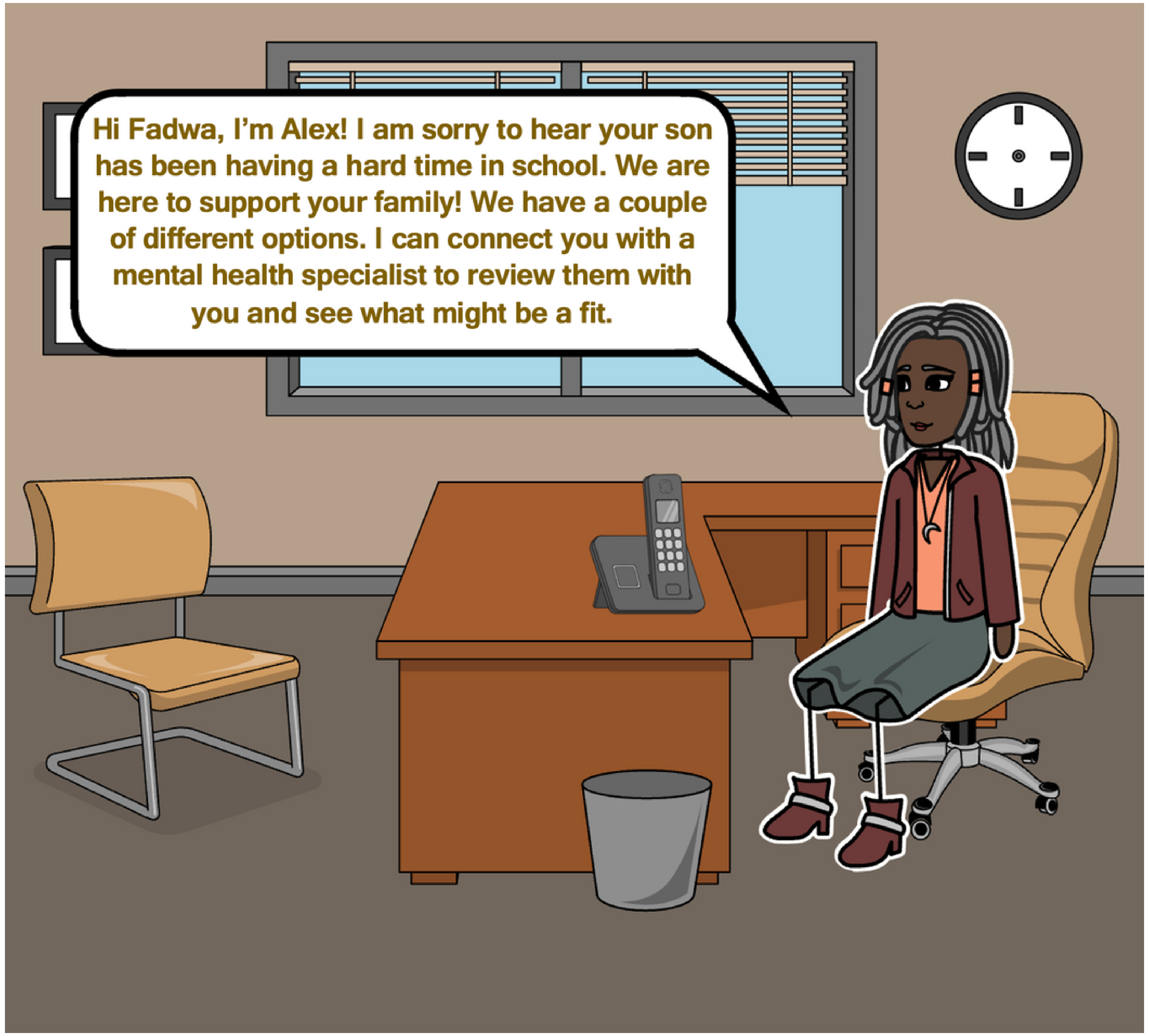

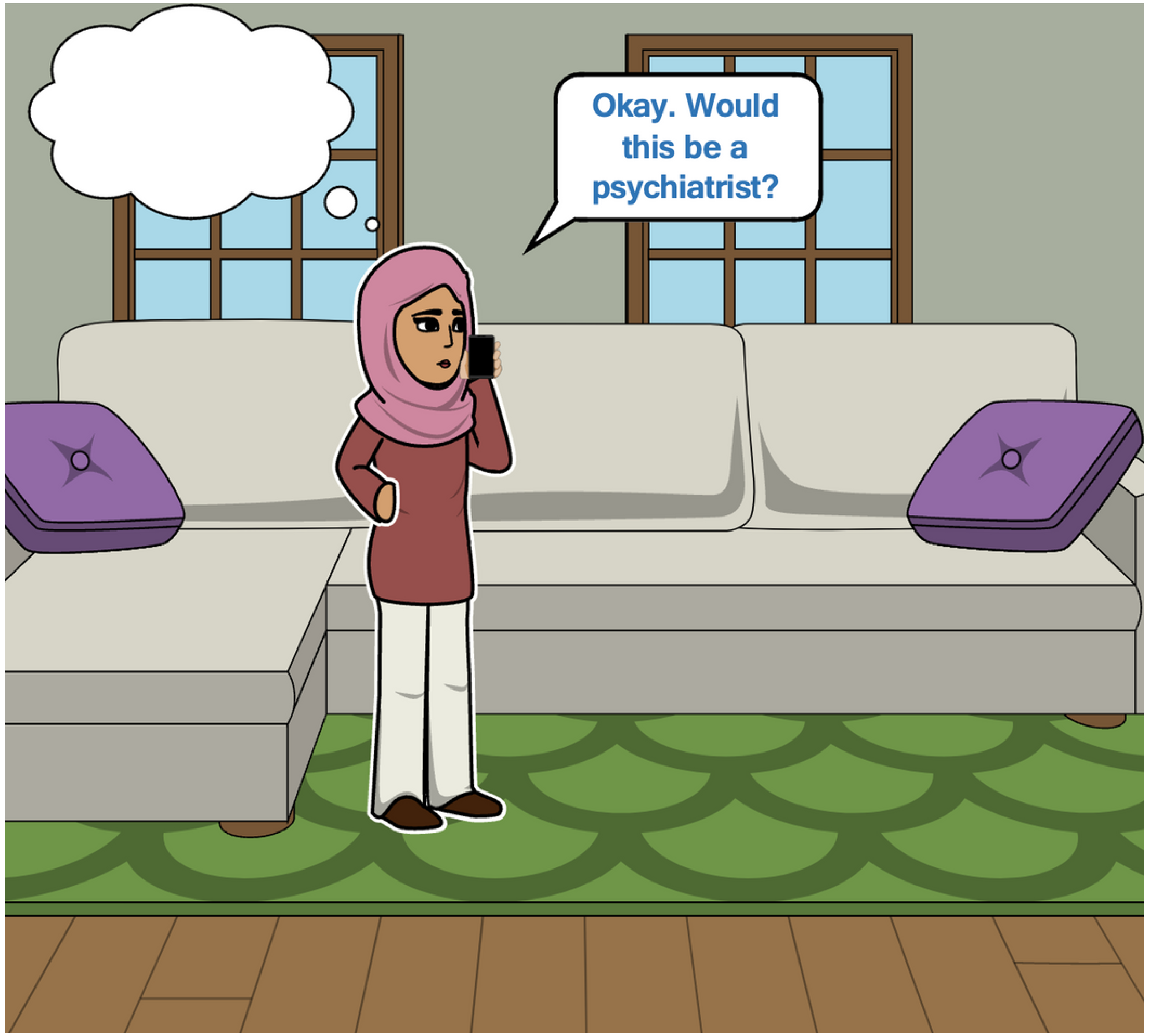
Okay. Would this be a psychiatrist?

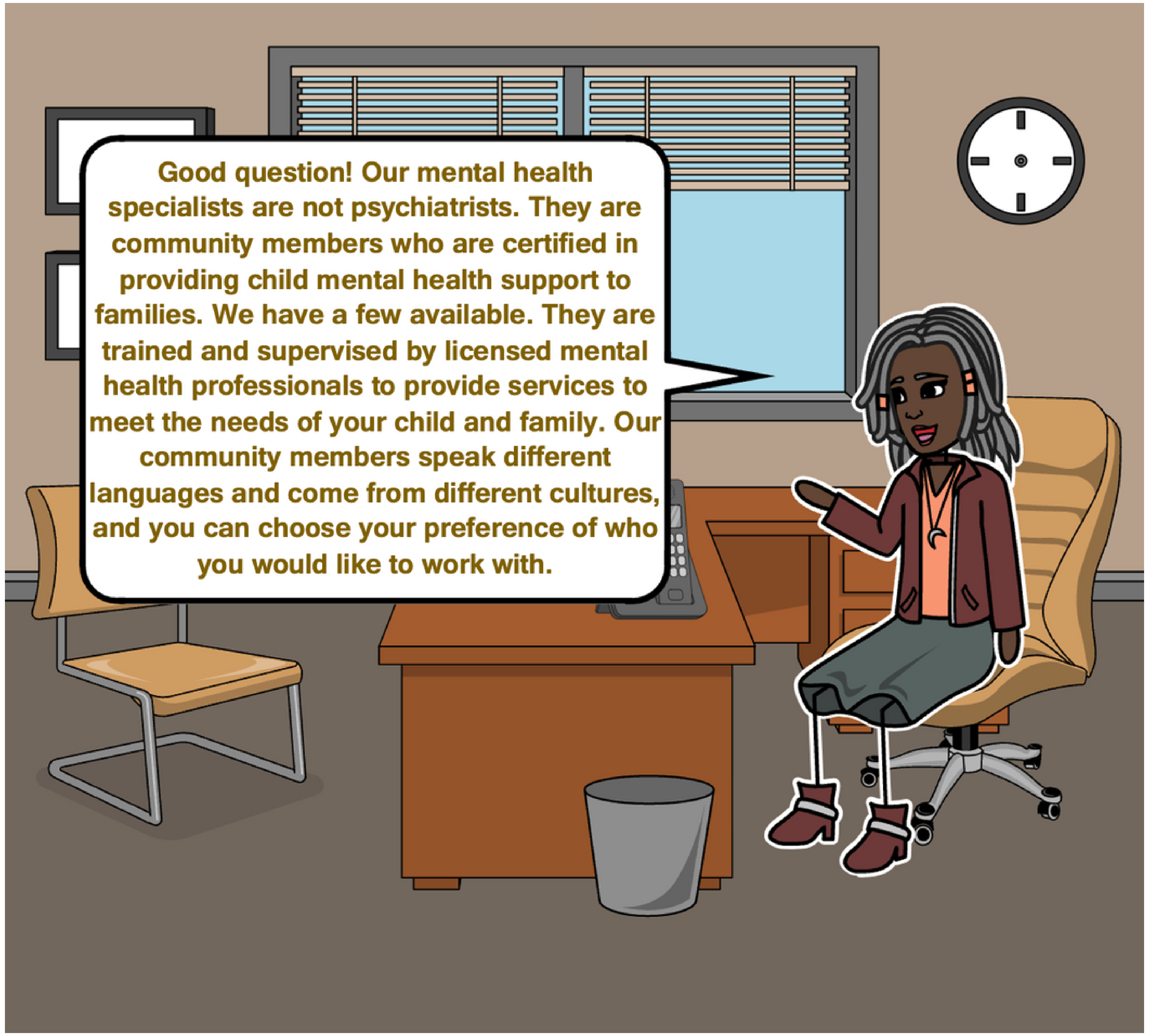
Good question! Our mental health specialists are not psychiatrists. They are community members who are certified in providing child mental health support to families. We have a few available. They are trained and supervised by licensed mental health professionals to provide services to meet the needs of your child and family. Our community members speak different languages and come from different cultures, and you can choose your preference of who you would like to work with.

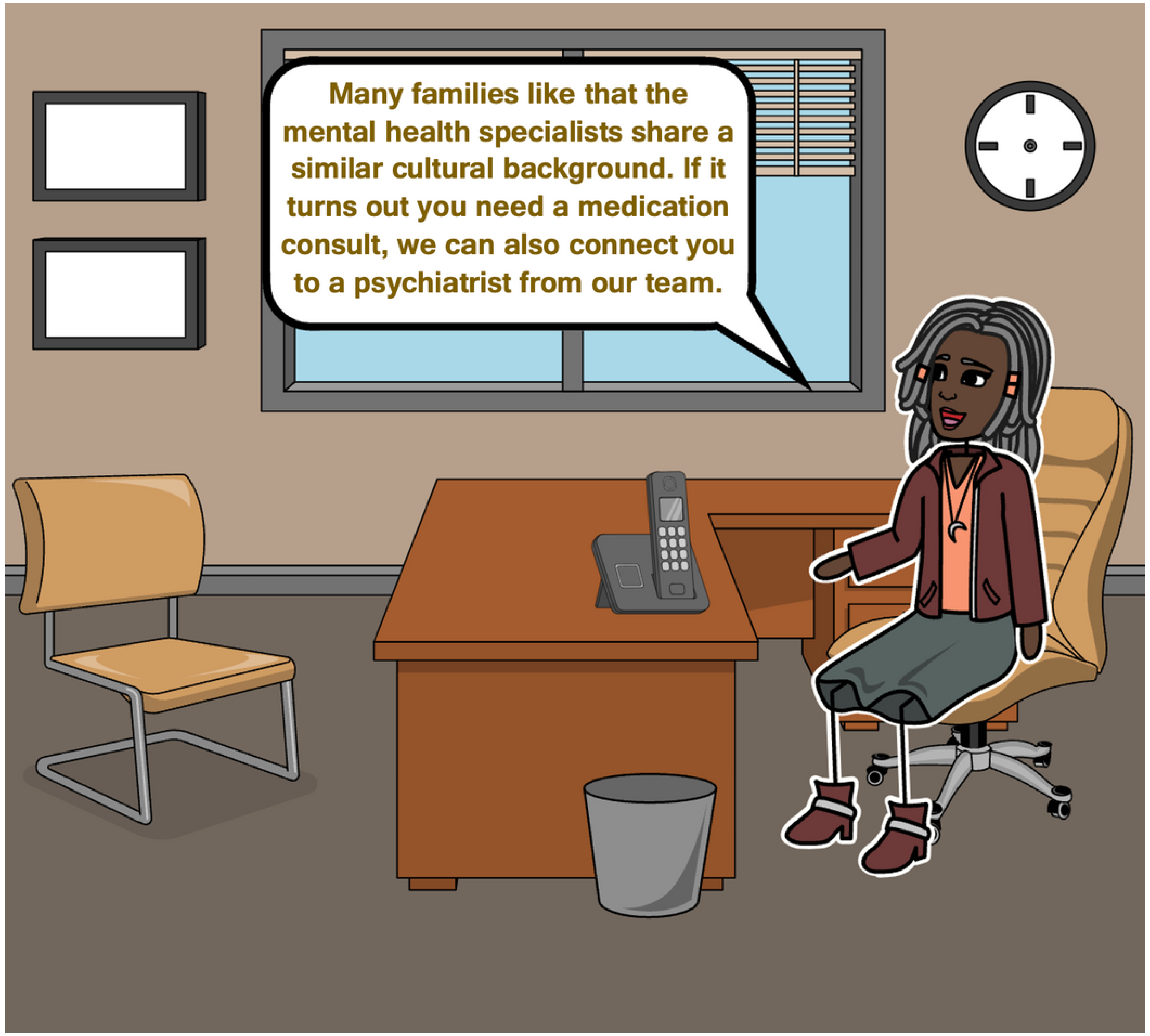
Many families like that the mental health specialists share a similar cultural background. If it turns out you need a medication consult, we can also connect you to a psychiatrist from our team.

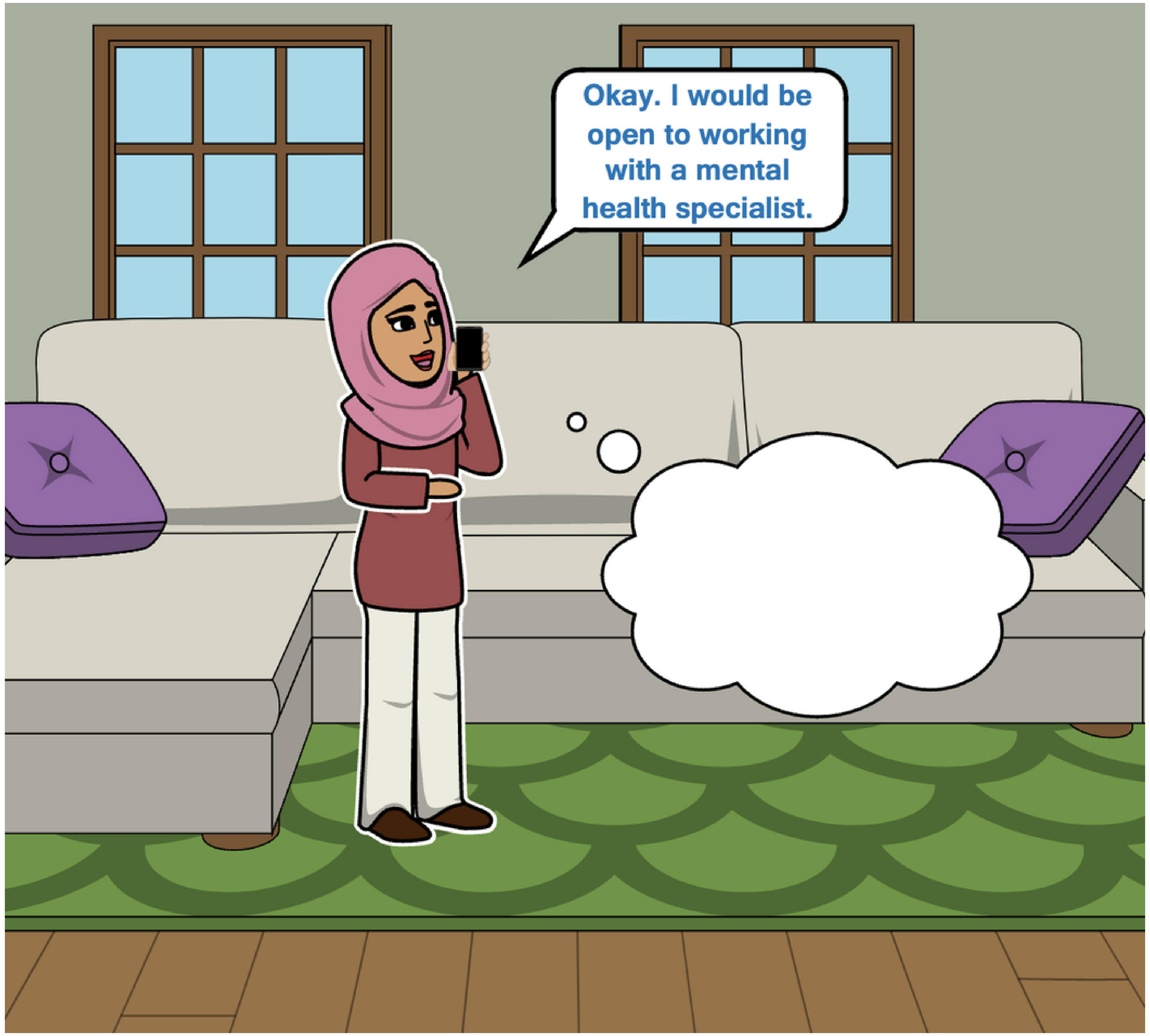
Okay. I would be open to working with a mental health specialist.

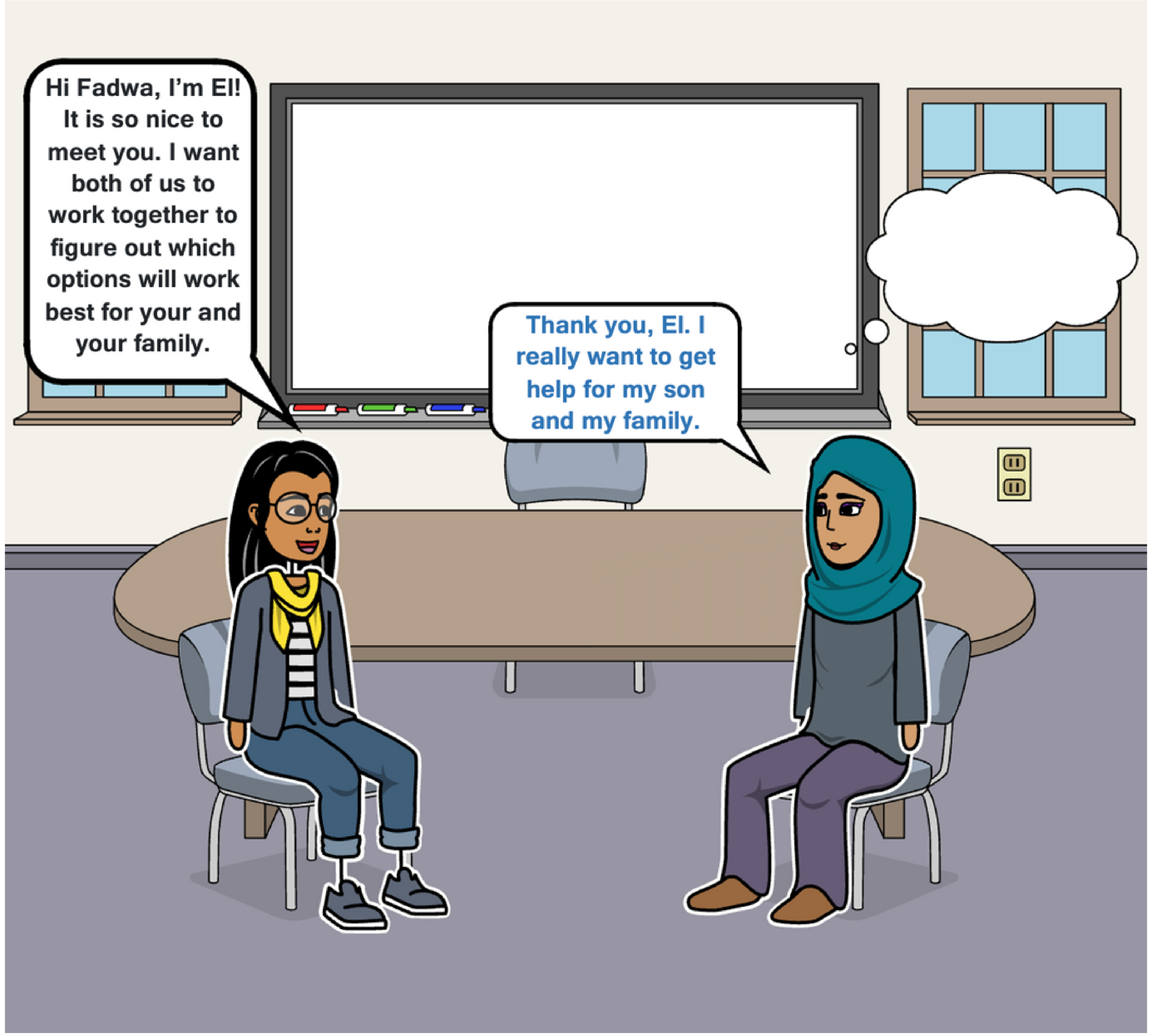
Hi Fadwa, I'm El! It is so nice to meet you. I want both of us to work together to figure out which options will work best for your and your family.
Thank you, El. I really want to get help for my son and my family.

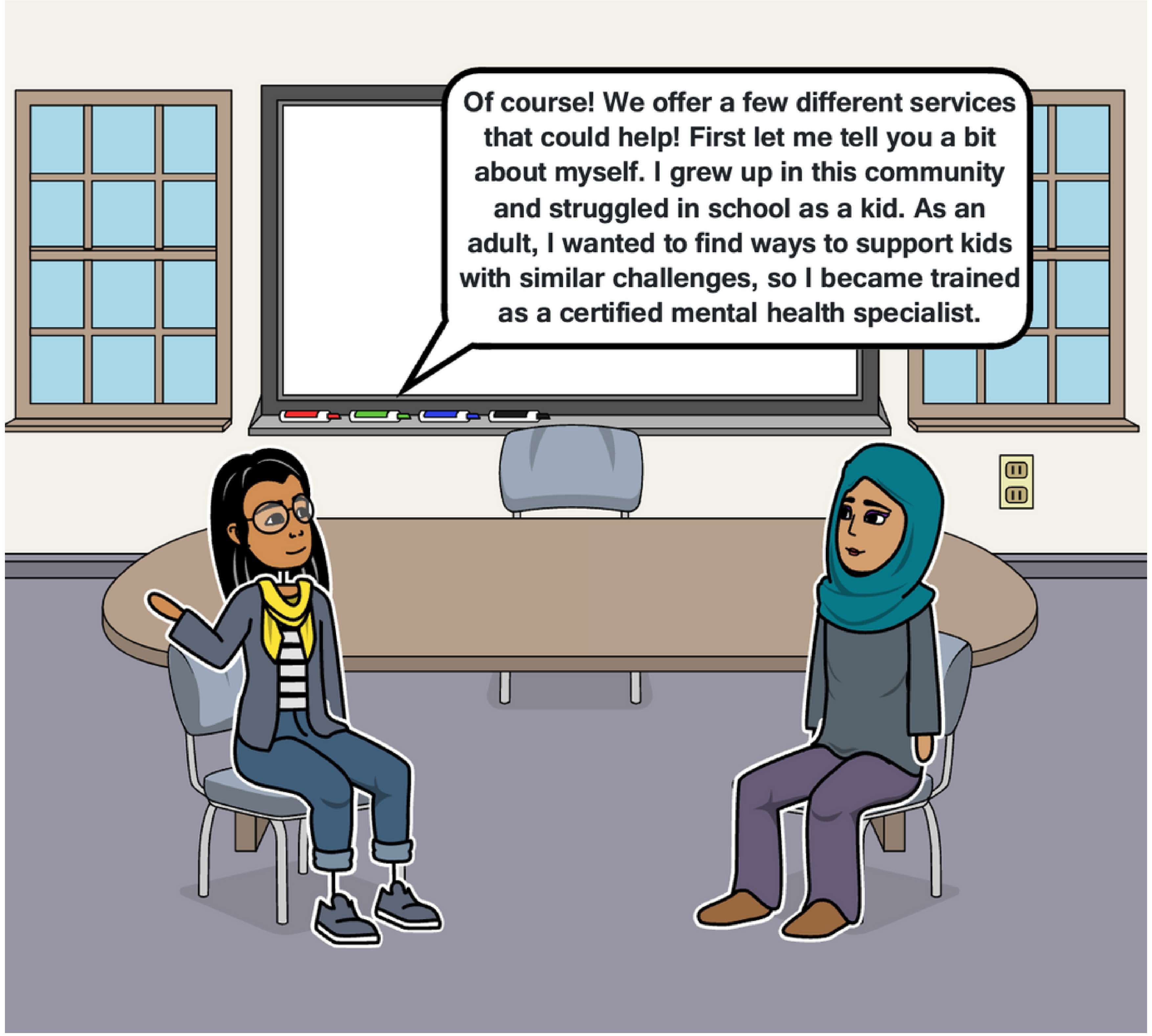
Of course! We offer a few different services that could help! First let me tell you a bit about myself. I grew up in this community and struggled in school as a kid. As an adult, I wanted to find ways to support kids with similar challenges, so I became trained as a certified mental health specialist.

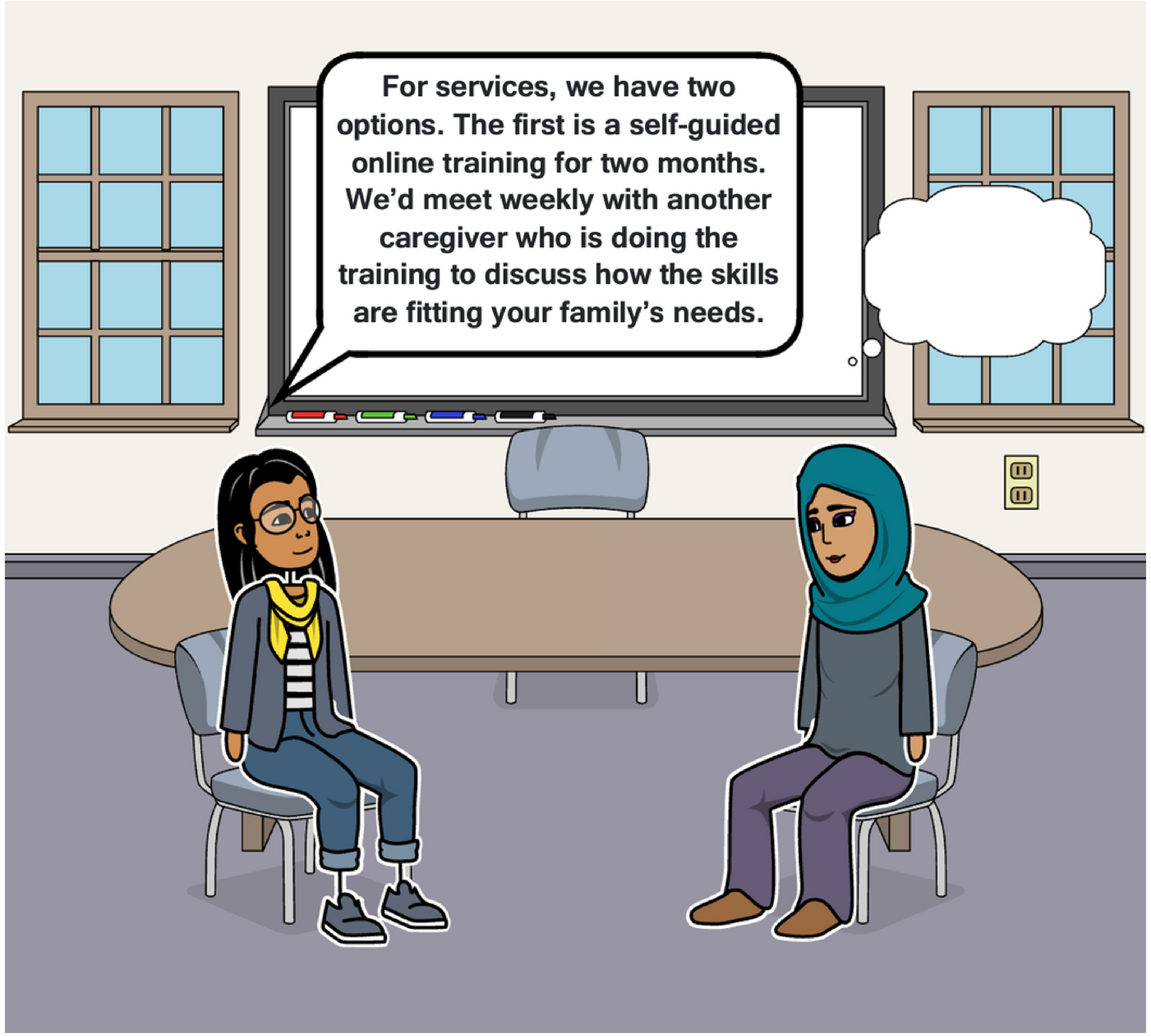
For services, we have two options. The first is a self-guided online training for two months. We'd meet weekly with another caregiver who is doing the training to discuss how the skills are fitting your family's needs.

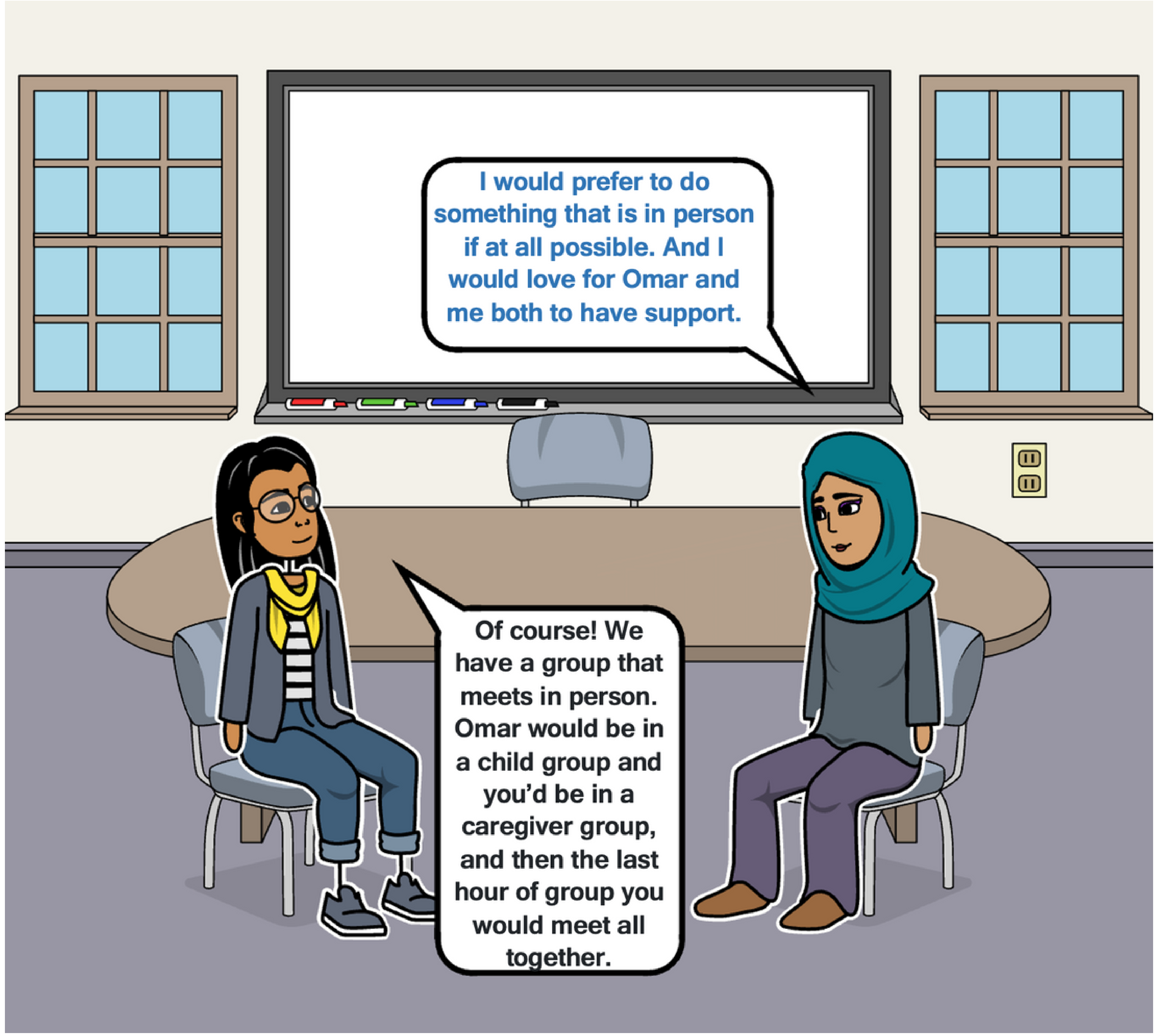
I would prefer to do something that is in person if at all possible. And I would love for Omar and me both to have support.
Of course! We have a group that meets in person. Omar would be in a child group and you'd be in a caregiver group, and then the last hour of group you would meet all together.

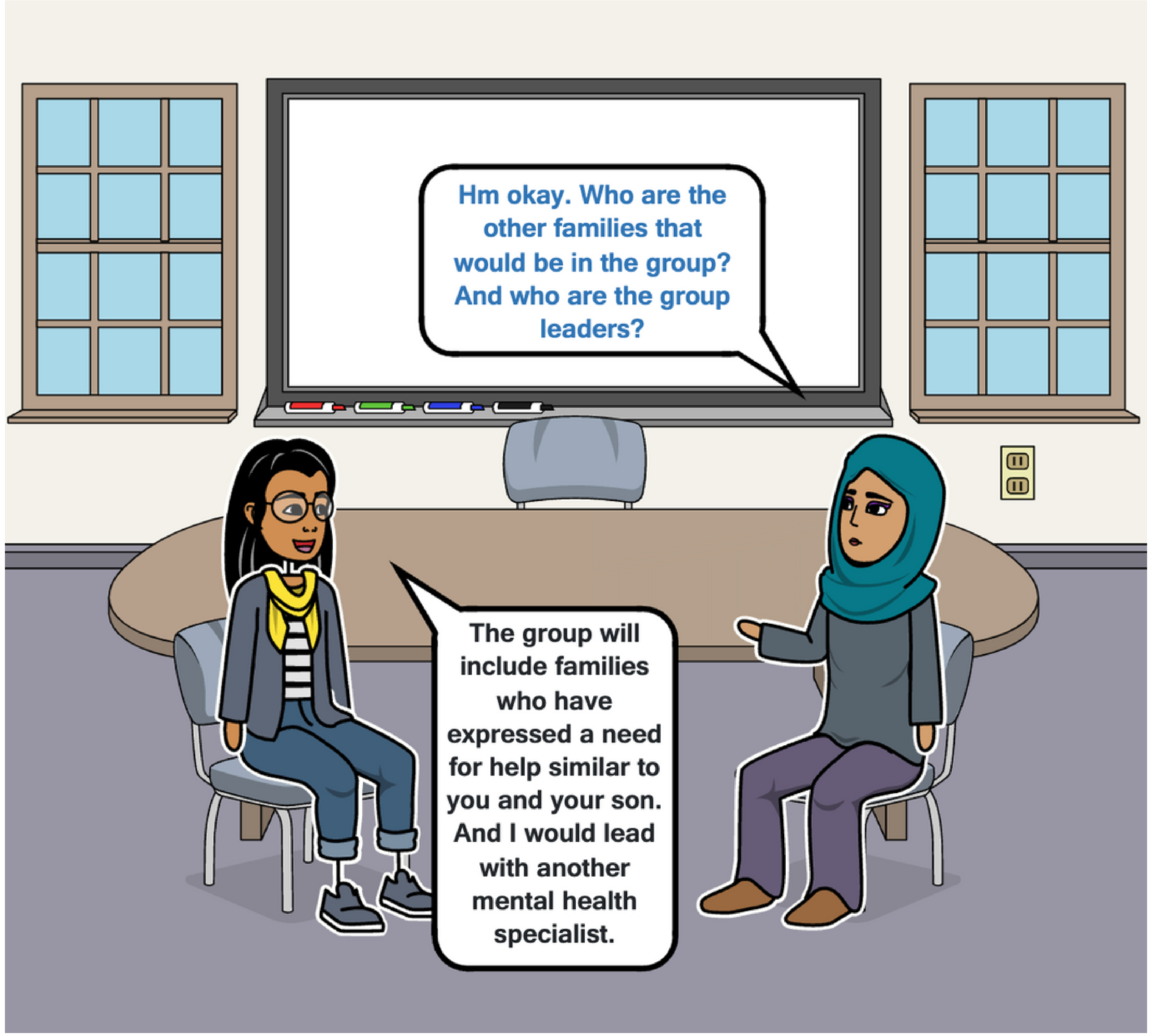
Hm okay. Who are the other families that would be in the group? And who are the group leaders?
The group will include families who have expressed a need for help similar to you and your son. And I would lead with another mental health specialist.

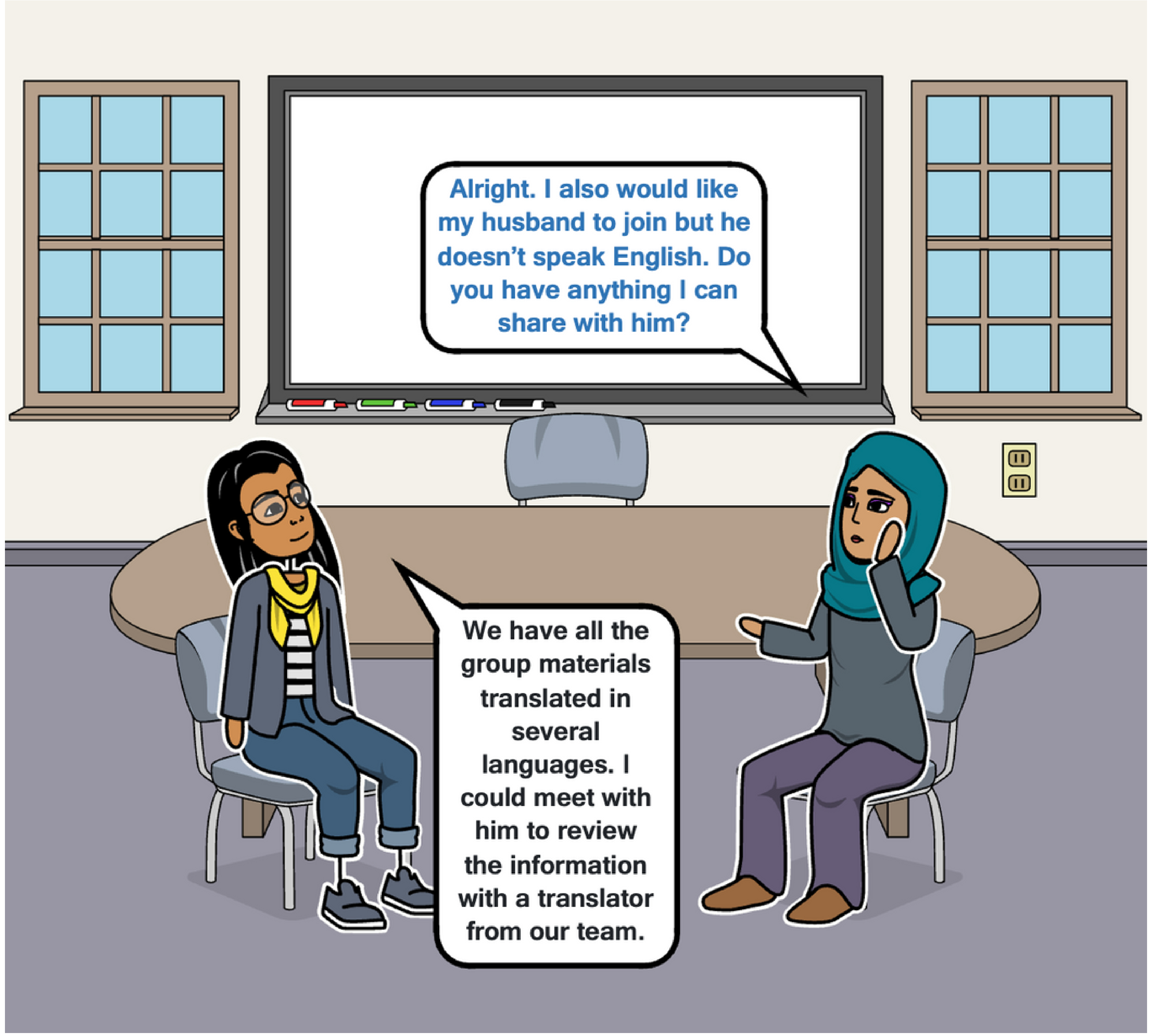
Alright. I also would like my husband to join but he doesn't speak English. Do you have anything I can share with him?
We have all the group materials translated in several languages. I could meet with him to review the information with a translator from our team.

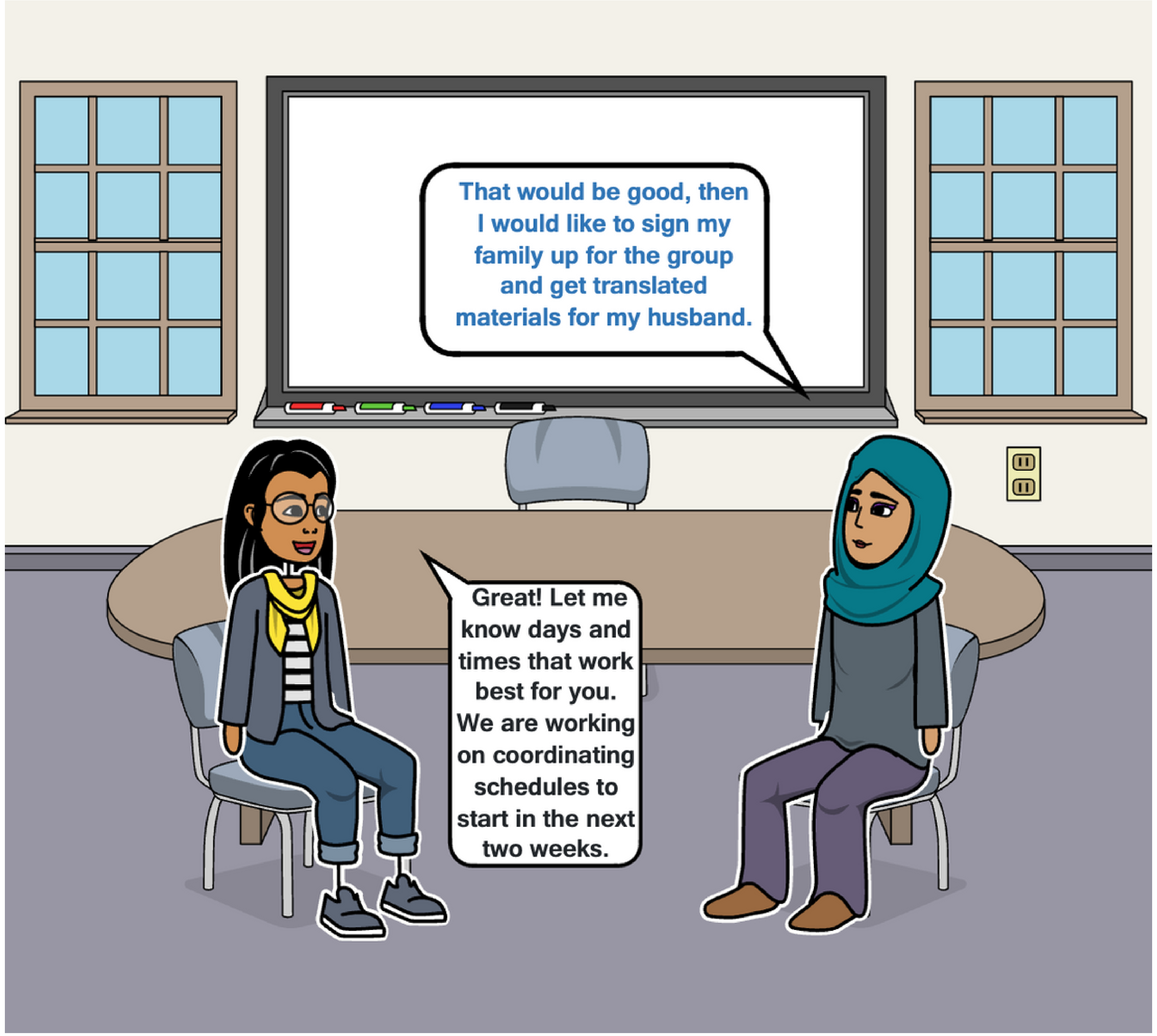
That would be good, then I would like to sign my family up for the group and get translated materials for my husband.
Great! Let me know days and times that work best for you. We are working on coordinating schedules to start in the next two weeks.

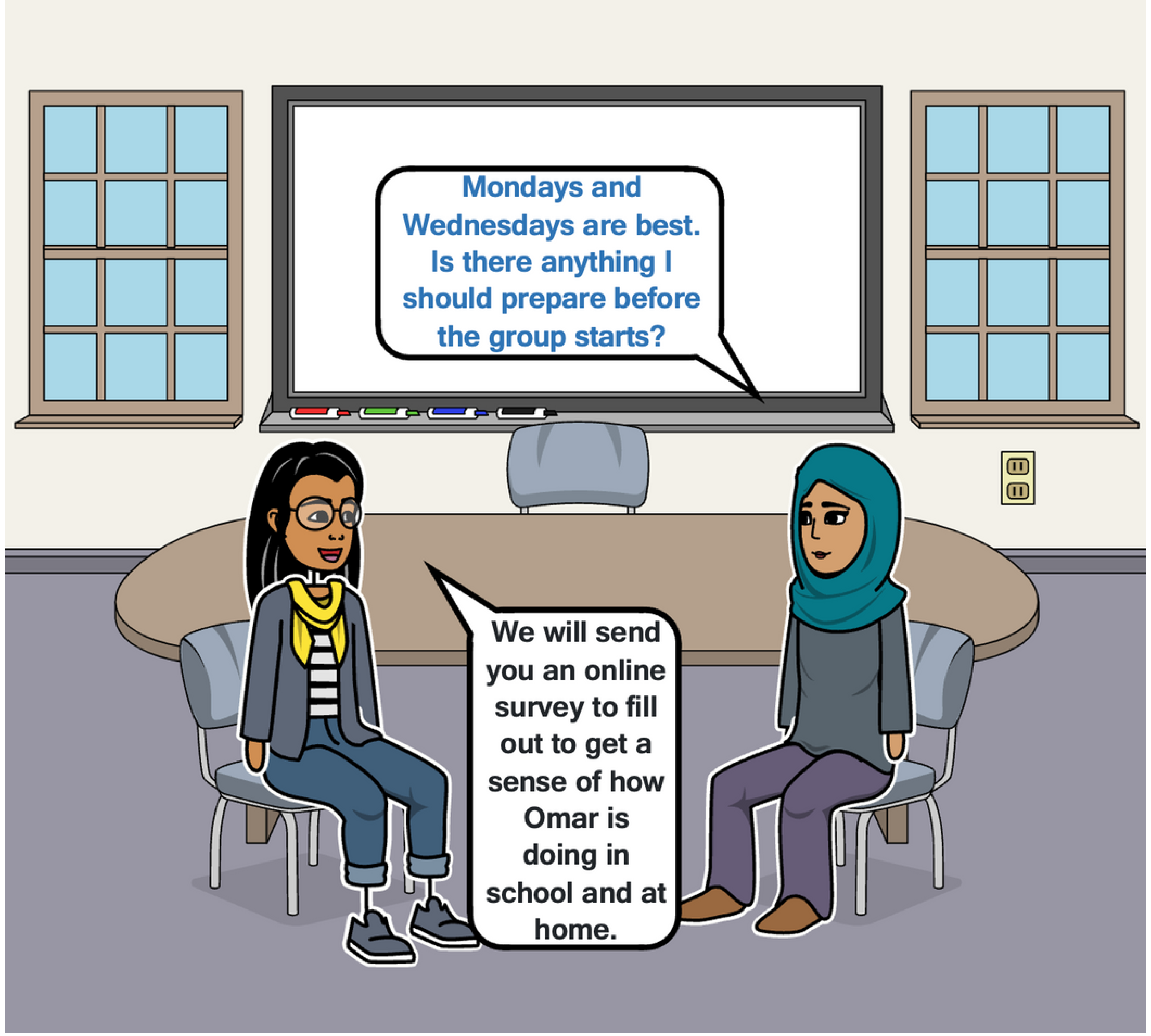
Mondays and Wednesdays are best. Is there anything I should prepare before the group starts?
We will send you an online survey to fill out to get a sense of how Omar is doing in school and at home.

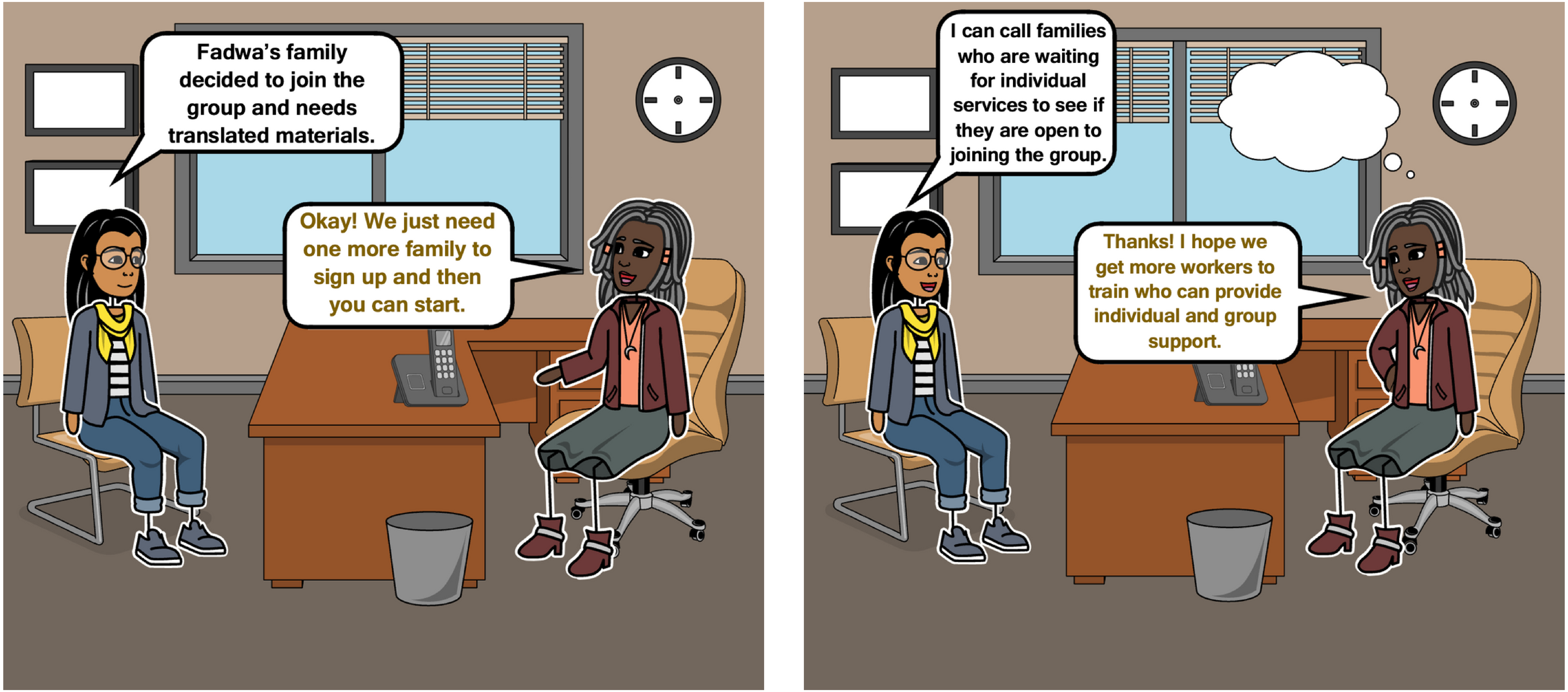
Fadwa's family decided to join the group and needs translated materials.
Okay! We just need one more family to sign up and then you can start.
I can call families who are waiting for individual services to see if they are open to joining the group.
Thanks! I hope we get more workers to train who can provide individual and group support.

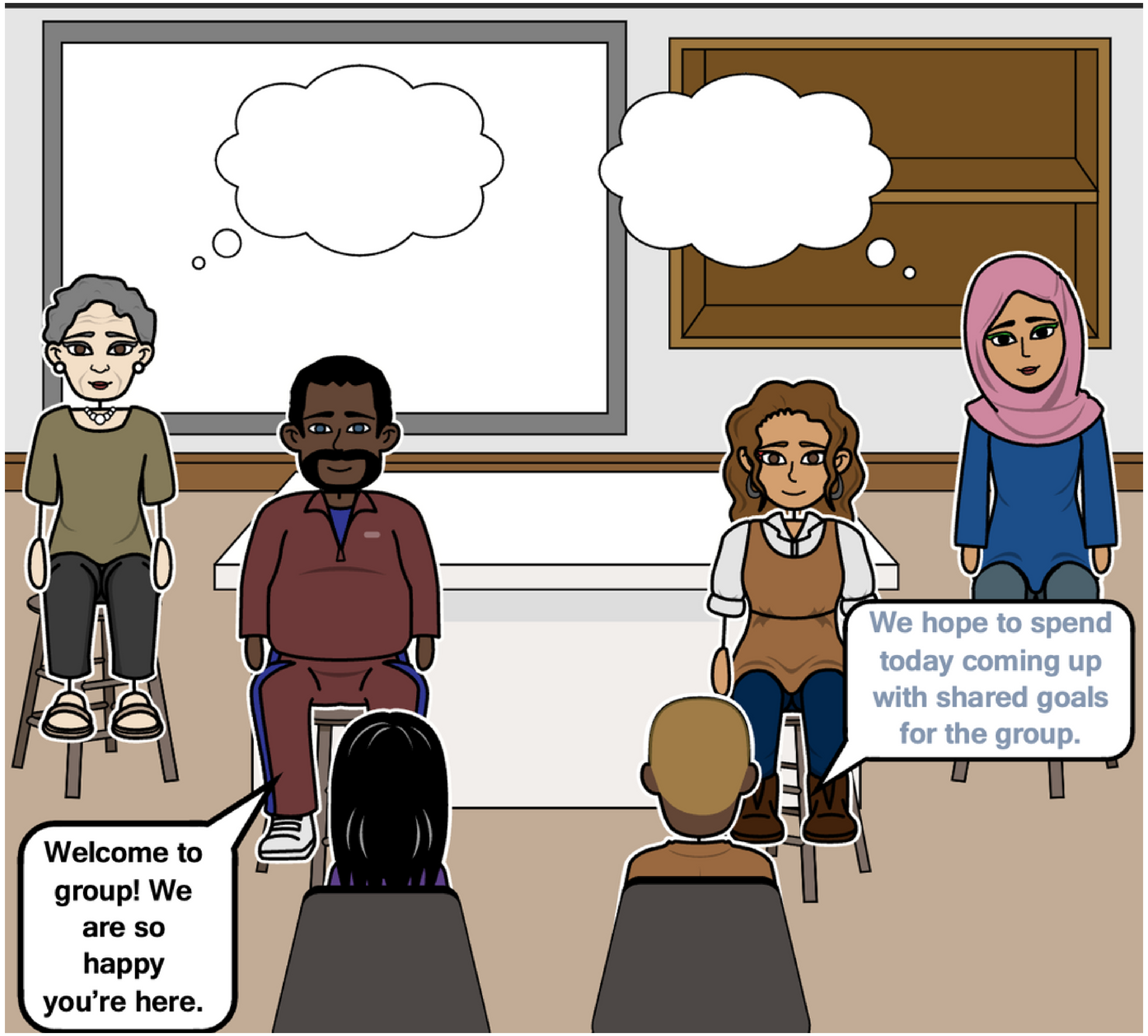
We hope to spend today coming up with shared goals for the group.
Welcome to group! We are so happy you're here.

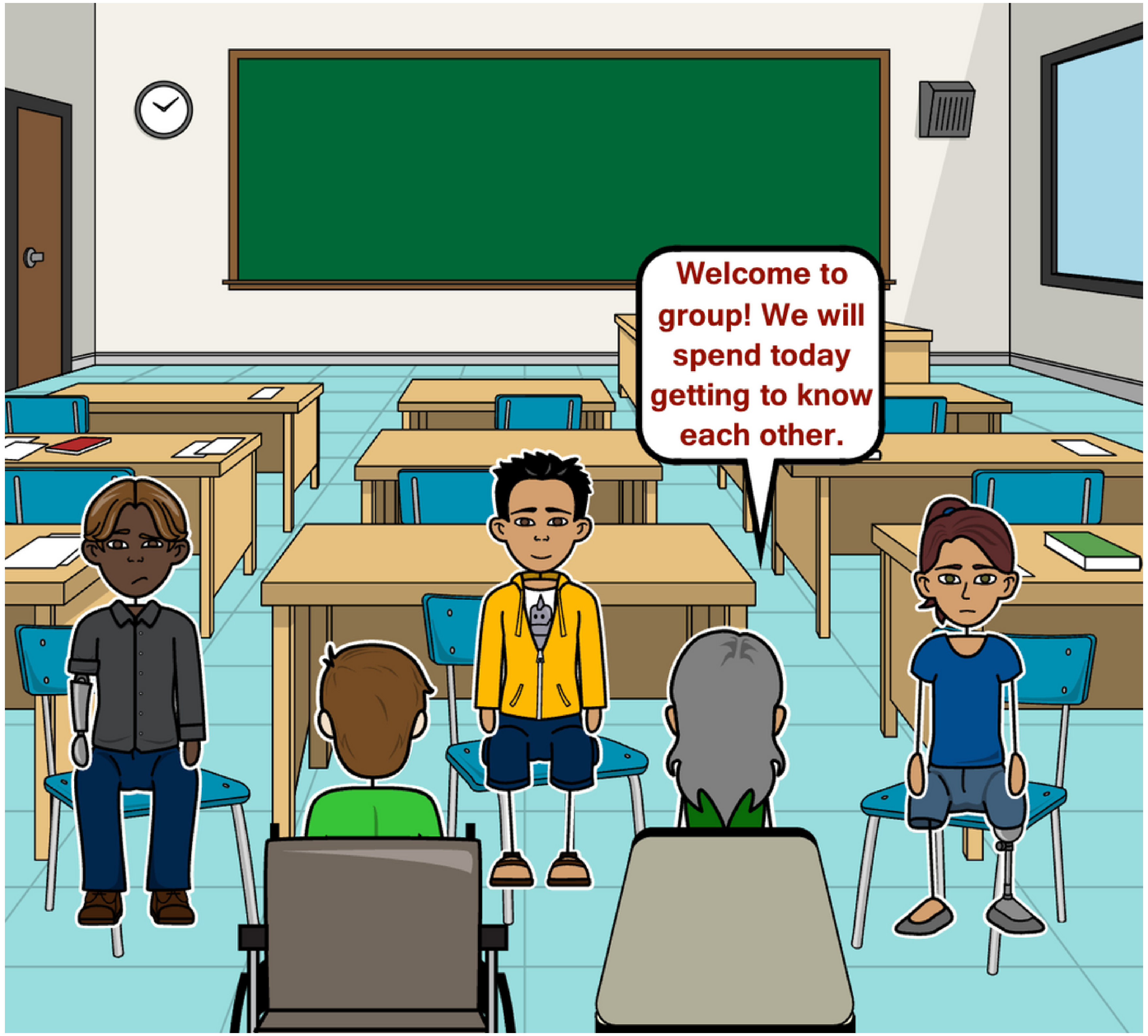
Welcome to group! We will spend today getting to know each other.

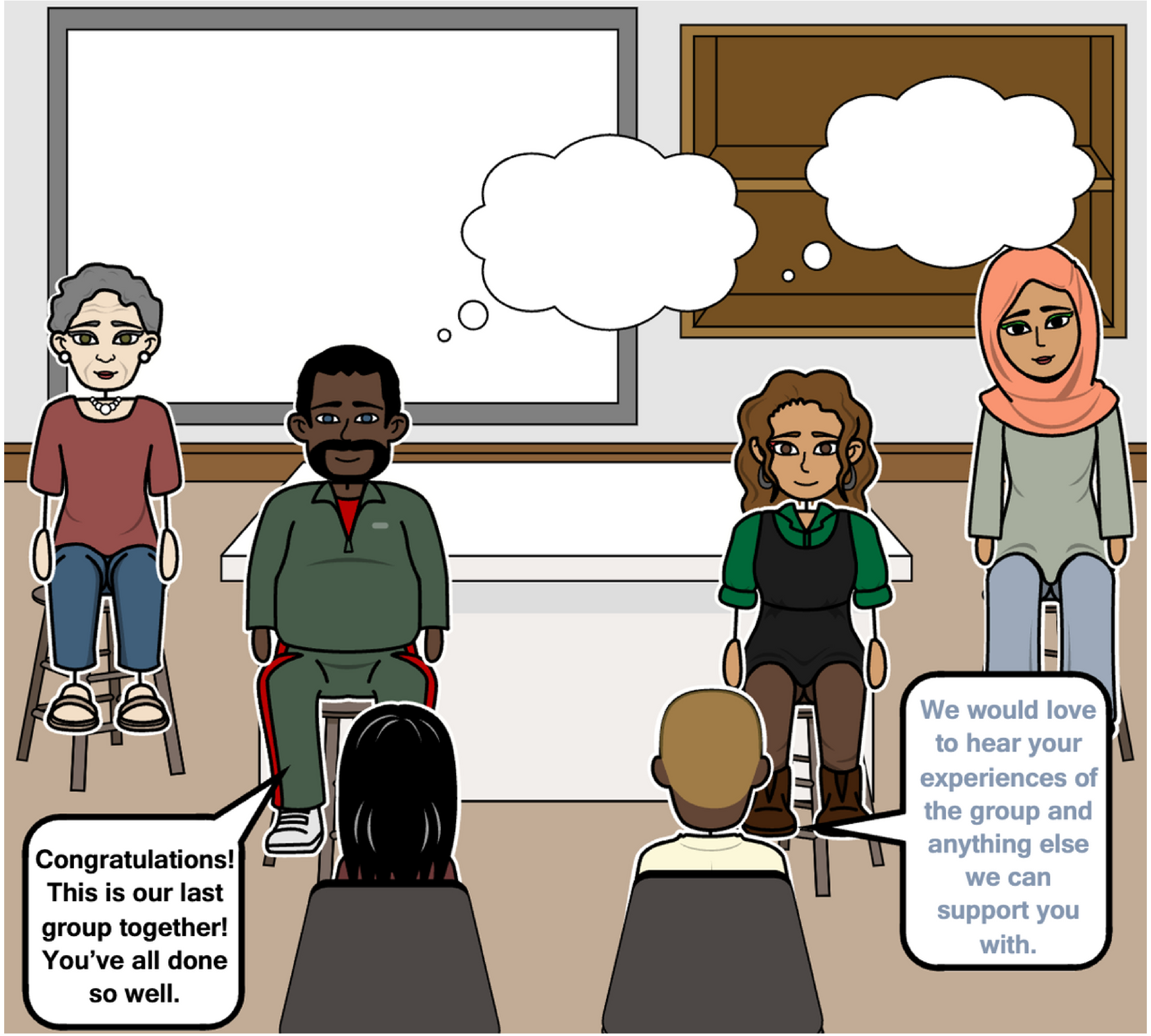
Congratulations! This is our last group together! You've all done so well.
We would love to hear your experiences of the group and anything else we can support you with.

# Caregiver-Youth Combined Group

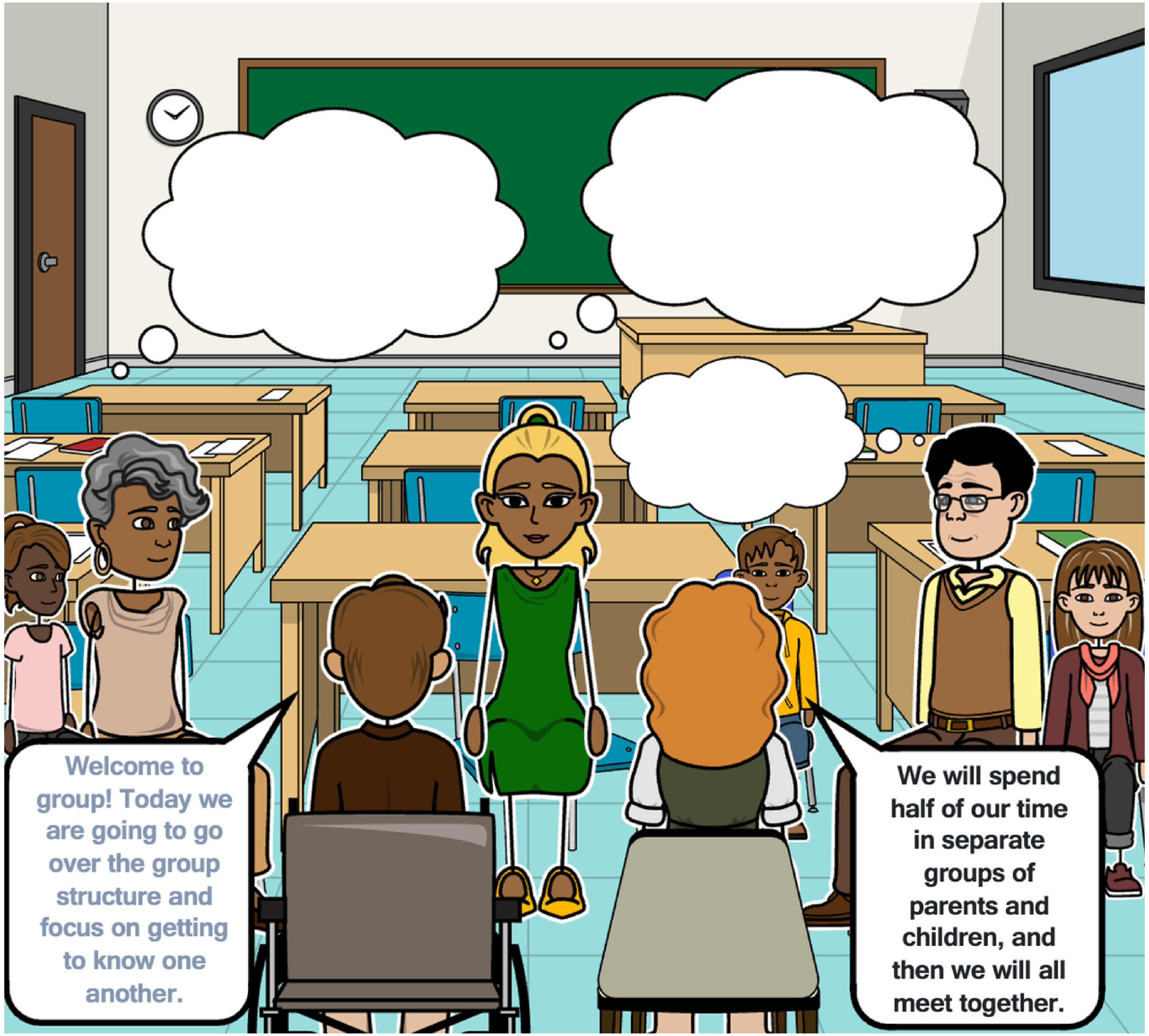

# Youth Worker Model

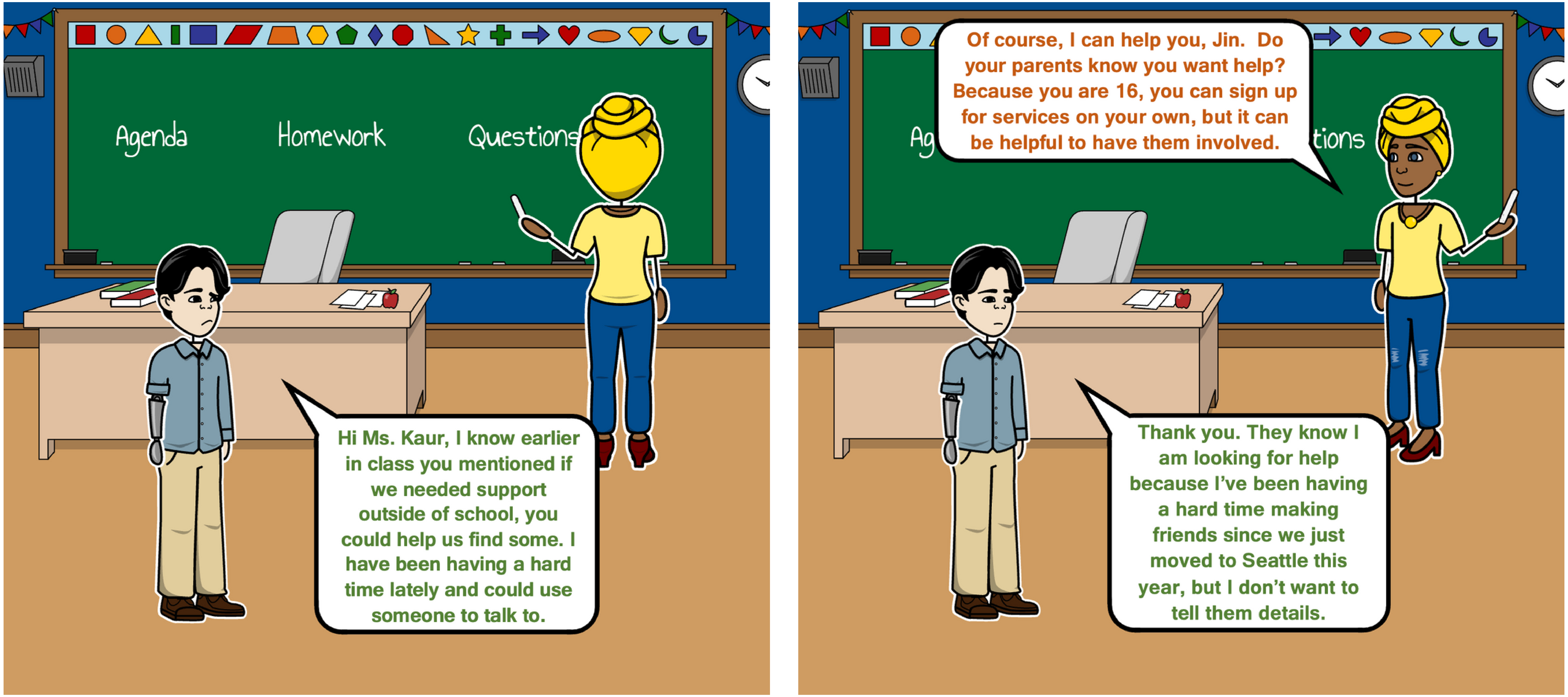

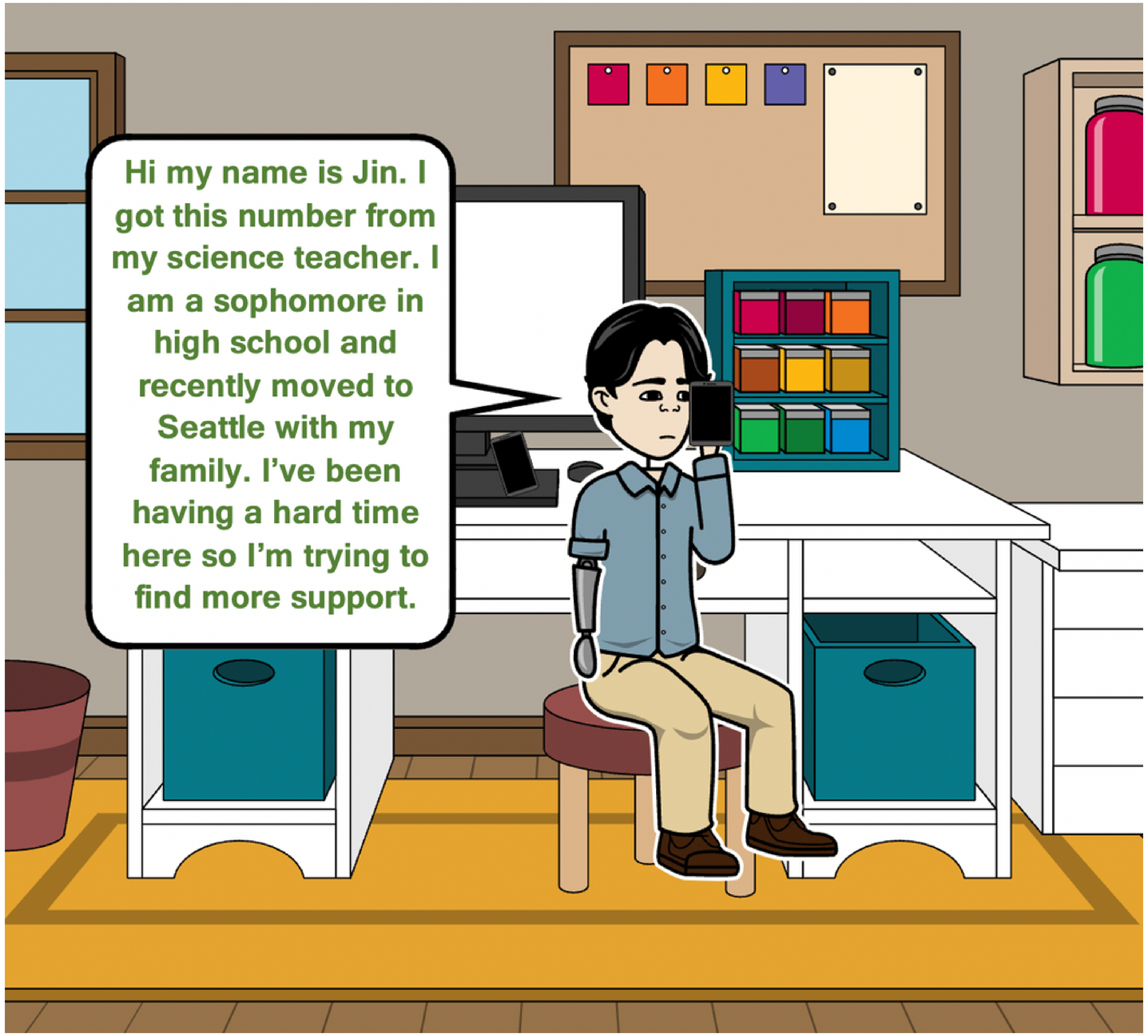
Hi my name is Jin. I got this number from my science teacher. I am a sophomore in high school and recently moved to Seattle with my family. I've been having a hard time here so I'm trying to find more support.

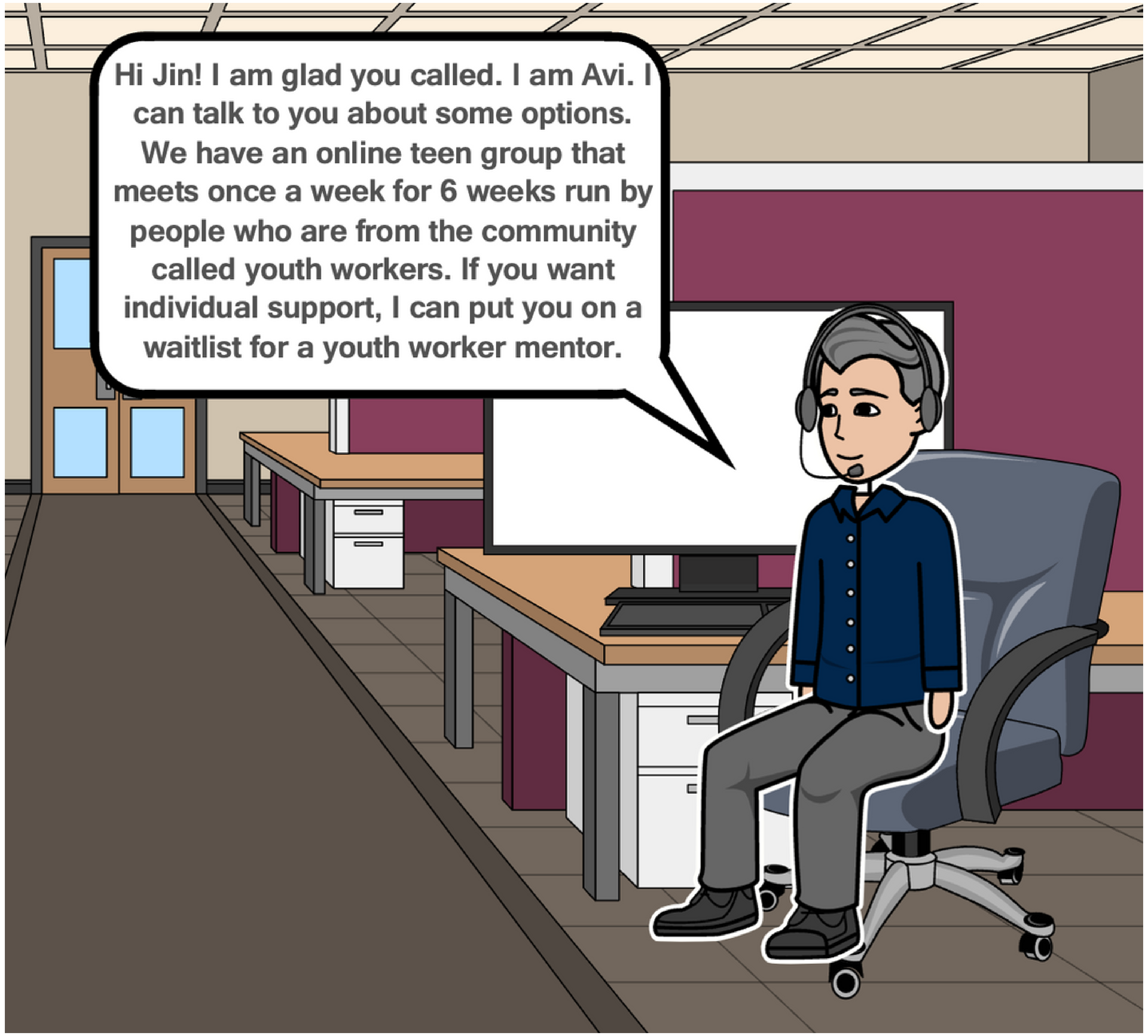
Hi Jin! I am glad you called. I am Avi. I can talk to you about some options. We have an online teen group that meets once a week for 6 weeks run by people who are from the community called youth workers. If you want individual support, I can put you on a waitlist for a youth worker mentor.

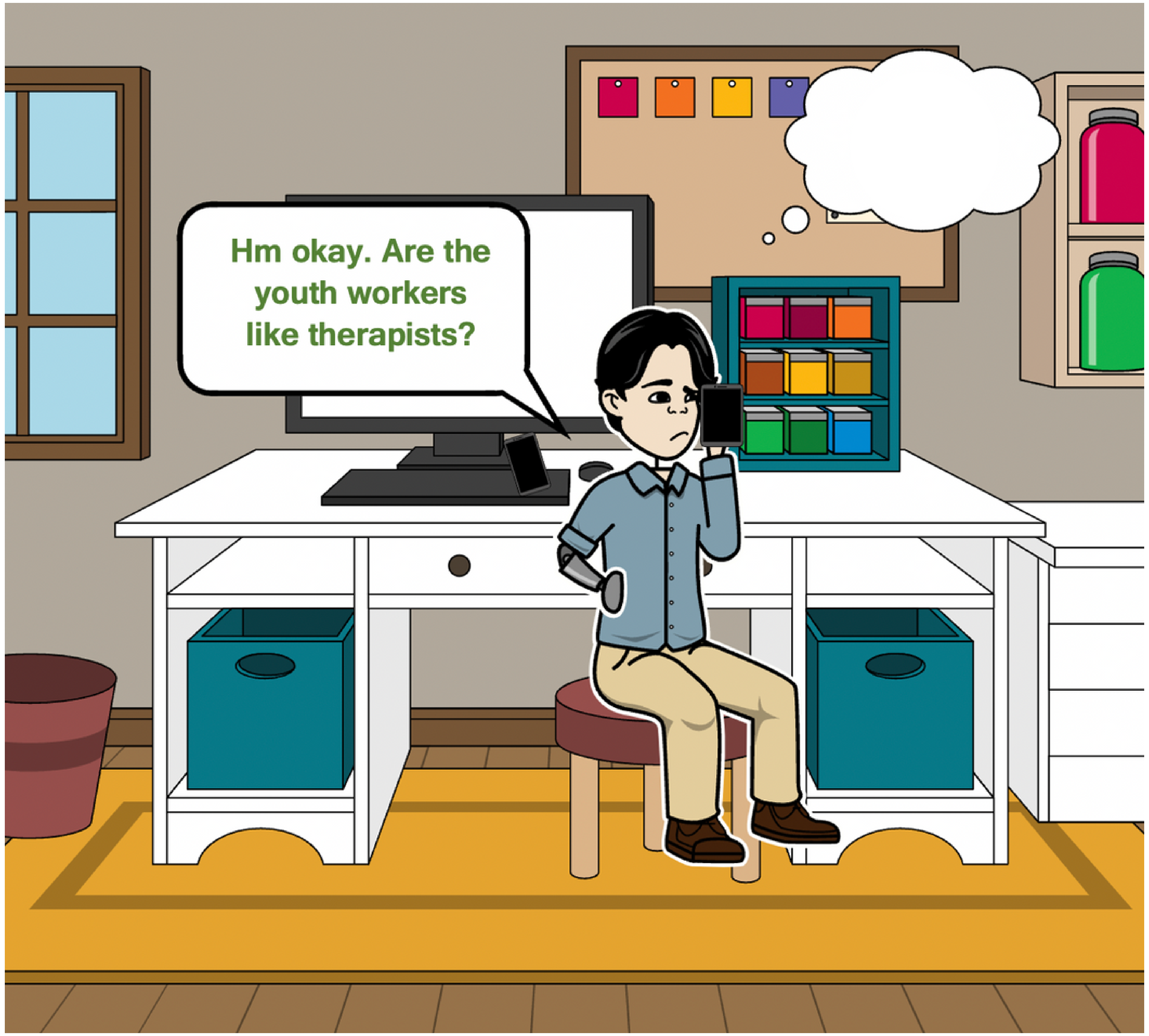
Hm okay. Are the youth workers like therapists?

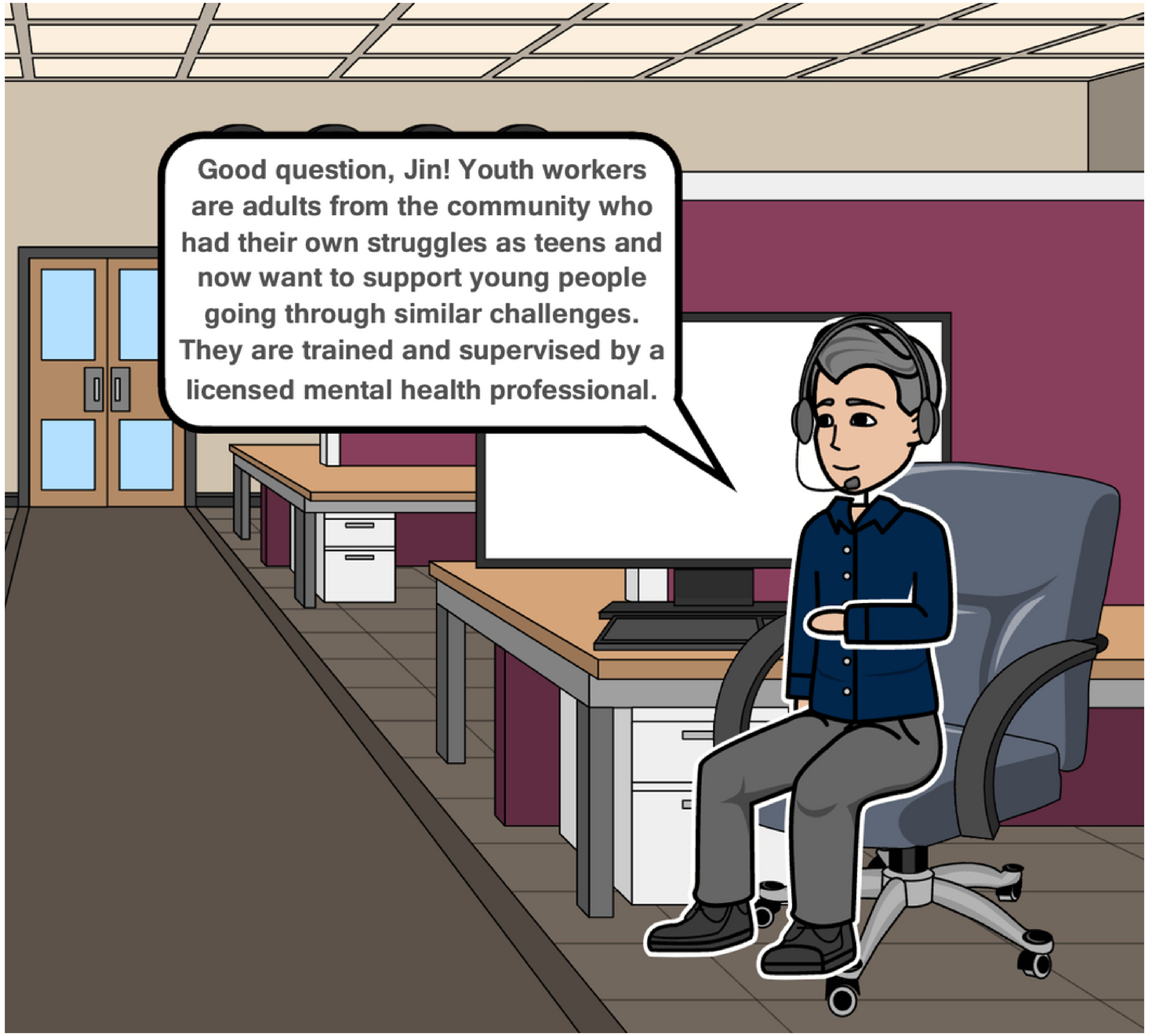
Good question, Jin! Youth workers are adults from the community who had their own struggles as teens and now want to support young people going through similar challenges. They are trained and supervised by a licensed mental health professional.

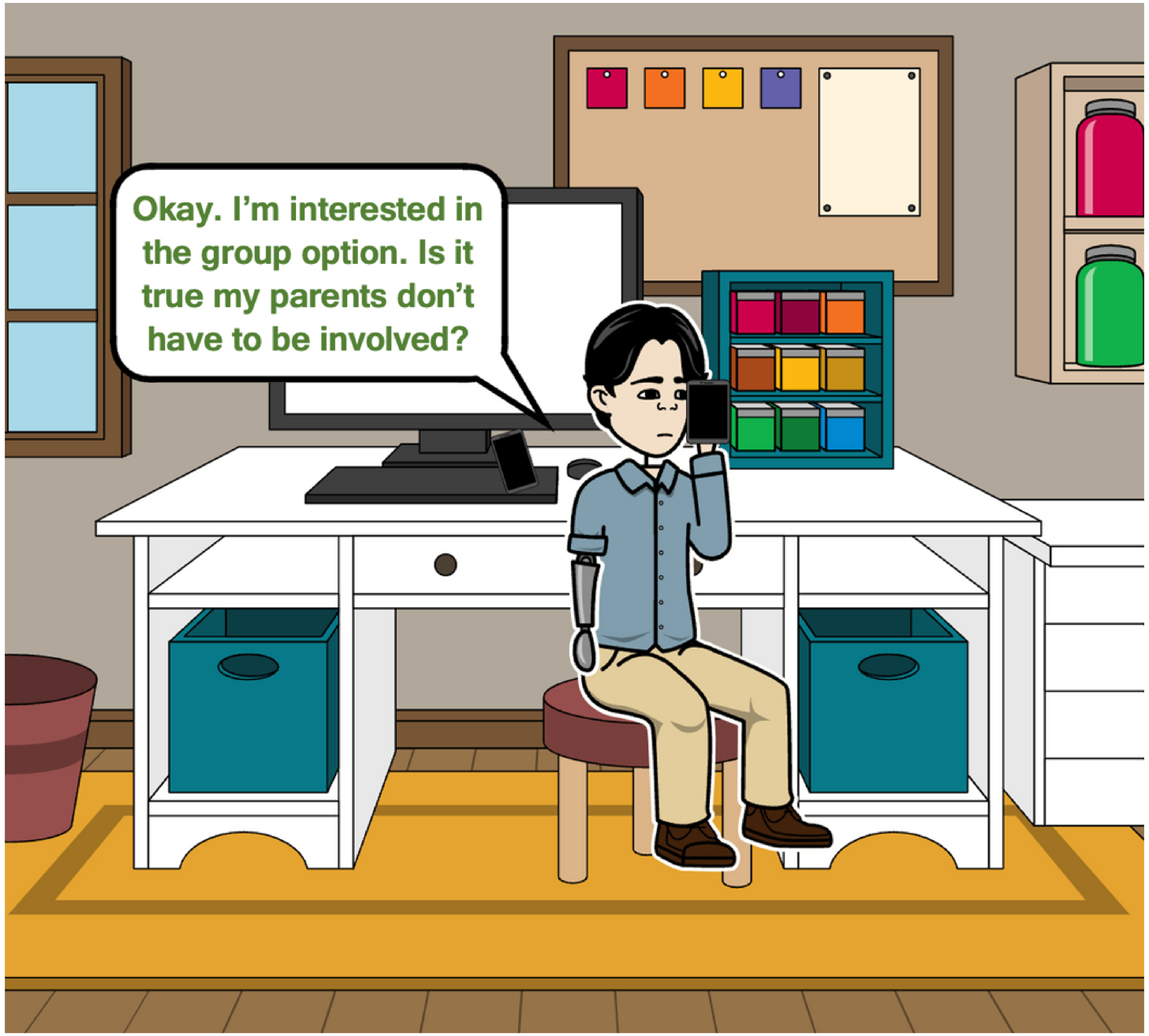
Okay. I'm interested in the group option. Is it true my parents don't have to be involved?

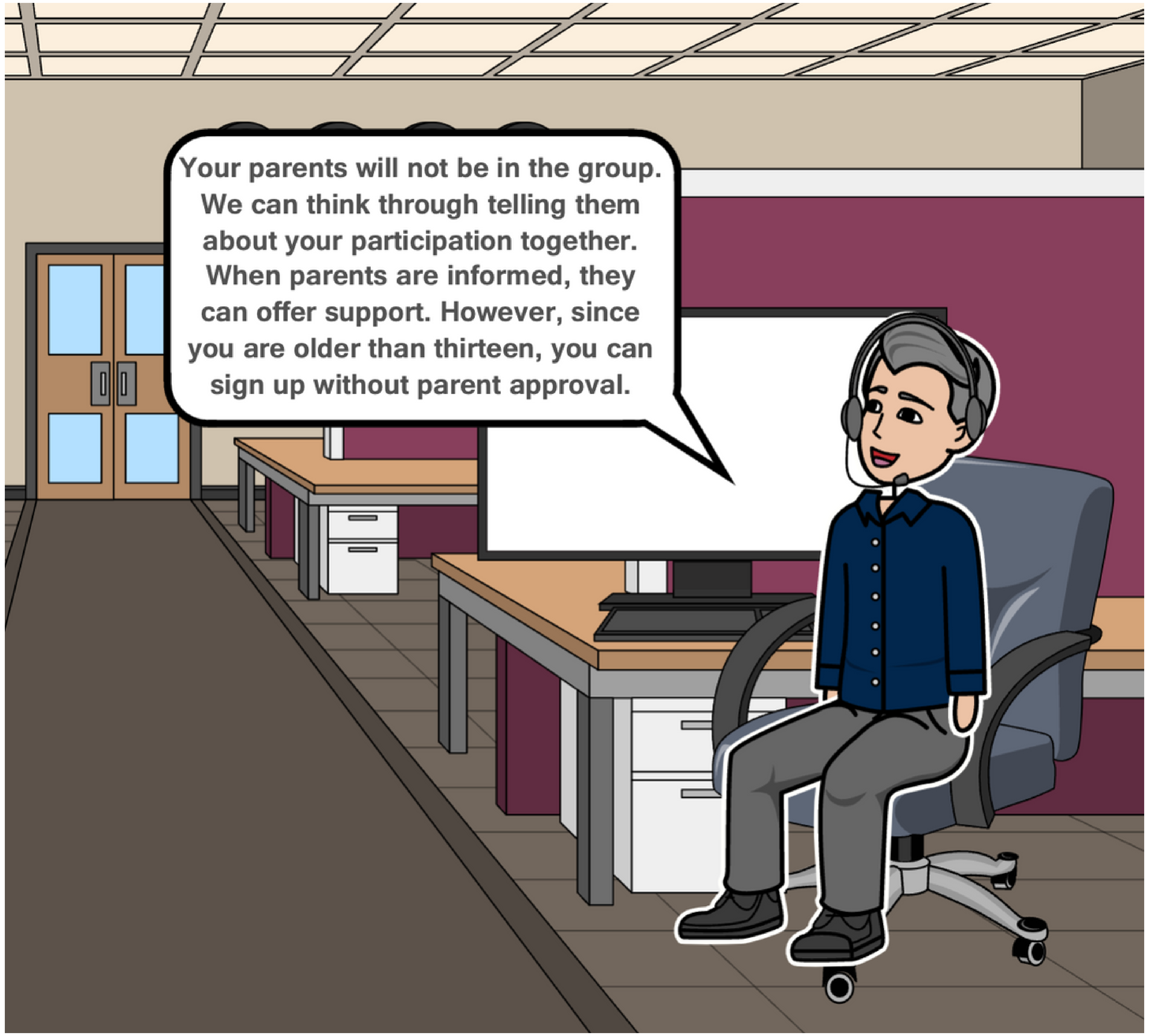
Your parents will not be in the group. We can think through telling them about your participation together. When parents are informed, they can offer support. However, since you are older than thirteen, you can sign up without parent approval.

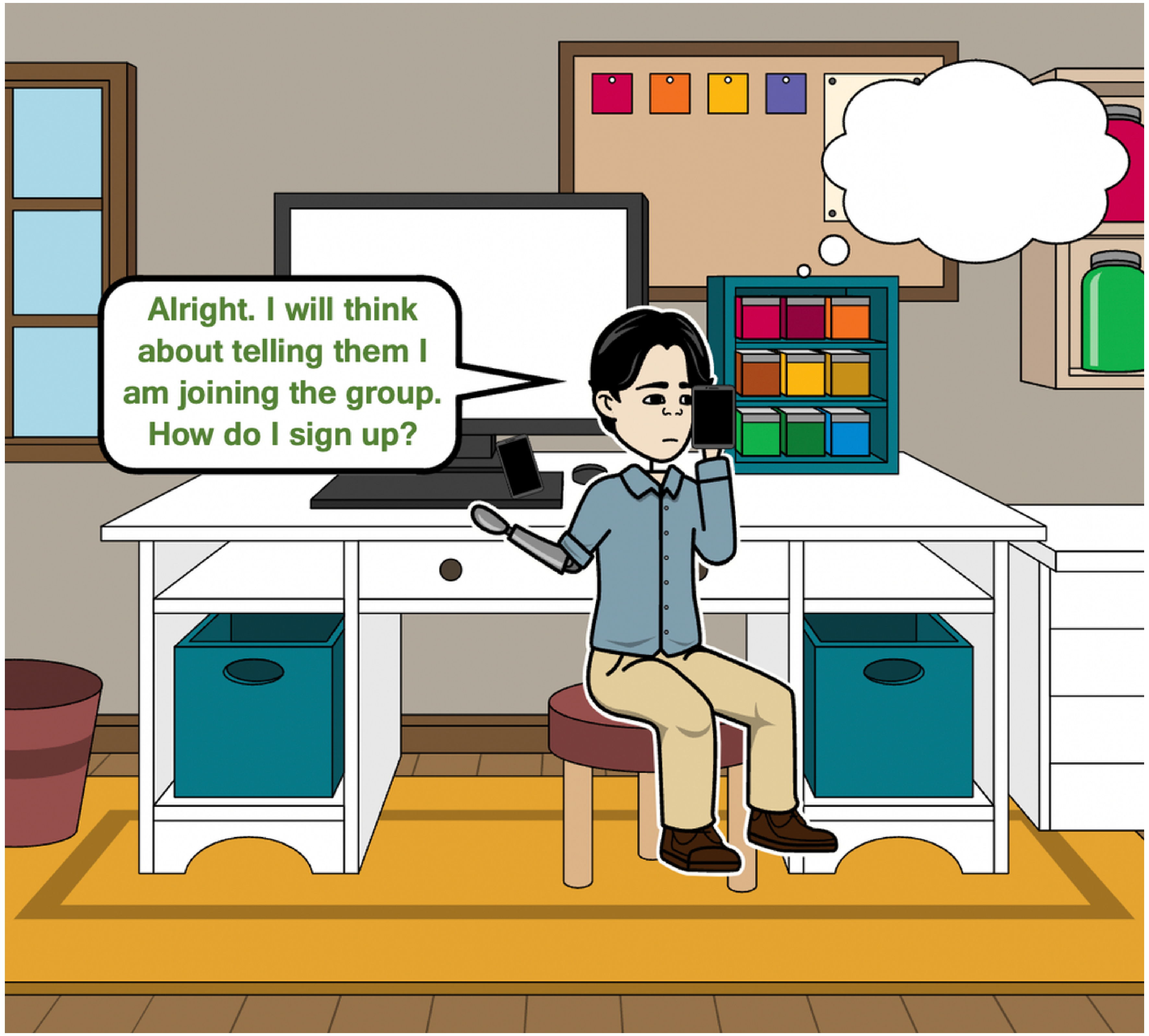
Alright. I will think about telling them I am joining the group. How do I sign up?

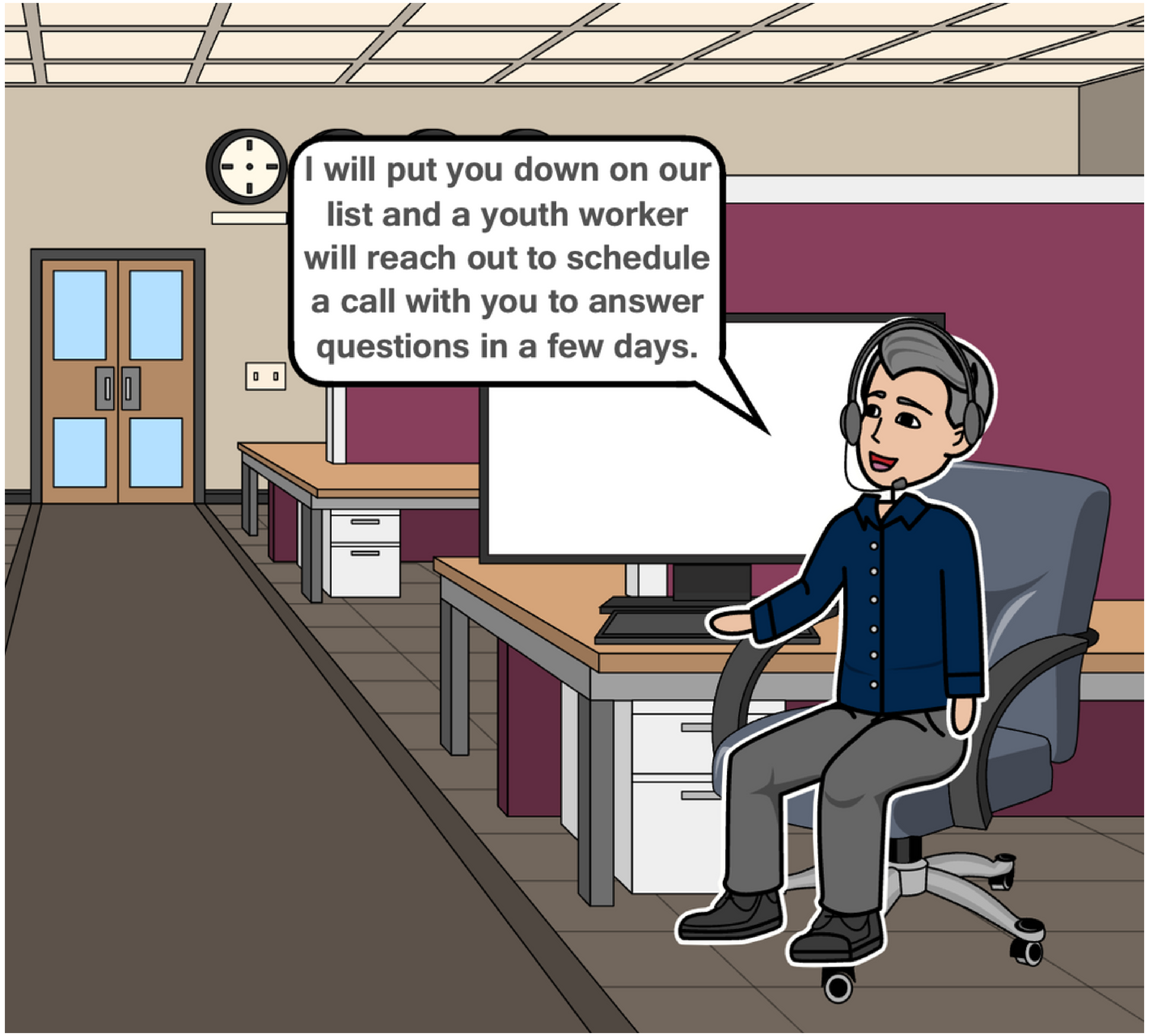
I will put you down on our list and a youth worker will reach out to schedule a call with you to answer questions in a few days.

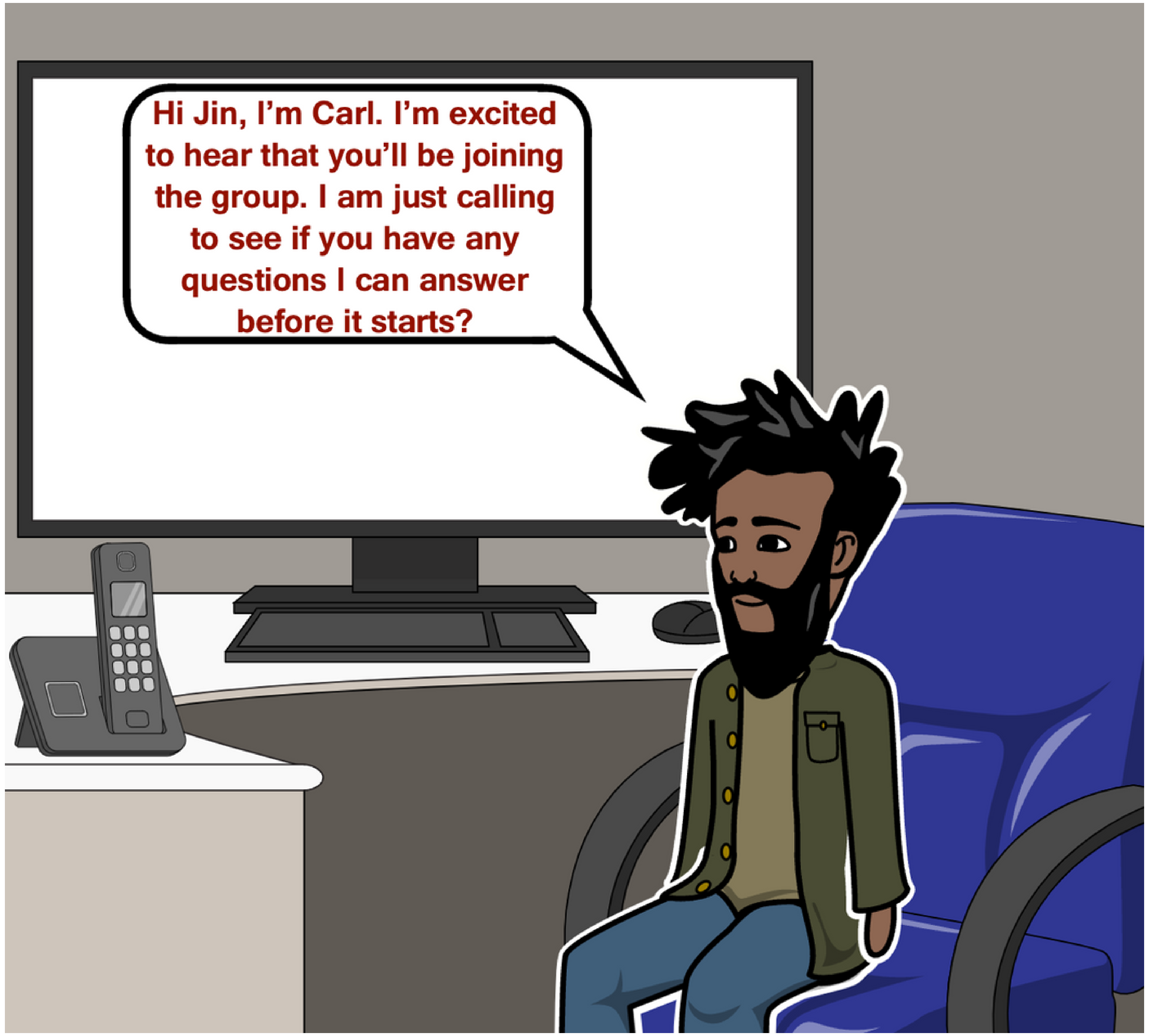
Hi Jin, I'm Carl. I'm excited to hear that you'll be joining the group. I am just calling to see if you have any questions I can answer before it starts?

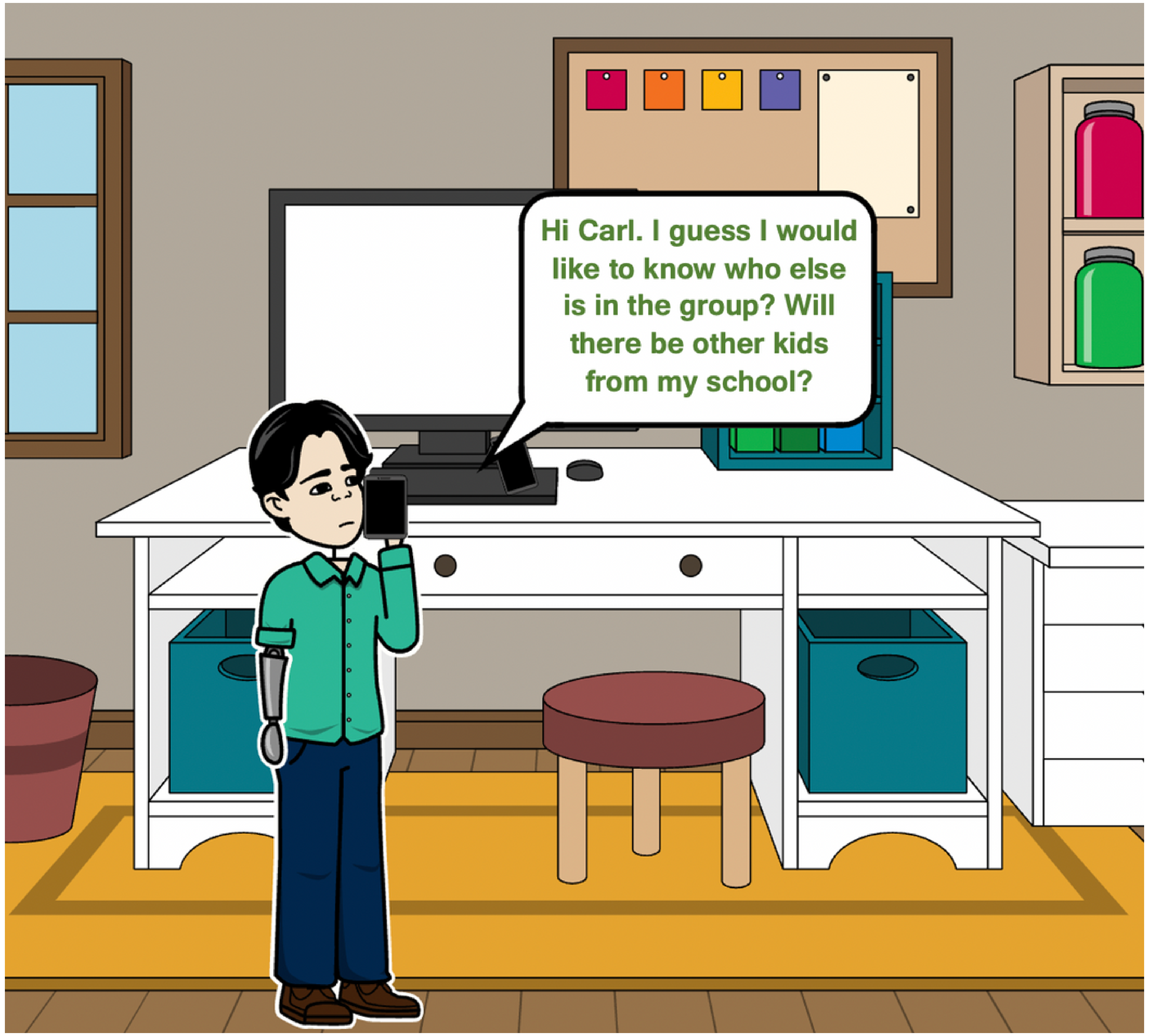
Hi Carl. I guess I would like to know who else is in the group? Will there be other kids from my school?

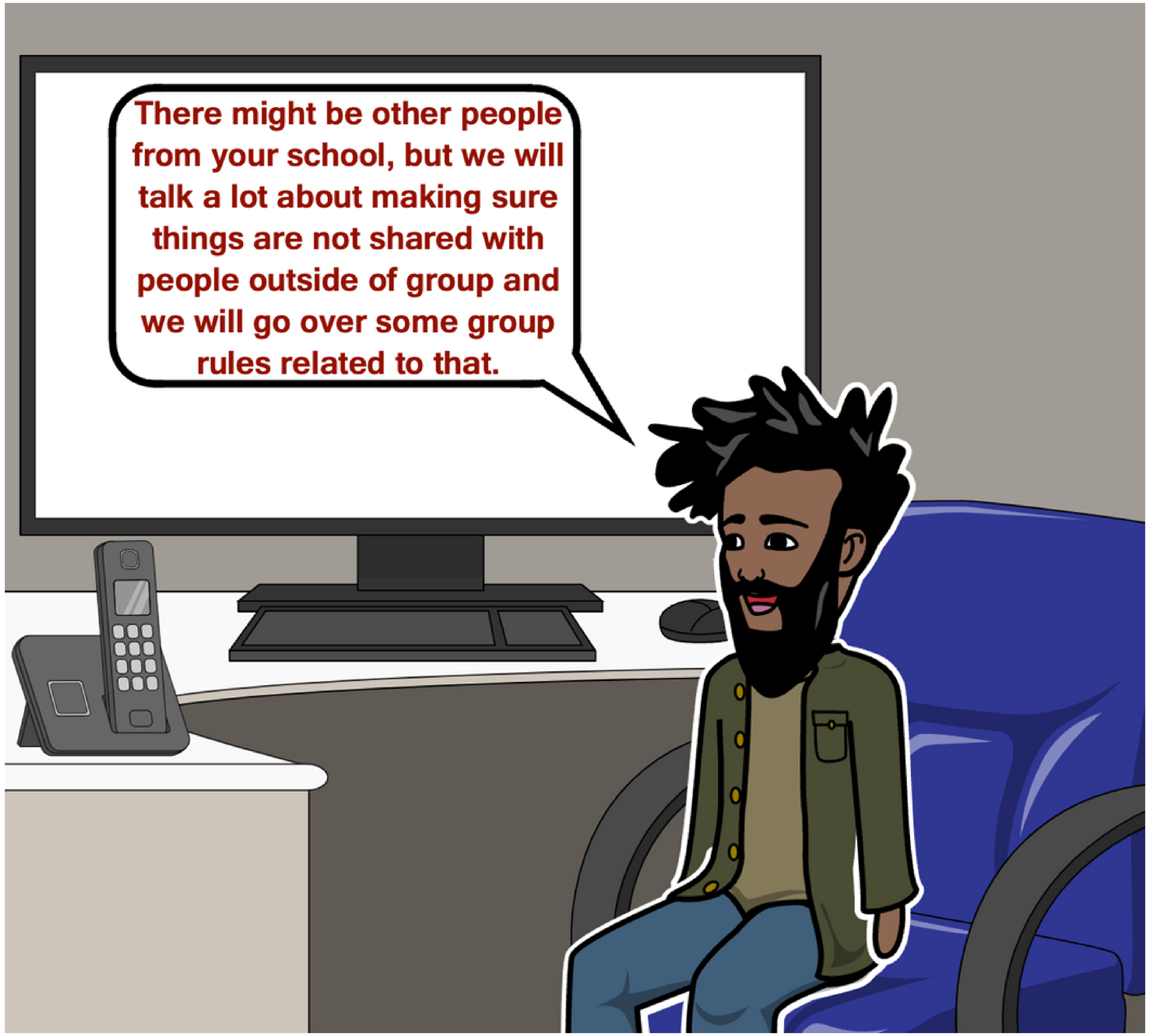
There might be other people from your school, but we will talk a lot about making sure things are not shared with people outside of group and we will go over some group rules related to that.

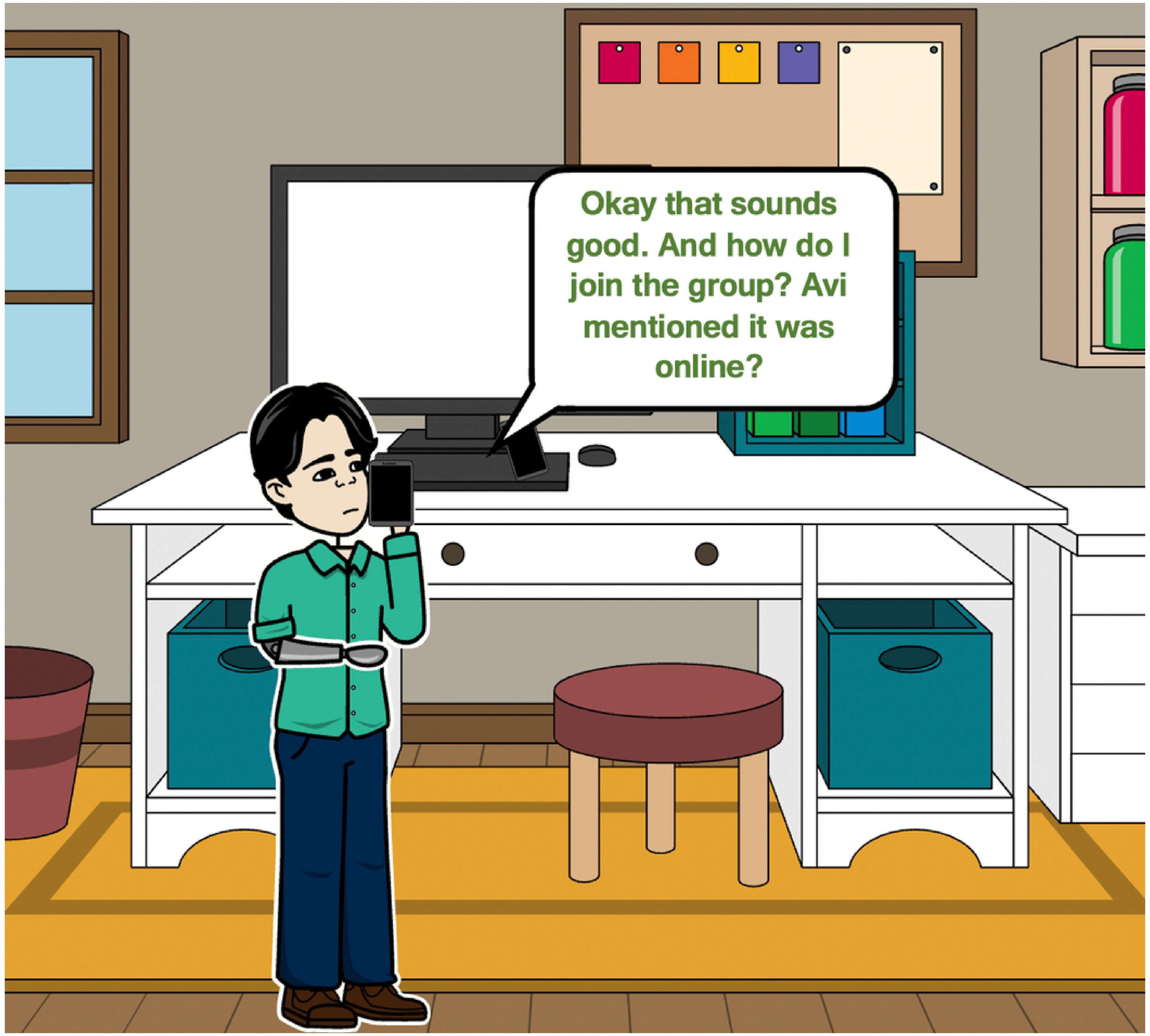
Okay that sounds good. And how do I join the group? Avi mentioned it was online?

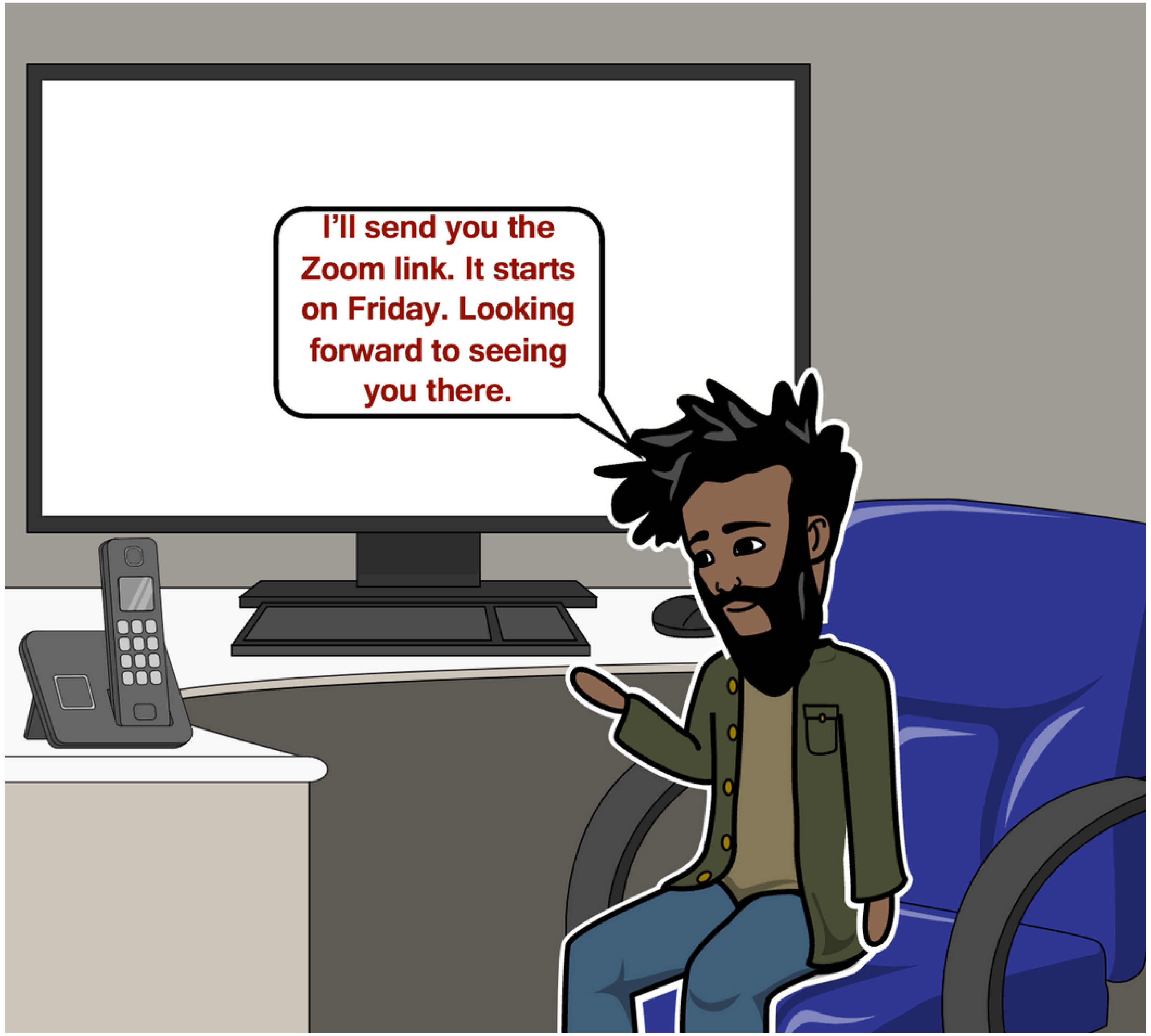
I'll send you the Zoom link. It starts on Friday. Looking forward to seeing you there.

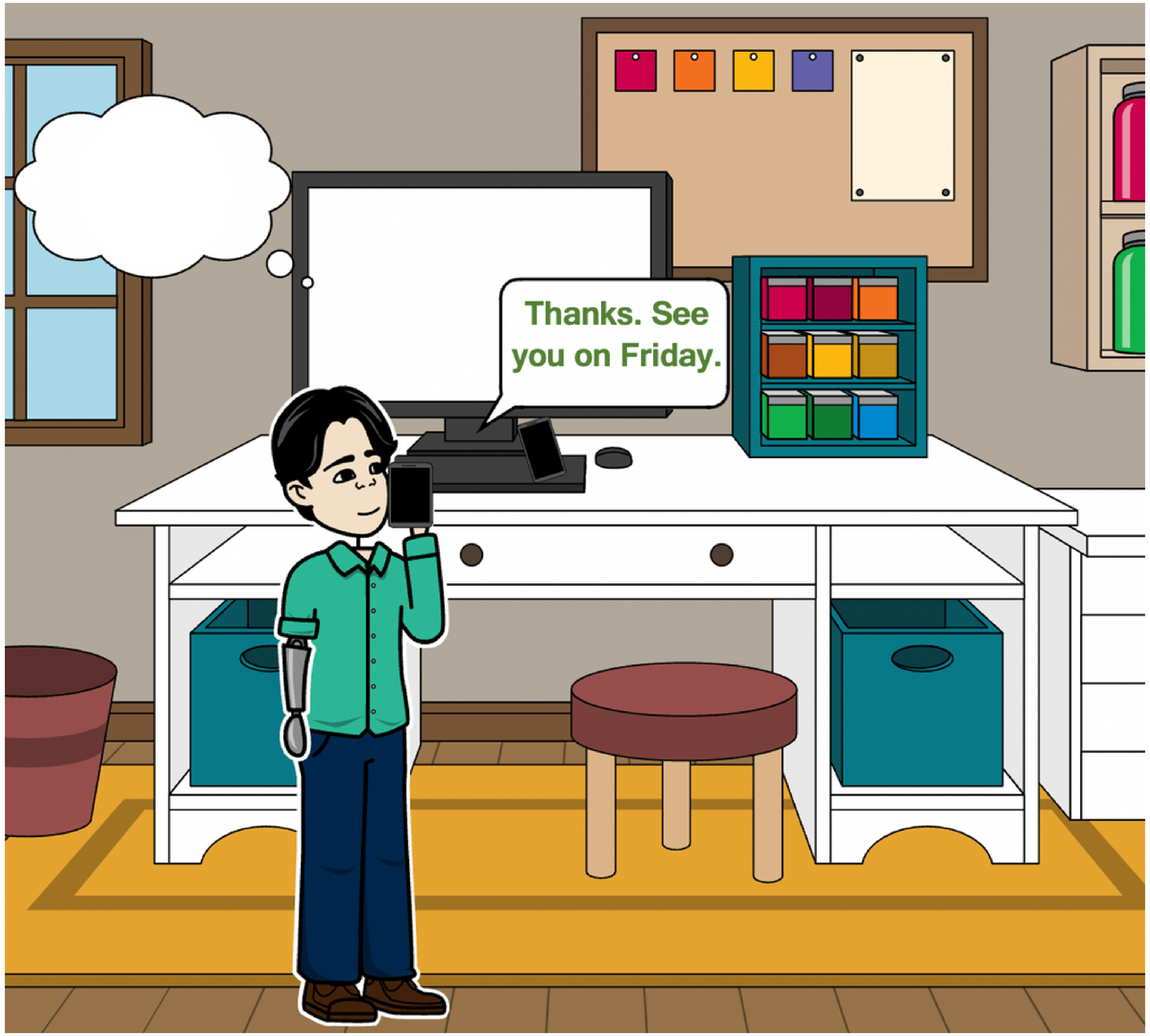
Thanks. See you on Friday.

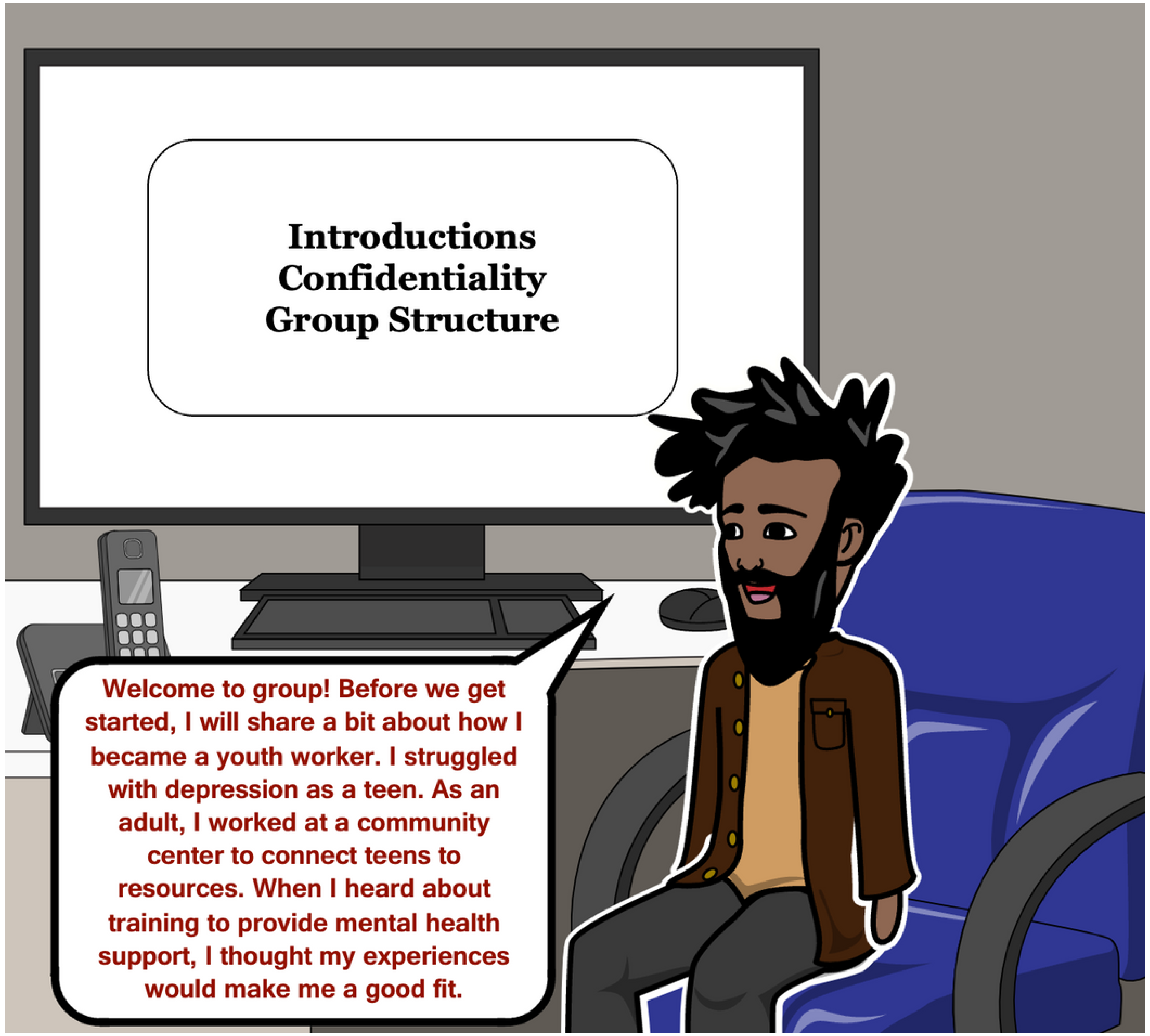
Introductions
Confidentiality
Group Structure
Welcome to group! Before we get started, I will share a bit about how I became a youth worker. I struggled with depression as a teen. As an adult, I worked at a community center to connect teens to resources. When I heard about training to provide mental health support, I thought my experiences would make me a good fit.

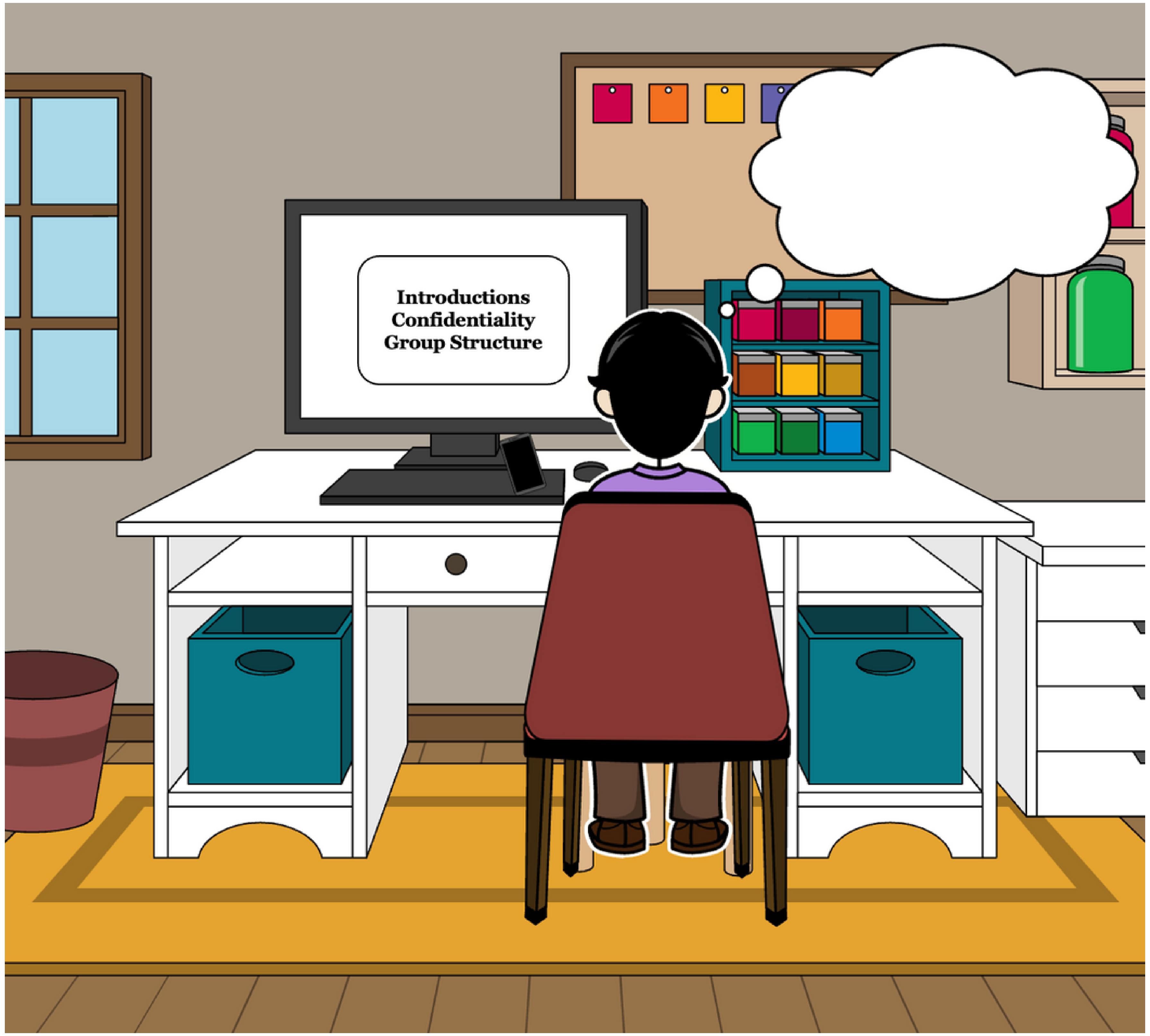
Introductions
Confidentiality
Group Structure

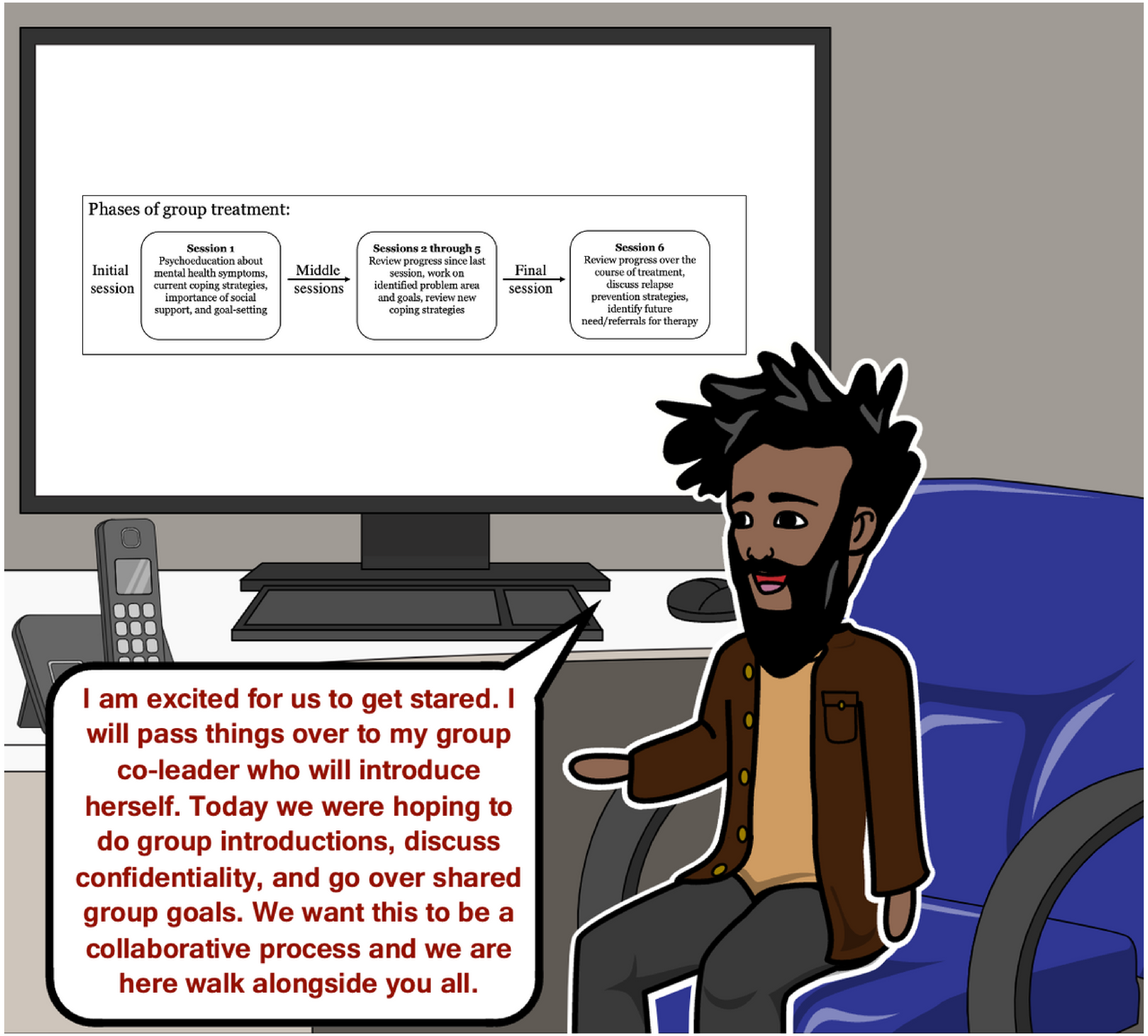
Phases of group treatment:
Initial session
Session 1 Psychoeducation about mental health symptoms, current coping strategies, importance of social support, and goal-setting
Middle sessions
Sessions 2 through 5 Review progress since last session, work on identified problem area and goals, review new coping strategies
Final session
Session 6 Review progress over the course of treatment, discuss relapse prevention strategies, identify future need/referrals for therapy
I am excited for us to get stared. I will pass things over to my group co-leader who will introduce herself. Today we were hoping to do group introductions, discuss confidentiality, and go over shared group goals. We want this to be a collaborative process and we are here walk alongside you all.

# Model of Supervision

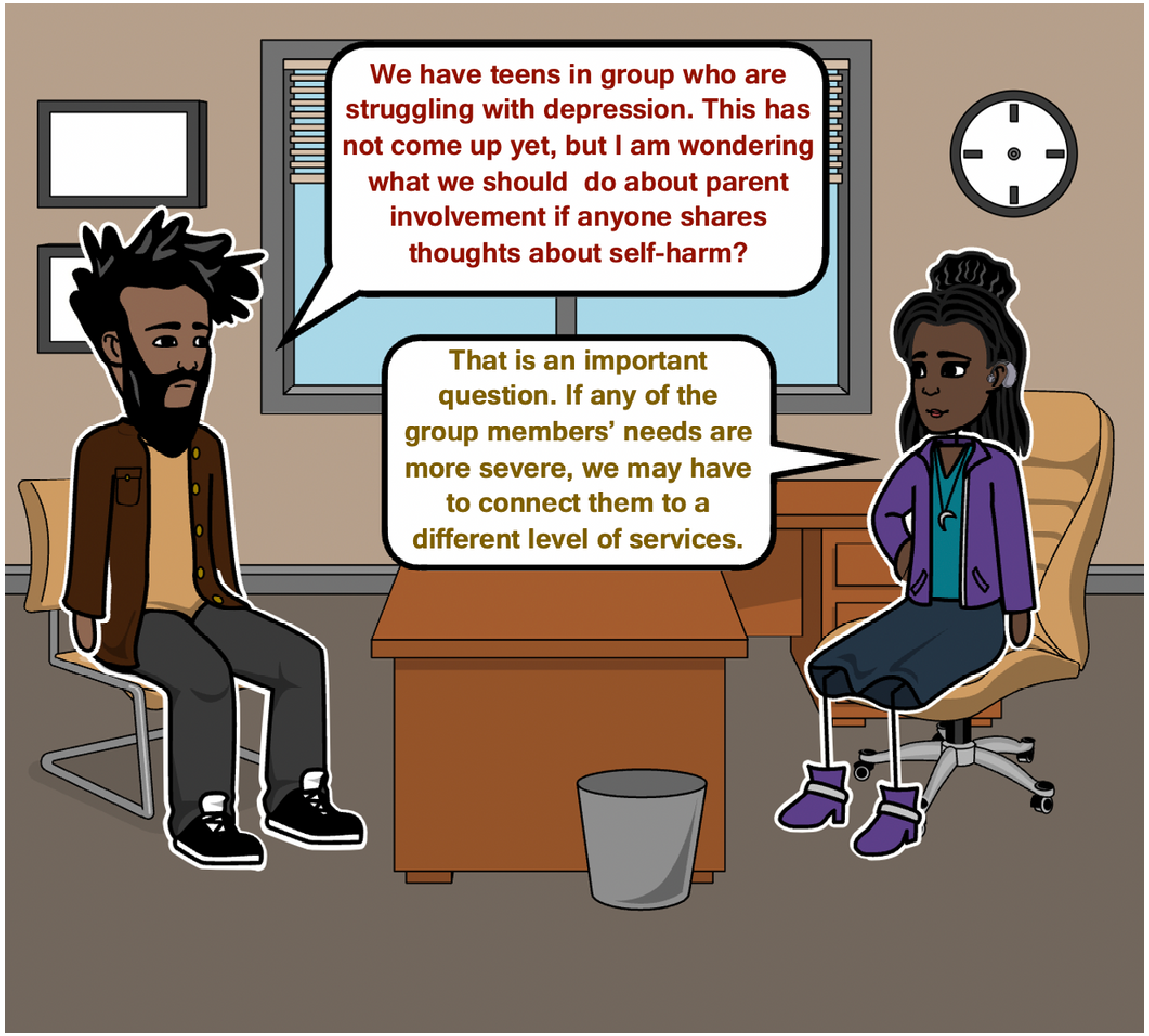


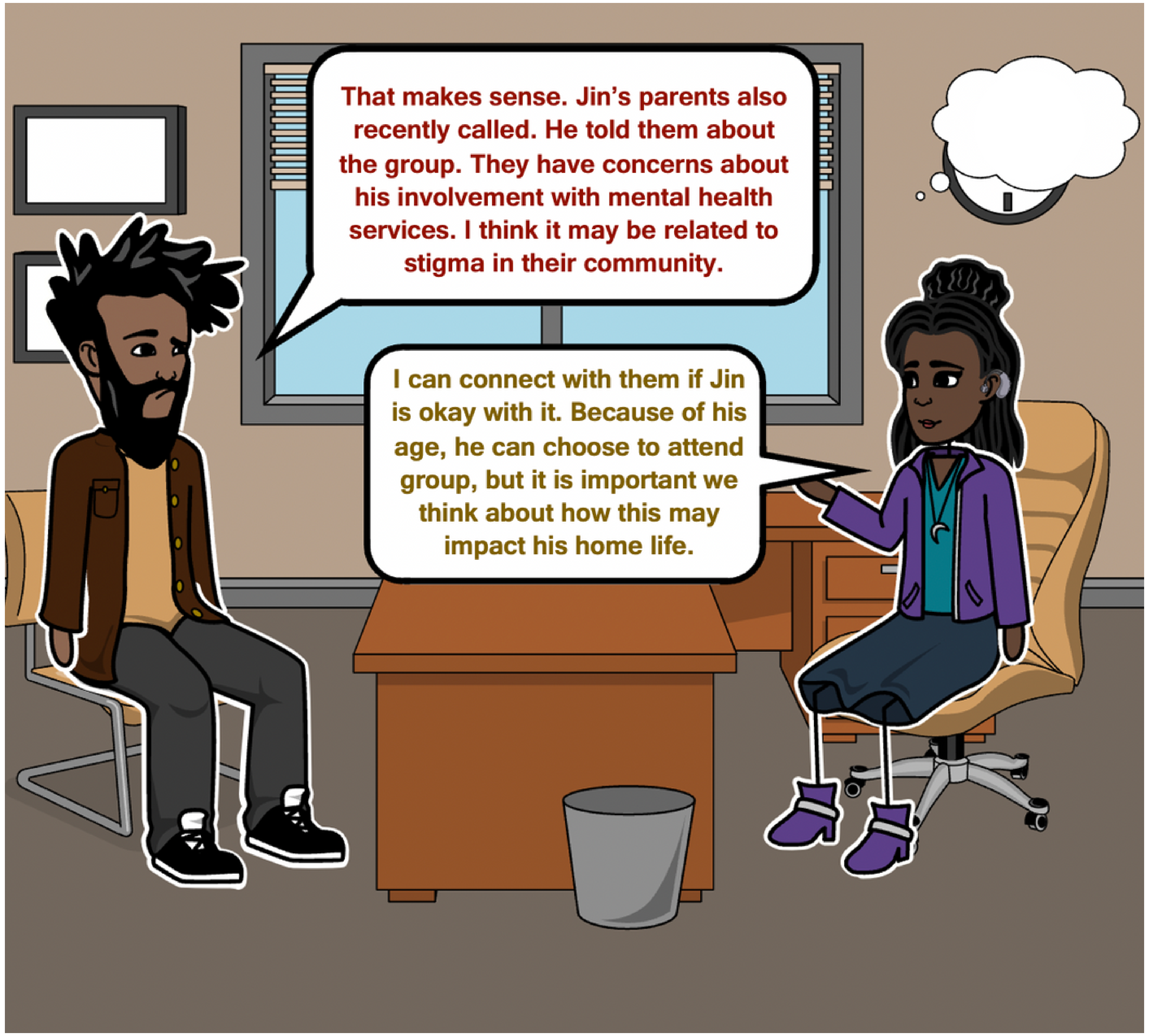

# Individual Youth Worker Model

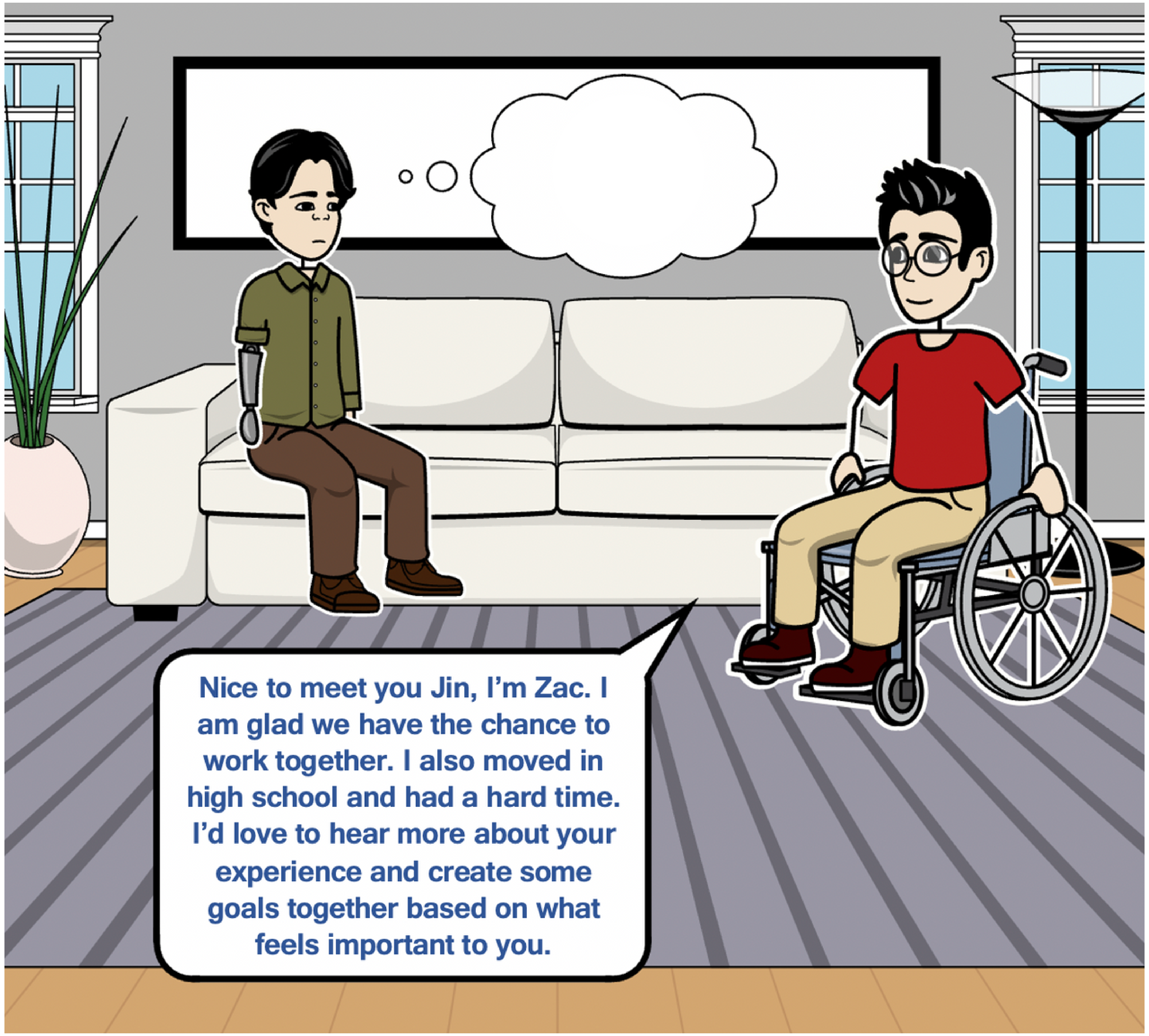

Supplement: Supplementary file 1 [file Data_Sheet_1.PDF]
